# Integrative structural analysis of the type III secretion system needle complex from *Shigella flexneri*

**DOI:** 10.1002/pro.4595

**Published:** 2023-04-01

**Authors:** Lara Flacht, Michele Lunelli, Karol Kaszuba, Zhuo Angel Chen, Francis J. O'. Reilly, Juri Rappsilber, Jan Kosinski, Michael Kolbe

**Affiliations:** ^1^ Department for Structural Infection Biology Center for Structural Systems Biology (CSSB) & Helmholtz Centre for Infection Research (HZI) Hamburg Germany; ^2^ Dynamics of Viral Structures, Leibniz Institute for Virology (LIV) Hamburg Germany; ^3^ Centre for Structural Systems Biology (CSSB) & European Molecular Biology Laboratory (EMBL) Hamburg Germany; ^4^ Technische Universität Berlin, Institute of Biotechnology, Chair of Bioanalytics Berlin Germany; ^5^ University of Edinburgh, Wellcome Centre for Cell Biology Edinburgh UK; ^6^ Structural and Computational Biology Unit, European Molecular Biology Laboratory Heidelberg Germany; ^7^ MIN‐Faculty University Hamburg Hamburg Germany

## Abstract

The type III secretion system (T3SS) is a large, transmembrane protein machinery used by various pathogenic gram‐negative bacteria to transport virulence factors into the host cell during infection. Understanding the structure of T3SSs is crucial for future developments of therapeutics that could target this system. However, much of the knowledge about the structure of T3SS is available only for *Salmonella*, and it is unclear how this large assembly is conserved across species. Here, we combined cryo‐electron microscopy, cross‐linking mass spectrometry, and integrative modeling to determine the structure of the T3SS needle complex from *Shigella flexneri*. We show that the *Shigella* T3SS exhibits unique features distinguishing it from other structurally characterized T3SSs. The secretin pore complex adopts a new fold of its C‐terminal S domain and the pilotin MxiM[SctG] locates around the outer surface of the pore. The export apparatus structure exhibits a conserved pseudohelical arrangement but includes the N‐terminal domain of the SpaS[SctU] subunit, which was not present in any of the previously published virulence‐related T3SS structures. Similar to other T3SSs, however, the apparatus is anchored within the needle complex by a network of flexible linkers that either adjust conformation to connect to equivalent patches on the secretin oligomer or bind distinct surface patches at the same height of the export apparatus. The conserved and unique features delineated by our analysis highlight the necessity to analyze T3SS in a species‐specific manner, in order to fully understand the underlying molecular mechanisms of these systems. The structure of the type III secretion system from *Shigella flexneri* delineates conserved and unique features, which could be used for the development of broad‐range therapeutics.

## Abstract 2

PDB Code(s): 8AXK, 8AXN and 8AXL;

## 1 INTRODUCTION

Increasing antimicrobial resistance is becoming one of the biggest global health threats (Dadgostar, 2019; Ventola, 2015; World Health Organization, 2021), highlighting the need for novel therapeutics. One of the potential targets for such therapeutics is the type III secretion system (T3SS) (Lyons & Strynadka, 2019). The T3SS is used by many pathogenic gram‐negative bacteria, including *Shigella*, *Salmonella*, *Pseudomonas*, *Yersinia*, and Enteropathogenic *Escherichia coli*, to transport virulence factors into the host cell during infection (Galán & Collmer, 1999). It is an attractive target for drug development due to its essential role during infection and accessibility on the cell surface. Yet, much of the underlying molecular mechanisms of the T3SS remain unclear, especially concerning species‐specific differences.

**FIGURE 1:**
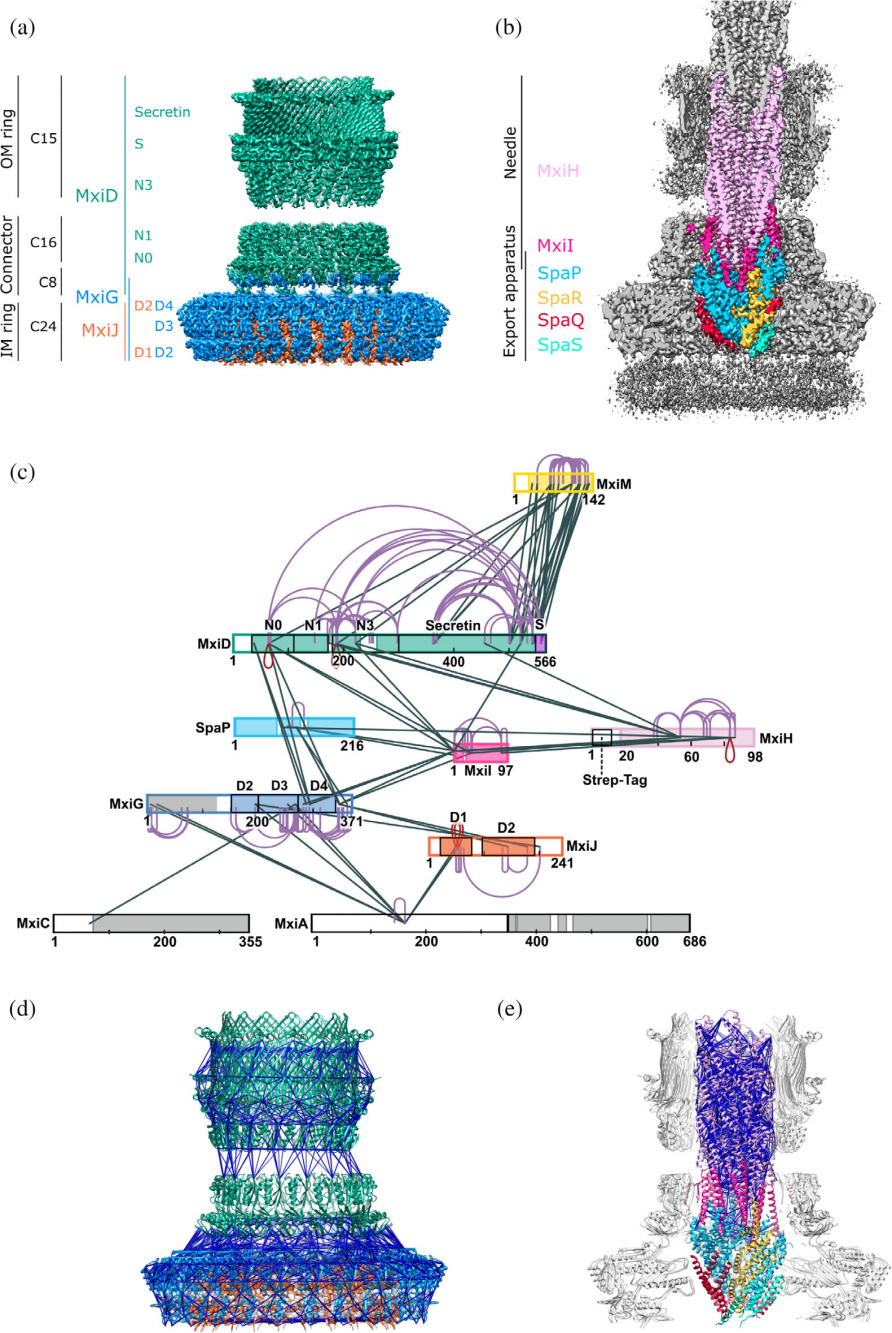
Structural overview of the type 3 secretion system needle complex. (a) Reconstructions of the needle complex regions with different symmetries. Composite map of the reconstructions focused on the needle complex basal body and obtained by applying cyclic symmetry (see also Table 1 and Figure S2). Substructures, symmetry, proteins and their corresponding domains are indicated on the left. The map regions are colored according to the constituting proteins. (MxiJ[SctJ] orange; MxiG[SctJ] medium blue; MxiD[SctC] green). (b) Cross‐section of the cryo‐EM reconstruction of the needle complex. The map was obtained without imposing symmetry and is represented and sliced along the vertical axis. The regions used for the model building of the export apparatus core and the proximal end of the needle are colored according to the constituting proteins as indicated on the left (SpaP[SctR] light blue; SpaQ[SctS] red; SpaR[SctT] dark yellow; SpaS[SctU] turquoise; MxiI[SctF] magenta; MxiH[SctF] pink). (c) Cross‐linking network of the type 3 secretion system proteins. Protein sequences are displayed as bars. Regions that where structurally solved in this study are colored according to panels (a) and (b). Structures available from other studies are indicated in either gray (for MxiA[SctV], MxiC[SctW], and the cytoplasmic domain of MxiG[SctJ]), yellow (for MxiM[SctG]) or purple (S domain of MxiD[SctC]). Heteromeric cross‐links are shown in black, self cross‐links in violet and homo‐multimeric cross‐links in red. (d) and (e) Confirmatory cross‐links mapped onto (d) the basal body rings (MxiG[SctJ], MxiJ[SctJ], and MxiD[SctC]) and (e) the export apparatus including the needle (SpaP[SctR], SpaQ[SctS], SpaR[SctT], SpaS[SctU], MxiI[SctF], and MxiH[SctF]). Structures are shown in the cartoon representation with cross‐links below the distance threshold of 30 Å indicated as dark blue sticks. Cross‐links exceeding this threshold are shown in Figure S7.

The T3SS is a large, syringe‐shaped, membrane‐spanning, multimegadalton protein machinery. It is composed of the membrane‐embedded needle complex (Figure 1a, b) and cytosolic components such as the sorting platform and effector proteins with their corresponding chaperones, overall comprising more than 20 proteins (Galán et al., 2014). The needle complex includes the membrane‐spanning basal body and a hollow filamentous needle protruding from the bacterial surface (Blocker et al., 2001). While the needle is a helical complex composed of a single protein (Figure 1b) (MxiH in *Shigella* [SctF in the unified nomenclature; Hueck, 1998]) (Demers et al., 2013), the basal body comprises multiple different proteins, either forming membrane‐associated periplasmic rings (Figure 1a) (MxiD[SctC], MxiG[SctD], MxiJ[SctJ] in *Shigella*) or the pseudohelical structure of the export apparatus core (Figure 1b) (SpaP[SctR], SpaQ[SctS], SpaR[SctT], and SpaS[SctU] in *Shigella*; Blocker et al., 2001; Lunelli et al., 2020).

To this end, several high‐resolution cryo‐electron microscopy (cryo‐EM) structures from single‐particle analysis of needle complexes have been solved (Goessweiner‐Mohr et al., 2019; J. Hu et al., 2018, 2019; Miletic et al., 2021) but all originated from the same pathogen—*Salmonella*. Recently, we could solve the partial cryo‐EM needle complex structure from another genus, *Shigella*. However, the insufficient resolution of some regions did not allow de novo modeling substantial areas.

First, the resolution of the outer membrane (OM) ring only allowed for building its structure based on homology model of the corresponding ring from *Salmonella* typhimurium pathogenicity island 1 (SPI‐1) T3SS and lacked information on its assembly‐relevant, C‐terminal S domain. The OM ring (MxiD [SctC]) belongs to the family of secretins, which are pore‐forming proteins found within the T2SS, T3SS, and in the type IV pilus system (T4PS), and assemble into homo‐oligomeric β‐barrels (Majewski et al., 2018). In the T3SS, the N‐terminal subdomains N0 and N1 form the connector, and the N3 together with the secretin and S domain compose the OM ring (Figure 1a). Interestingly, the connector and the OM ring show a different symmetry (C16 vs. C15), suggesting that one subunit is either proteolytically cleaved between the N1 and N3 domains or its N3, secretin and S domains are extruded from the OM ring and adopt a flexible conformation (Goessweiner‐Mohr et al., 2019; Hu et al., 2019; Lunelli et al., 2020). While most of the domains are highly conserved (even among the different secretion systems), the S domains share no sequence similarity whatsoever (Lunelli et al., 2020; Worrall et al., 2016; Yan et al., 2017). In *Salmonella*, which is the only high‐resolution structure of a T3SS secretin currently available, the S domain interacts with a neighboring subunit, stabilizing the pore‐complex as a “molecular clamp” (Hu et al., 2018; Worrall et al., 2016). However, it is unclear how the S domain contributes to the pore stabilization in *Shigella*.

Additionally to the stabilization, the S domain acts as a binding site for the pilot protein, on which some secretins depend for complex assembly and/or transport to the OM (Koo et al., 2012; Okon et al., 2008; Schuch & Maurelli, 2001). Pilotins are a diverse group of small proteins that have an N‐terminal signal sequence with a conserved lipidated cysteine residue, by which they are thought to be transported to the OM via the lol‐pathway (Koo et al., 2012; de Silva et al., 2020). They differ significantly in structure (de Silva et al., 2020), ranging from mostly α‐helical (e.g., InvH[SctG] in *Salmonella*; Majewski et al., 2021) to being composed primarily out of β‐sheets (e.g., MxiM[SctG] in *Shigella*; Lario et al., 2005). Up to date, only a single fully assembled secretin structure from the *E. coli* T2SS (GspD) was solved in complex with its corresponding pilotin (AspS), revealing a 15:15 stoichiometry (Yin et al., 2018). It is unclear why some pilotins remain bound to secretin after assembly of the pore, while others seem to be released at the end of this process. It was found that MxiM[SctG] co‐purifies with isolated *Shigella* needle complexes (Sani et al., 2007; Zenk et al., 2007) and it was assumed in spike‐like structures around the OM ring (Sani et al., 2007) of the isolated needle complex as well as in an unassigned density from cryo‐ET of *Shigella* minicells (Hu et al., 2015).

Second, the export apparatus (Figure 1b) which serves as the entry gate for substrates (Miletic et al., 2021; Wagner et al., 2010) could only be partially resolved in our previous needle complex structure. However, several three‐dimensional structures have been analyzed from either within isolated T3SS needle complexes (PDB IDs: 6PEM, 6PEP; Hu et al., 2019), 7AGX, 7AH9, 7AHI (Miletic et al., 2021) or from heterologously expressed and subsequently isolated complexes of the T3SS (6R6B; Johnson et al., 2019) and the functionally related bacterial flagellar system (PDB IDs: 6F2D; Kuhlen et al., 2018), 6R69 (Johnson et al., 2019), 6S3L, 6S3S, 6S3R (Kuhlen et al., 2020). They all share a conserved pseudohelical arrangement, with a stoichiometry of 5:4:1 (in *Shigella* SpaP[SctR]:SpaQ[SctS]:SpaR[SctT]) being the most common. Yet, a structure including the autoprotease (SpaS[SctU] in *Shigella*), which cleavage regulates secretion hierarchy from middle to late effectors proteins (Deane et al., 2008; Sorg et al., 2007), has only been described for the flagellar homolog, FlhB[SctU], from *Vibrio* (Kuhlen et al., 2020).

Lastly, the previous corresponding electron density maps did not allow for visualizing interactions between some substructures. This included the detailed interfaces between the export apparatus (Figure 1b) and the inner membrane (IM) ring (Figure 1a). Furthermore, it remained unclear how the inner rod protein MxiI[SctI] connects the export apparatus with the needle (Figure 1b) and connector (Torres‐Vargas et al., 2019) (Figure 1a). Additionally, we could not determine the location and conformation of the C‐terminal domain of one‐third of the MxiG[SctD] subunits (Figure 1a).

To fill these gaps in the structural knowledge of T3SS in *Shigella* and to understand the extent of intergenera differences in T3SSs, we improved the cryo‐EM reconstructions of the *Shigella* needle complex. We report comprehensive de novo atomic models revealing (i) the structure of the OM secretin (MxiD[SctC]) pore and the unique fold of its C‐terminal S domain (ii) the periplasmic components of the export apparatus (SpaP[SctR], SpaQ[SctS], SpaR[SctT]) including the SpaS[SctU] subunit, unresolved in any of the previous virulence‐related T3SS structures, (iii) the proteins interfacing the export apparatus with the hollow needle (mxiI[SctI] and MxiH[SctF]), and (iv) the conformation of the IM ring subunits (MxiG[SctD]) at the interface with the connector (MxiD[SctC]). In addition, an integrative modeling approach combining cryo‐EM and cross‐linking mass spectrometry (MS) allowed the docking of the pilotin subunits (MxiM[SctG]) around the secretin ring, a feature apparently unique to *Shigella* T3SS. Overall, this study contributes to a better understanding of the T3SS of *S. flexneri* and enables the structural comparison between two needle complexes from species from two different genera, providing valuable knowledge of differences and similarities, which could help to combat pathogens that utilize the T3SS in the future.

## 2 RESULTS

### 2.1 The architecture of the *Shigella* needle complex as obtained with cryo‐electron microscopy

We reprocessed with Relion 3.0 the data presented in (Lunelli et al., 2020), after removing from the micrographs the last frame, which is the most exposed and suffered of radiation damage. The three‐dimensional reconstruction of the needle complex without imposing symmetry yielded a cryo‐EM map with an overall resolution of 4.05 Å according to the gold‐standard Fourier shell correlation (FSC) (Table 1, Figures S1, S2). The IM ring, connector, and OM ring show 24‐, 16‐, and 15‐fold symmetry, respectively, with a common rotation axis coinciding with the central channel of the needle complex (Figure 1a). Focused refinements imposing local symmetries increased the resolution for the IM ring, connector and their interface (Table 1, Figures S1, S2). Furthermore, partial signal subtraction with focused refinement resulted in an OM ring map at near‐atomic resolution showing defined density for the N3, the secretin, and the S domains of the pentadecameric MxiD[SctC] oligomer (Table 1 and Figures S1–S3), which was not clearly resolved in the previously published *Shigella* maps (Lunelli et al., 2020). Regions inside the needle complex, which do not exhibit any symmetry, could be resolved up to 3.6 Å in a map without imposed symmetry (“C1 map”), enabling us to build the de novo atomic model of the proximal needle end and the export apparatus (Figures 1b, S2). Compared to our previous study (Lunelli et al., 2020), the current structural analysis provides atomic models of the unresolved or partially resolved subunits of the *Shigella* needle complex, including MxiG[SctD], MxiJ[SctJ], MxiD[SctC], MxiI[SctI], MxiH[SctF], and the export apparatus proteins SpaP[SctR], SpaQ[SctS], SpaR[SctT], SpaS[SctU] (Figure S4).

**TABLE 1:** Maps and models refinement data.

Map	Full needle complex	OM ring	IM ring + connector	IM ring
EMDB ID	EMD‐15700	EMD‐15701	EMD‐15702	EMD‐15703
Symmetry	C1	C15	C8	C24
Resolution (FSC = 0.143) (Å)	4.05	3.42	3.34	3.21
Pixel size, Å	1.38	1.38	1.38	1.38
Particle number	90,547	69,589	90,547	90,547
Defocus range (μm)	1.5–4	1.5–4	1.5–4	1.5–4
Map sharpening B factor	−103	−108	−90	−100
Model	Inner components	Secretin pore	IM ring + connector	
PDB ID	8AXK	8AXL	8AXN	
Proteins (domains)	SpaP[SctR], SpaQ[SctS], SpaR[SctT], SpaS[SctU] (N‐term), MxiI[SctI], MxiH[SctF], MxiD[SctC] (N1), MxiJ[SctJ]_90–106_	MxiD[SctC] (N3, secretin, S)	MxiJ[SctJ] (D1, D2), MxiG[SctD] (D2, D3, D4, C‐term), MxiD[SctC] (N0, N1)	
Residues	5608	4800	11,704	
Nonhydrogen atoms	44,141	37,650	94,656	
R.m.s. deviations				
Bond lengths (Å)	0.002	0.006	0.003	
Bond angles (deg)	0.43	0.63	0.51	
Average B‐factor	59.2	72.8	67.0	
Resolution (FSC = 0.5) (Å)	4.1	3.5	3.5	
Clashscore	6.19	9.50	6.77	
Poor rotamers (%)	3.27	3.47	3.52	
Ramachandran plot				
Favored (%)	95.02	93.87	94.57	
Allowed (%)	4.98	6.13	5.43	
Disallowed (%)	0.00	0.00	0.00	
Molprobity score	2.08	2.32	2.16	
EMRinger score	1.22	1.92	2.70	

### 2.2 Cross‐linking mass‐spectrometry of the isolated needle complex reveals additional interactions

To validate the obtained structures and identify interactions between subunits and domains not resolved in the cryo‐EM maps, we performed cross‐linking MS analysis of the T3SS needle complex purified from *Shigella* strain M90T. Homo bifunctional bis(sulfosuccinimidyl)suberate (BS3), predominantly reactive to amino groups of lysine residues and protein N‐termini, was used as a cross‐linker. The reaction conditions were optimized to acquire a sufficient cross‐linking rate and minimize unspecific particle aggregation (Figure S5). In total 364 cross‐links were identified with a 2% false discovery rate (FDR) at linkage level (Fischer & Rappsilber, 2017; Mendes et al., 2019) (Table S1 and Figure S6). For modeling, cross‐links with a search score below 6 or cross‐links involving the MxiH[SctF] purification tag were excluded. With this additional filtering, we obtained 68 heteromeric, 24 homomultimeric, and 132 self‐links. The resulting cross‐linking map (Figure 1c) shows a dense network of connections between needle complex components, mainly involving the needle (MxiH[SctF]), the inner rod (MxiI[SctI]) and the connector/OM ring (MxiD[SctC]), the IM ring proteins (MxiG[SctD] and MxiJ[SctJ]), and also the export apparatus components (SpaP[SctR]). Among proteins not yet included in the structural model, the pilotin (MxiM[SctG]), the gatekeeper (MxiC[SctW]), and the export gate (MxiA[SctV]) are cross‐linked to specific needle complex subunits. In total, 106 cross‐links could be mapped to residues resolved in the cryo‐EM structure, with 90 of them (85%) satisfying the maximal CαCα distance of 30 Å as expected for the BS3 cross‐linker (Figures 1d, e S6a). Most of the violated cross‐links are within MxiD[SctC] and consistently connect secretin and connector domains (Figures S6b, S7), suggesting the presence of another, more compact conformation of this protein. We can, however, not rule out that the violated cross‐links could have been caused by false positive identification or unspecific protein aggregation. Taken together, the cross‐linking MS analysis corroborates the structure obtained with cryo‐EM and indicates the presence of further interactions, not yet resolved structurally.

## 3 THE SECRETIN EXHIBITS A NOVEL S DOMAIN CONFORMATION

**FIGURE 2:**
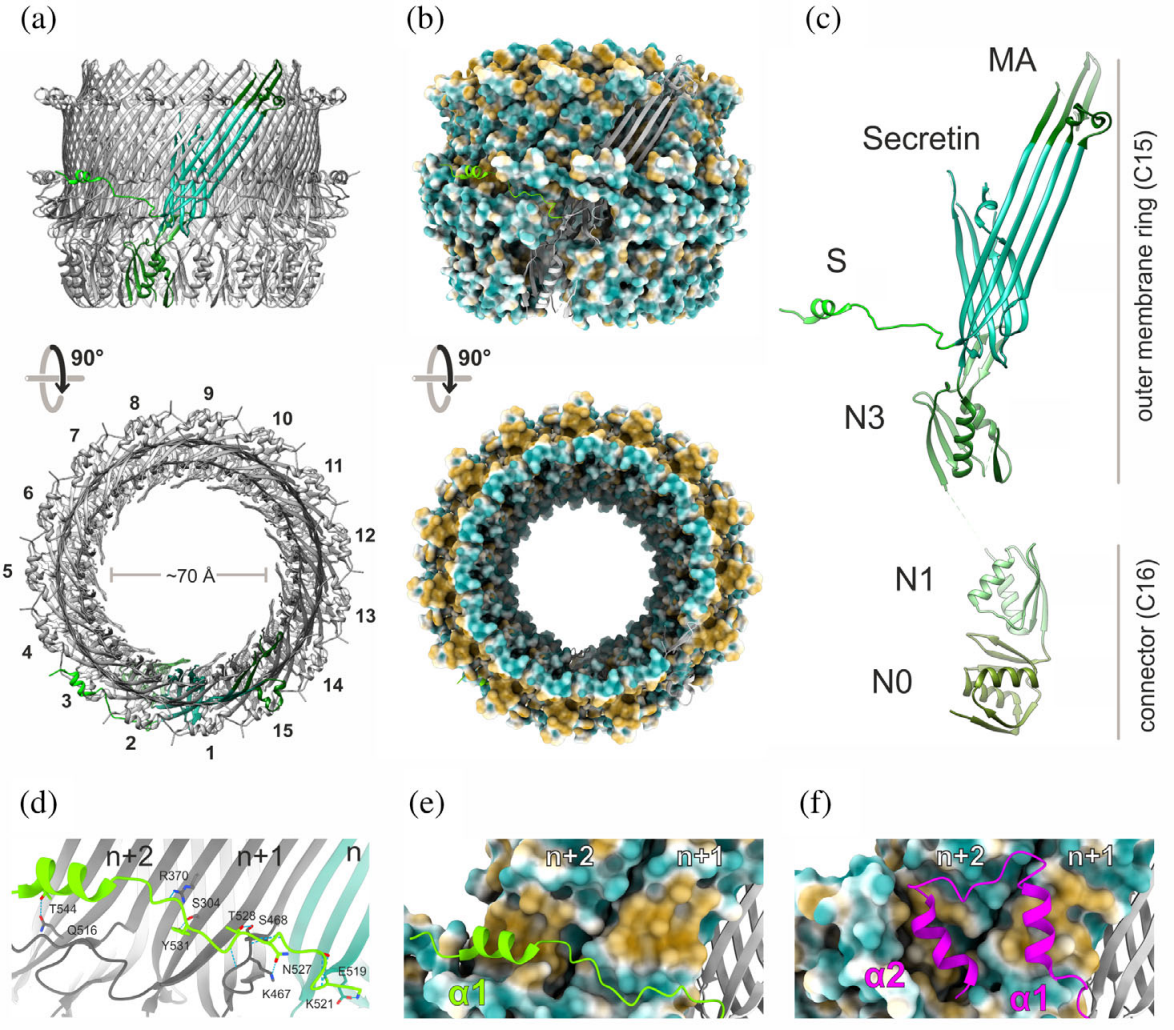
Structure of the secretin pore complex. (a) Side and top view of the outer membrane ring (MxiD[SctC]_180–548_) atomic model in cartoon representation with a single subunit highlighted in green and consecutively numbered subunits in the top view highlight pentadecameric (C15) assembly. (b) Side and top view of the outer membrane ring (MxiD[SctC]_180–548_) molecular lipophilicity potential (MLP) ranging from gold for hydrophobic, throughout white, to cyan for hydrophilic. (c) A single, MxiD[SctC]_34–548_ subunit with domains colored in different shades of green. While the domains N0 and N1 form the connecting region with a 16‐fold symmetry, the N3, Secretin, membrane‐associated (MA), and S domain assemble into the secretin pore complex with a 15‐fold symmetry. (d) Hydrogen bonds between the S domain (lime green) and the two following subunits (*n* + 1&2, gray). Dashed blue lines indicate hydrogen bonds, and involved side‐chain residues are displayed as sticks. Structure of the (e) *Shigella* and (f) *Salmonella* (PDB ID: 6DV6; Hu et al., 2018) S domains (as a cartoon) interacting with the two following secretin subunits (*n* + 1&2) displayed as molecular lipophilicity potential. While the first amphipathic helix (α1) interacts with the hydrophobic patch on the *n* + 2 subunit in *Shigella*, this position, in contrast, is occupied by the second helix (α2) in *Salmonella* SPI‐1.

Previously, we presented the structure of the *Shigella* OM ring (which is composed of the C‐terminal domains of MxiD[SctC]) built using the structure of the *Salmonella typhimurium* InvG[SctC] oligomer as a template (Lunelli et al., 2020). In the present study, the OM ring map obtained at 3.4 Å resolution allowed de novo atomic model building of the constituting domain (Figures 2, S3, 4b, and Table 1). The resulting structure includes the N3 (residues K180–H300) and the secretin (residues I301–I520) domains, and the residues K521–P548 of the previously unresolved S domain of MxiD[SctC] (Figure 2a–e).

Natively embedded in the outer membrane, the OM ring forms a pentadecameric (C15 symmetry) assembly (Figure 2a, b). The secretin domain of MxiD[SctC] forms a large double‐layered antiparallel β‐barrel with 60 strands in each wall (four in each of the 15 secretin subunits) (Figure 2a, c). While the individual walls are mainly stabilized by backbone hydrogen bonds of the β‐sheets, the interface between these two layers is predominantly hydrophobic and mediated by side chains. The inner wall forms a ring with a diameter of ~70 Å but (Figure 2a), due to unresolved density in two β‐turns on the distal side (Figure 2c), the actual inner diameter could be smaller. On the distal side, the outer four β‐strands of each monomer form a subdomain with a hydrophobic L‐shaped groove, which associates with the OM (Figure 2b, c). The proximal opening of the OM ring is lined by the N3 domain that contains the structurally conserved ring‐building motif (RBM) (Spreter et al., 2009) (Figure 2c), which promotes the secretin oligomerization. The hydrophobicity and the relatively large area (~1040 Å^2^) of the interface between adjacent N3 domains likely contribute to the ring stability. A β‐hairpin insertion in the RBM (A196–G209) extends against the inner wall of the secretin domain (Figure 2c). Overall, the above architecture is similar to the *Salmonella* pore complex (PDB ID: 6DV6; Hu et al., 2018), with RMSD of 1.2 Å for Cα atoms of the 290 aligned residues, revealing the structural conservation of the core of the secretin pore complex across species.

In contrast to this, the S domain in *Shigella* exhibits a unique conformation. While the *Salmonella* S domain adopts a helix‐turn‐helix conformation with the helices oriented perpendicular to the membrane (PDB ID: 6DV6, Figure 2f), the *Shigella* S domain helix extends parallel to the membrane plane (Figure 2d, e). It laterally enfolds in a long loop (K521–I535) mid‐height on the outer side of the secretin barrel and ends in a helix (S536 to Q545), thereby embracing the two following subunits. The loop interacts with the outer wall of the adjacent subunit mainly via hydrogen bonds (Figure 2d, and Table S2). The amphipathic helix interacts with the later subunit through a hydrophobic cluster on the outer wall surface (W306, I308, V368, L470, V512, and L514) and a single hydrogen bond (T544) (Figure 2d, e, and Table S2). The 18 amino acids following the helix, which might form a second helix and interact with the MxiM[SctG] pilotin (Okon et al., 2008), are not resolved in our EM map, suggesting the flexibility of this region. Overall, the S domain exhibits a relatively large interface area with the two following subunits (Table S3), creating an interaction network, in which every single MxiD[SctC] monomer is in contact with four adjacent monomers.

In *Salmonella*, the first helix (α1) interacts with the neighboring subunit (*n* + 1), and the second helix (α2) with the subunit after that (*n* + 2) (Figure 2f, and Table S3). Strikingly, the position *n* + 2, where the helix α2 interacts with the β‐barrel in *Salmonella*, is occupied by the helix α1 in *Shigella* (Figure 2e). In contrast to *Shigella*, the initial loop of the S domain is shorter in *Salmonella* (V519 to P524) and only three hydrogen bonds are formed with residues of the *n* + 1 subunit, while more hydrogen bonds and a salt bridge are formed with the *n* + 2 subunit (Table S4). The amino acids involved in these interactions are mostly conserved between the two bacterial species in the secretin domain, while they are not in the S domain (see alignment published in Lunelli et al., 2020). Thus, overall, *Shigella* and *Salmonella* adopt a similar architecture of the secretin pore complex but use unique conformations of their S domains to stabilize the β‐barrel.

### 3.1 The structure of the export apparatus reveals a conserved architecture and a previously unresolved SpaS[SctU] subunit

**FIGURE 3:**
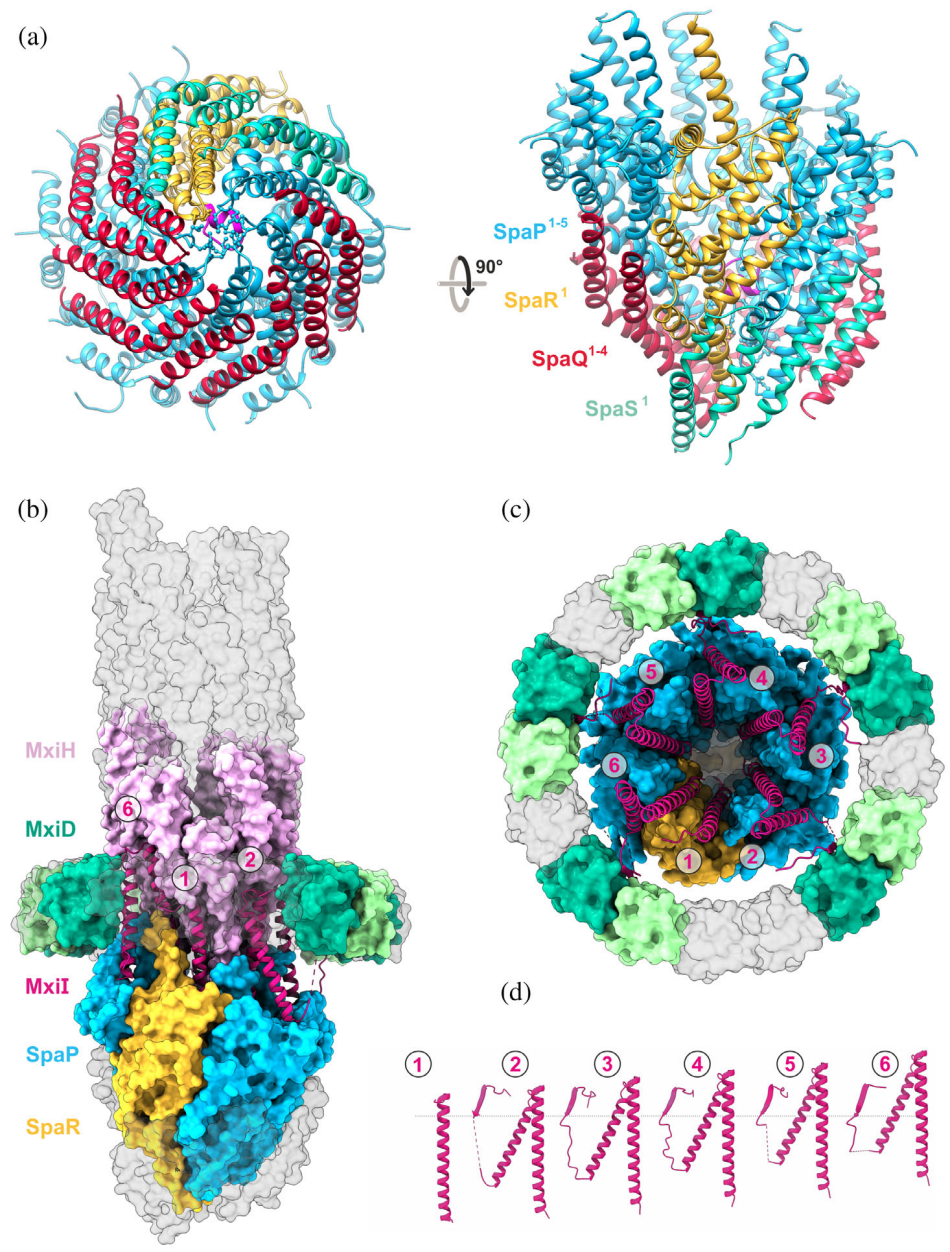
Structure of the export apparatus and the inner rod protein MxiI[SctI]. (a) Bottom (left) and side view (right) of export apparatus built in the C1 map as cartoon representation. The export apparatus is composed of five SpaP[SctR] subunits (blue), four SpaQ[SctS] (red), one SpaR[SctT] (yellow/magenta) and one SpaS[SctU] (turquoise, only the N‐terminal domain is observed in the cryo‐EM map). The side chains of the residues M178 and M179 of SpaP[SctR] and F213 of SpaR[SctT], which occlude the channel close to the proximal entry (P/R gasket), are represented as ball‐and‐sticks. The R‐plug, also occluding the channel immediately above the P/R gasket, is colored in magenta. (b) Side view of the needle complex core with subunits shown in surface representation, except for MxiI[SctF] (magenta), which is displayed as a cartoon. Protein subunits interfacing with MxiI[SctF] are opaquely colored and indicated on the right (SpaP[SctR]^1–5^ light blue; SpaR[SctT]^1^ dark yellow; MxiD[SctC]^1,2,4,5,7,8,10,11,13,14^ light and dark green; MxiH[SctF]^1–11^ pink), highlighting the connecting role of MxiI[SctF] within the needle complex. Proteins that are part of this structure but do not interact with MxiI[SctF] (SpaQ[SctS]^1–4^, SapS^1^, MxiD[SctC]^3,6,9,12,15,16^, MxiI[SctF]^12–28^) are displayed in transparent gray. One additional MxiD[SctC] subunit is left transparent to allow a view of MxiI[SctF]. (c) Top view of the needle complex core is colored as in (b) but with needle subunits (MxiH[SctF]) removed to allow a view on MxiI[SctF]. (d) Atomic model of MxiI[SctF]^1–6^ subunits as cartoon representation. The dotted line indicates the same level where the β‐sheet augmentation with MxiD[SctC] occurs, demonstrating how the height difference from the spiral arrangement of MxiI[SctF] is compensated by its loop region.

**FIGURE 4:**
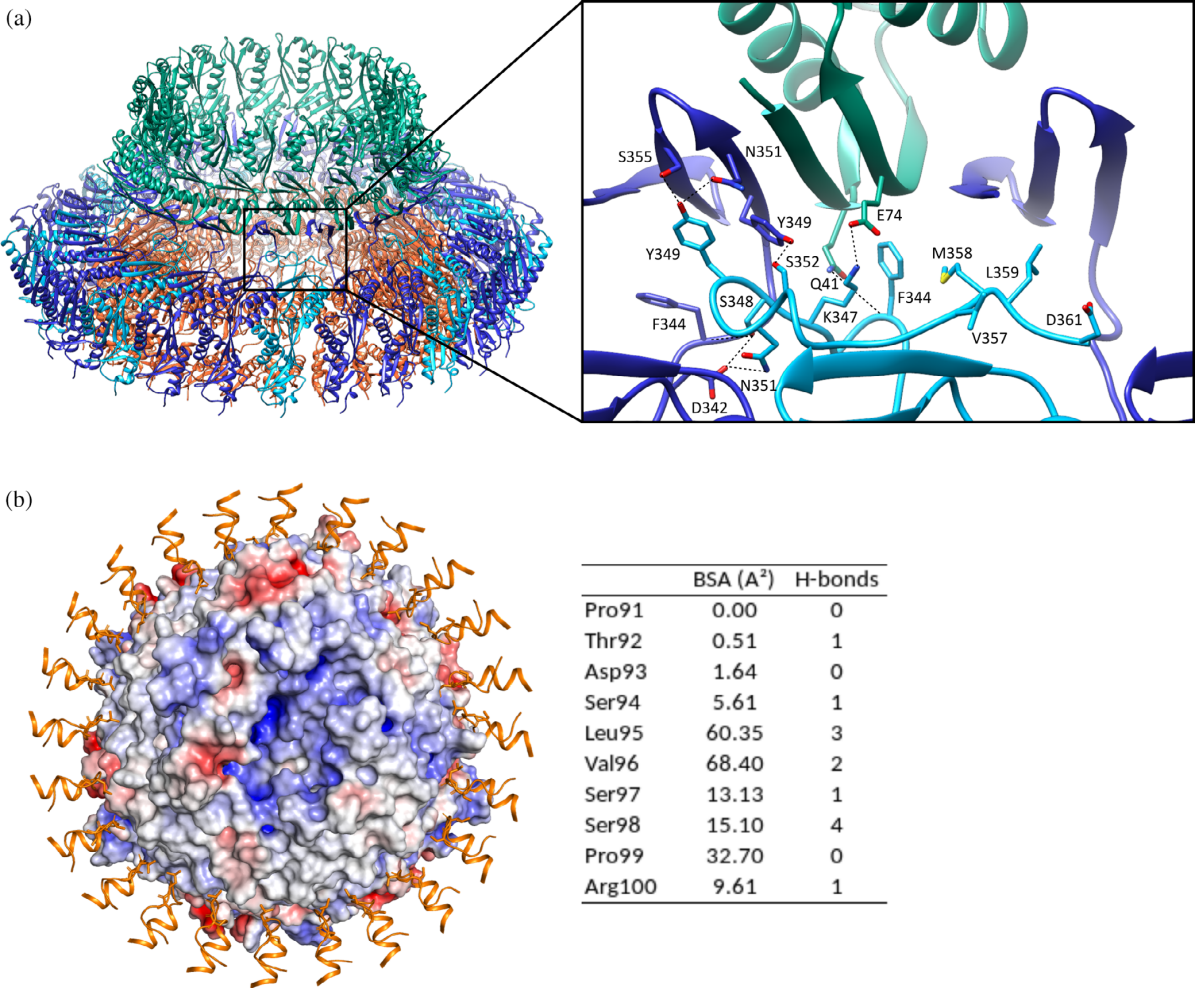
Inner membrane ring interactions with the connector and export apparatus. (a) C‐terminal domain of the MxiG[SctJ] subunits not bound to MxiD[SctC]. Cartoon representation of the IM ring (MxiG[SctJ] blue, MxiJ[SctJ] orange) and connector (N0 and N1 domains of MxiD[SctC] in green). The MxiG[SctJ] subunits not forming a β‐sheet with MxiD[SctC] are colored in light blue. The C‐terminal domain of one of these subunits is magnified on the right. Side chains of residues at the interface with their neighboring proteins or involved in hydrogen bonds are represented as sticks (backbone sticks are not shown for clarity). Intermolecular hydrogen bonds are shown as dashed lines (the hydrogen bond K345‐G346, which involves only the backbone of two MxiG[SctJ] subunits, is not shown for clarity). (b) Interaction of MxiJ[SctJ]_91–100_ with the export apparatus. Bottom view of the export apparatus surface is colored according to the electrostatic potential, surrounded by 24 copies of MxiJ[SctJ]_90–106_ shown as orange cartoons with the residues L95 and V96 depicted as sticks. The bottom end of the export apparatus misses several residues of SpaQ[SctS] and SpaS[SctU], not observed in the map, but not in contact with the MxiJ[SctJ] loop, thus the shown surface at the center of the bottom view does not reflect the real surface electrostatics. The table on the right shows the average buried surface area and the total number of hydrogen bonds formed between MxiJ[SctJ] and the export apparatus for the residue of the MxiJ[SctJ] loop.

The resolution of the C1 map allowed building the atomic model of the export apparatus (Figures 3a, S2, 4a, and Table 1). As in the previously published structures of the *Shigella* export apparatus (Johnson et al., 2019; Lunelli et al., 2020), our updated cryo‐EM structure includes five SpaP[SctR], four SpaQ[SctS], and one SpaR[SctT] subunit. In addition, we were able to model the N‐terminal domain of the SpaS[SctU] subunit (Figure 4a), which was not resolved in our previous reconstruction and also not resolved in the published structures of the *Salmonella* T3SS.

All subunits of the export apparatus are α‐helical proteins arranged in a helical fashion (Figure 4a). The SpaP[SctR] and SpaR[SctT] form the core of the complex, whereas the SpaQ[SctS] and SpaS[SctU] subunits form smaller helical hairpins that decorate the outer surface. The proximal side of the export apparatus is poorly resolved in the cryo‐EM map, allowing us to build only a partial model of three SpaQ[SctS] and SpaS[SctU] subunits. In particular, the conformation of the residues lining the proximal entry of the inner channel is not defined. The transport channel at this side is closed by the side chains of the residues F213 of SpaR[SctT] and M178, M179 of the five SpaP[SctR] subunits, while the flanking M177 and M180 do not extend their side chains in the channel lumen. Proceeding towards the distal opening, the residues 111–122 of SpaR[SctT] also occlude the channel (Figure 4a). These two structural features called, respectively, P/R‐gasket and R‐plug, are also present in the *Salmonella* export apparatus (Hu et al., 2019; Miletic et al., 2021). Compared with our *Shigella* structure, the methionine residues of the P/R‐gasket have a similar arrangement in the closed conformation of the *Salmonella* export apparatus (PDB IDs 6PEP; Hu et al., 2019 and 7AGX; Miletic et al., 2021). The residues of the R‐plug, however, show some deviation in the 7AGX *Salmonella* structure, and they are not modeled in the earlier 6PEP structure. This indicates high flexibility of the R‐plug loop, consistently with its role as a barrier that should open when the substrate enters the channel.

The overall architecture of the *Shigella* export apparatus is well conserved in the structures from different species solved so far, either from flagellar (Kuhlen et al., 2018, 2020) or virulence‐associated T3SS (Hu et al., 2019; Kuhlen et al., 2020; Miletic et al., 2021), likely owing to its central role in secretion. However, in *Salmonella*, SpaS[SctU] is not observed, probably lost during purification, also causing the most proximal SpaQ[SctS] subunit to adopt a more open conformation (Miletic et al., 2021). In the flagellar export apparatus of *Pseudomonas savastanoi*, a fifth FliQ[SctS] subunit, a homolog of SpaQ[SctS], occupies the position of SpaS[SctU]. The N‐terminal domain of FlhB[SctU], also a homolog of SpaS[SctU], has been observed only in the flagellar export apparatus of *Vibrio mimicus*. It is composed of two helical hairpins connected by a long loop, which wraps around the proximal opening (Kuhlen et al., 2020). Although we do not observe the long loop, the N‐terminal domain of SpaS[SctU] has a similar topology to the flagellar FlhB[SctU]. The first helical hairpin occupies the position of the fifth SpaQ[SctS] subunit in our previous model of the export apparatus (Lunelli et al., 2020), but with inverted orientation along the long axis, that with the termini pointing towards the proximal end. The second hairpin occupies the position of a sixth imaginary SpaQ[SctS] subunit along the pseudohelical arrangement of the export apparatus components, again inverted compared with the orientation of the SpaQ[SctS] subunits. The SpaS[SctU] transmembrane region and the C‐terminal domain are not defined in our map.

### 3.2 Inner rod protein, MxiI[SctI], provides flexible interface connecting export apparatus and needle

Our previous model (Lunelli et al., 2020) of the needle subunits adjacent to the export apparatus included only polyalanine peptides, thus concealing which of MxiI[SctI] or MxiH[SctF] was the true identity of the initial few turns. The improved C1 map presented here clearly shows that six MxiI[SctI] subunits assemble into a single helical turn before being continued by the needle protein MxiH[SctF] (Figures 3b, c, S8a). The six MxiI[SctI] subunits build the first turn of an upward spiral on the distal side of the export apparatus creating interfaces with all SpaP[SctR] (SpaP[SctR]^1–5^) and the only SpaR[SctT] subunit of the complex (Figure 3b, c). The lowest (MxiI[SctI]^1^) and the highest MxiI[SctI] (MxiI[SctI]^6^) interface each other above the SpaR[SctT] subunit.

Although in the lowest MxiI[SctI] (MxiI[SctI]^1^), we could not model the residues beyond the first helix, the other five remaining MxiI[SctI] subunits (MxiI[SctI]^2–6^) displayed a very similar structure to each other (Figure 3d). Starting from the C‐terminus, they adopt a helix‐turn‐helix conformation followed by a partially unresolved long loop region, which includes a short β‐strand located at the interface between two connector subunits. The β‐strand augments a β‐sheet of the N1 domain of MxiD[SctC] (S112‐V116) (Figure S8b), anchoring the needle‐ and export apparatus structure within the basal body. At the same time, the N‐terminal segment of the loop extends along the upper rim of the connector. The loop compensates for the height difference of the single MxiI[SctI] subunits caused by their helical arrangement (Figure 3d). In this way, the different MxiI[SctI] subunits can interact with MxiD[SctC] at the same height even though they are located at varying levels.

After the first turn of MxiI[SctI], the upward helical structure is continued by the needle protein MxiH[SctF] (Figures 3b, S9a). MxiH[SctF] adopts a similar helix‐turn‐helix conformation as previously mentioned for MxiI[SctI] (Figure S9). While in the subunits of the first turn (MxiH[SctF]^1–5^) half of the N‐terminal helix is not resolved in the map, starting from the second turn (MxiH[SctF]^6–28^) the protein is more ordered, thus revealing a kink within the N‐terminal helix (Figure S9b). Taken together, each MxiI[SctI] (MxiI[SctI]^2–6^) subunit interfaces with two export apparatus proteins (SpaP[SctR]/SpaR[SctT]), two connector proteins (MxiD[SctC]), and three needle proteins (MxiH[SctF]), demonstrating its role in mediating interactions among the subcomplexes of the needle complex.

A similar stoichiometry of the inner rod protein was also found in *Salmonella* SPI‐1 T3SS (Hu et al., 2019; Miletic et al., 2021; Torres‐Vargas et al., 2019; Zilkenat et al., 2016). While we could not fully model the lowest MxiI[SctI]^1^, the *Salmonella* orthologue PrgJ[SctI]^1^ has been shown to adopt a unique fold compared to the other PrgJ[SctI]^2–5^ subunits. Thereby its N‐terminus first bridges across SpaR[SctT] and SpaP[SctR], before its β‐strand eventually complements with the MxiD[SctC] orthologue InvG[SctC] (Miletic et al., 2021). In our structure, however, based on the similarity with the other MxiI[SctI] subunits, we tentatively assigned that β‐strand in the mentioned position to the MxiI[SctI] subunit thereafter (MxiI[SctI]^2^). This location has also been previously proposed as one of the two alternate conformations of PrgJ[SctI]^2^ in *Salmonella*, possibly caused by the insertion of an additional InvG[SctC] subunit in the connector (Hu et al., 2019).

### 3.3 Inner membrane ring interactions with connector and export apparatus

In our previous study (Lunelli et al., 2020), we found the MxiG[SctD] ring is composed of eight triplets of subunits differing in the conformation of the C‐terminal domain. Two subunits from each triplet form a three‐stranded β‐sheet that joins the β‐sheet of the N0 domain of two neighboring MxiD[SctC] subunits to form a continuous circular sheet at the basis of the secretin oligomer. The higher resolution of our new map focused on the IM ring and connector with imposed C8 symmetry (Table 1), confirms this finding. Moreover, it allowed us to build the C‐terminal domain of the third MxiG[SctD] subunit up to residue D361 (Figure S4c). It forms a long loop, which occupies the region of space between IM ring and the circular β‐sheet delimited by the two other MxiG[SctD] subunits (Figures 4a, S10). It is mostly hydrophilic: eight residues are charged, seven polar, and six hydrophobic. The relatively high B‐factor and the weak density in this region indicate its higher flexibility. Nevertheless, the C‐terminal domain forms a relatively large interface (~460 Å^2^) with the adjacent MxiG[SctD] subunit within which the segment K345‐S352 forms several hydrogen bonds. On the contrary, no hydrogen bonds are formed with the other MxiG[SctD] neighbor, and only a few mostly hydrophobic residues interface with it (F344, V357, M358, L359, and D361). It is likely, however, that the last 10 MxiG[SctD] residues which are not resolved in the map, are also in close contact with the same MxiG[SctD] neighbor. The interface between MxiG[SctD] and MxiD[SctC] is also small (~165 Å^2^) and includes one salt bridge (K347–E74) and one hydrogen bond (F344–Q41). Notably, the C‐terminal domain of the *Salmonella* PrgH[SctD], an orthologue of MxiG[SctD], shows a conformation less extended in the interspace between IM ring and connector, forming a small β‐hairpin, which is missing in *Shigella*, oriented vertically and reaching one of the helices of the secretin N0 domain (Hu et al., 2019). This difference, compared with the well‐conserved conformation of the surrounding domains, suggests either a different role among species or dispensability of this domain.

The MxiJ[SctJ] subunits of the IM ring contact the export apparatus through two loops (P91–R100 and Y133–P144). Since each one of the 24 MxiJ[SctJ] copies must adapt to different patches of the export apparatus surface these two loops are expected to be conformational flexible. Consequently, imposing symmetry in this region averages out the conformational variability of these loops, preventing detailed analysis of the interface. However, the C1 map, although at a relatively low resolution (Figure S2), allows tentative building of the 24 copies of the lower loop (P91–R100). It interacts with the SpaQ[SctS] and SpaS[SctU] subunits and includes at its tip the conserved hydrophobic residues L95 and V96 (Lunelli et al., 2020) (Figure 4b). The average surface area of these residues buried by the contact with the export apparatus is 60.4 and 68.4 Å^2^, respectively, considerably higher than the average area buried in the other more hydrophilic residues of the loop (Figure 4b). Altogether, the 24 copies of the loop form 13 hydrogen bonds with the components of the export apparatus, five of them involving the conserved residues S97 and S98 and five the backbone of L95 and V96. The SpaQ[SctS]/SpaS[SctU] surface of the export apparatus is predominantly hydrophobic, with some hydrophilic patches (Figure 4b). The hydrophobic tip of this loop provided by the residues L95 and V96, supported by the polar bonds of the peptide backbone and the neighboring S97 and S98, allows this loop to adapt to the different residues on the complementary export apparatus surface.

### 3.4 The pilotin, MxiM[SctG], remains associated with the needle complex secretin ring

**FIGURE 5:**
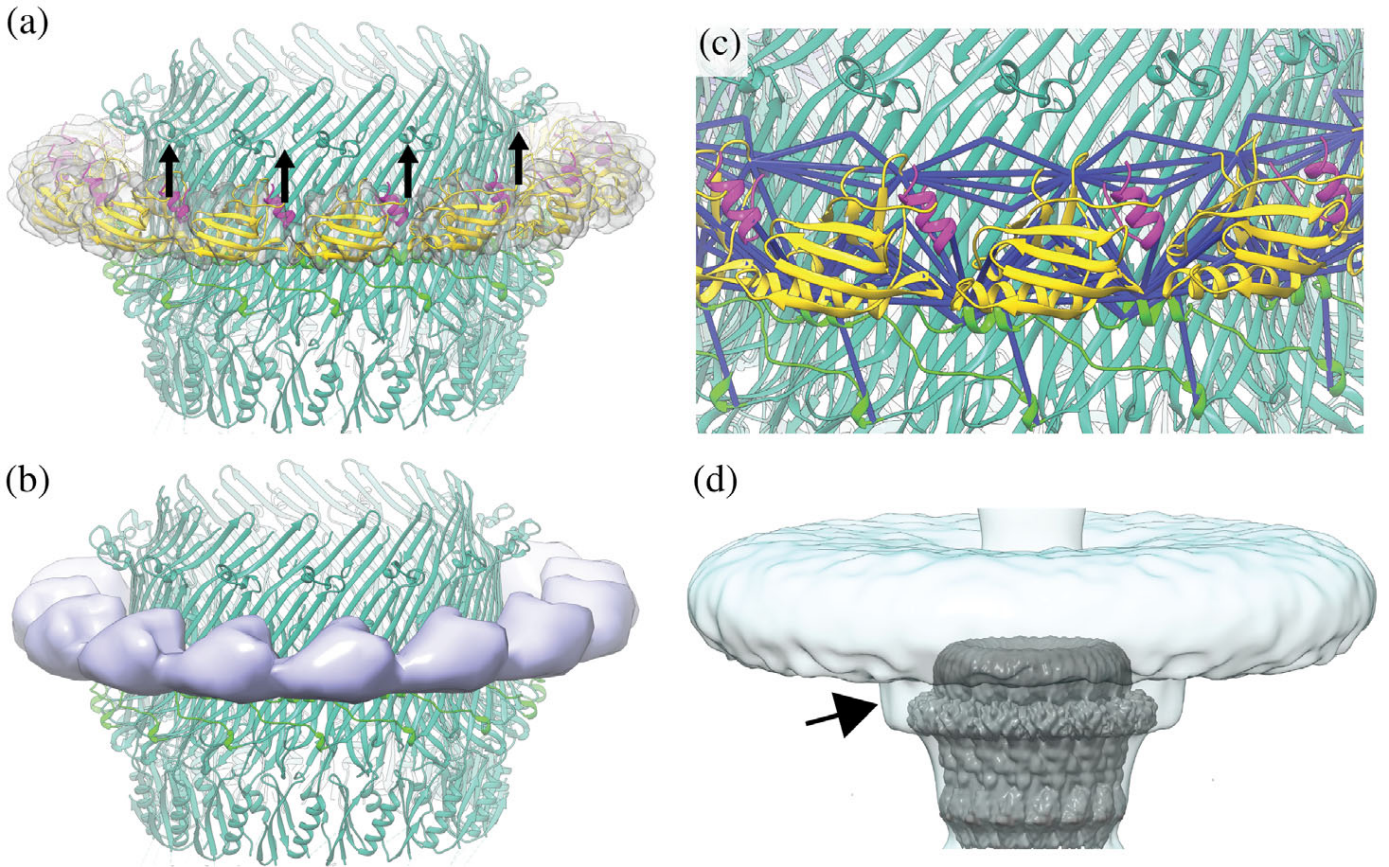
Structure of the secretin‐pilotin complex. (a) A model of the secretin‐pilotin complex (cluster 2 in Figures S13, S14) including MxiD[SctC]_180–548_ from cryo‐EM as cartoon representation (green) with additional surrounding density (transparent gray) from our study and MxiM[SctG] (yellow) in complex with MxiD[SctC]_549–566_ (purple, PDB ID 2JW1; Okon et al., 2008) fitted into the EM density ring. Arrows indicate the orientation of the lipidated MxiM[SctG] N‐terminus. (b) The localization probability density (violet solid surface) represents the ensemble of the MxiM[SctG] conformations in cluster 2. (c) Cross‐links satisfying the distance threshold of 30 Å (dark blue) between MxiD[SctC]_180–548_ and MxiM[SctG] in complex with MxiD[SctC]_549–566_ (cluster 2). Violated cross‐links to more distant regions of MxiD[SctC] are not shown. (d) Superposition of the MxiD[SctC] cryo‐EM density from this study (gray solid surface) with the cryo‐ET density (cyan, transparent, volume contorting threshold of 0.726) from *Shigella* T3SS in mini‐cells (Hu et al., 2015) indicates similar putative MxiM[SctG] location (arrow).

Many secretins depend on their conjugate pilotins for localization and/or stabilization, which also applies to MxiD[SctC] and MxiM[SctG] of *Shigella* (Schuch & Maurelli, 1999, 2001). Furthermore, MxiM[SctG] binds MxiD[SctC], and its structure had been solved in complex with the C‐terminal MxiD[SctC] residues (S549–Y566), which form a loop and a second S domain helix (α2, residues E557–N565) (Okon et al., 2008). Interestingly, these residues are not resolved in our MxiD[SctC] model, which ends at residue P548. Analysis of the cross‐linking MS data indicated in total 26 cross‐links formed between MxiD[SctC] and MxiM[SctG]. Twelve of these cross‐links were concentrated in the region of the MxiD[SctC] S domain helix (α1) (Figures 1c, S11). The same region forms intra‐MxiD[SctC] cross‐links to the last C‐terminal MxiD[SctC] helix α2 (Figure S11). This region is adjacent to a weak low‐resolution cloud of density surrounding the secretin pore on the periplasmic side of the OM (Figure 5a). We thus hypothesized that this density, which remained unassigned in our previous study, reflects the presence of multiple copies of MxiM[SctG], forming a ring around the secretin oligomer surface.

To test this hypothesis, we docked the structure of MxiM[SctG] (Okon et al., 2008), solved in complex with the C‐terminal MxiD[SctC] residues (S549–Y566), to our cryo‐EM model of the OM ring, using the integrative modeling platform (IMP) (Webb et al., 2018) and Assembline software (Rantos et al., 2021).

Using both cross‐links, the EM density map with C15 symmetry as restraints for integrative modeling (Figure S12), and assuming 15 copies of MxiM[SctG], we found three different clusters of orientations of MxiM[SctG], each satisfying an overlapping but different subset comprising ~65% of all secretin‐pilotin cross‐links (Figures S13, S14). These three pilotin binding modes are characterized by the main β‐sheet adopting a skewed (cluster 0), a parallel (cluster 1), or a perpendicular orientation (cluster 2) relative to the cylindrical axis of the secretin ring. In all three orientations, a rearrangement of the loops E545–P548 and S549–D556 could ensure the connectivity of the MxiD[SctC] residues in the two structures. In the perpendicular orientation, however, MxiM[SctG] showed the best agreement with the cryo‐EM map (Figure 5a–c). Additionally, in this arrangement, the lipidated N‐terminus of MxiM[SctG] orients toward the outer membrane, suggesting that this orientation might represent the most stable pilotin binding mode under physiological conditions. The cross‐linking positions of MxiD[SctC] to MxiM[SctG] were distributed over a wide area of the MxiD[SctC] ring outer surface (Figure S11), with some being not compatible with MxiM[SctG] located in the EM density map. Thus, we also performed modeling using cross‐linking‐MS data alone obtaining five clusters (Figure S15) that satisfied further cross‐links leaving seven cross‐links violating the 30 Å distance (Figure S16). Four of the remaining cross‐links are only slightly violated and still support the binding of MxiM around the S domain while the three remaining cross‐links would have to be explained by false positive identifications, large compression of the needle complexes, or aggregation. Altogether, the modeling based on cross‐links only suggests that, in a purified needle complex, MxiM[SctG] can explore a broader range of transient locations around the secretin ring, probably not stable or populated enough to appear in the EM map.

The superposition of our cryo‐EM map with the in situ cryo‐electron tomography (cryo‐ET) map of the *Shigella* T3SS (Hu et al., 2015) indicates that the MxiM[SctG] position modeled based on the cryo‐EM density exactly coincides with a yet unassigned density “belt” observed in membrane‐embedded complexes (Figures 5d, S17a). No such density could be observed in the cryo‐ET map of *Salmonella* (Figure S17b, c), suggesting a different supramolecular organization of the T3SS OM ring compared with *Shigella*. Altogether, the docking results indicate that MxiD[SctC] is surrounded by multiple MxiM[SctG] subunits, forming a ring localized at the level of the S domain.

## 4 DISCUSSION

In this study, we obtained cryo‐EM structures of the *Shigella* needle complex at a resolution that allows building an atomic model of almost the entire system comprising 10 different proteins, some present in multiple copies and partly adopting different conformations including in total 101 individual polypeptide chains (MxiH[SctF] needle subunits excluded). The combination of cross‐linking MS, cryo‐EM and integrative modeling revealed the presence of the pilotin (MxiM[SctG]) oligomer forming a ring encompassing the secretin that would have remained undefined when investigating solely by cryo‐EM.

The export apparatus shares the pseudohelical arrangement with the previously described homologous structures (Hu et al., 2019; Johnson et al., 2019; Kuhlen et al., 2018, 2020; Miletic et al., 2021). Like in *Salmonella*, it is anchored to the connector within the needle complex by the inner rod protein MxiI[SctI] (Hu et al., 2019; Miletic et al., 2021). While the helical hairpin of the MxiI[SctI] subunits creates an interface with the proximal needle and with the SpaP/R[SctR/T] core of the export apparatus, the N‐termini anchor it to the connector by a β‐sheet augmentation with MxiD[SctC]. Additionally, the export apparatus is in contact with the IM ring via two flexible loops of MxiJ[SctJ]. Our structure reveals that these features are similar in both *Shigella* and *Salmonella*, and likely represent conserved features of T3SSs. Similarly, the low stoichiometry of MxiI[SctI] arranged as a single helical turn was predicted for the *Salmonella* homologue PrgJ[SctI] based a on proteomic approach (Zilkenat et al., 2016) and *in vivo* photo cross‐linking (Torres‐Vargas et al., 2019), before it was confirmed from structural data (Hu et al., 2019).

Apart from the previously mentioned similarities, however, we discovered marked differences in our *Shigella* structure as well. This includes the N‐terminal domain of SpaS[SctU] observed on the proximal side of the export apparatus. In previous studies of the *Salmonella* needle complex, it was assumed to be lost during purification (Hu et al., 2019; Miletic et al., 2021) and it remained undescribed for any virulence‐associated T3SS. It is unclear whether its presence after complex isolation is due to physiological differences or whether it is a result of a varying purification procedure. Also different between the two pathogens are the conformations of the C‐terminal domains of the MxiG[SctD] subunits, which are not binding to the connector by β‐sheet augmentation. While two subunits in the MxiG[SctD] triplets cohere the IM ring with the connector, the third copy locates its last domain in the interspace between IM ring and connector. This third conformation varies between *Shigella* and *Salmonella* (Hu et al., 2019) and further investigation is necessary to determine if this has a functional relevance.

The most striking difference appears to be the conformation of the S domain of the secretin and the attached pilot protein MxiM[SctG]. While the *Salmonella* S domain adopts helix‐turn‐helix conformation with the helices oriented perpendicularly to the membrane (PDB ID: 6DV6; Hu et al., 2018) the *Shigella* S domain adopts a more extended structure with a helix oriented horizontally. In both species, the amphipathic S domain helices interact with a conserved hydrophobic patch on the outer wall surface (Figure 2e, f). In *Salmonella*, the first helix α1 interacts with a patch of the directly neighboring subunit *n* + 1, and the second helix α2 with the following subunit *n* + 2. In our *Shigella* structure, however, the first helix α1 interacts with the second neighboring *n* + 2 subunit directly, seemingly compensating for the missing second helix as found in *Salmonella*. Thus, despite different structures, both species bind the S domain at the same surface region of neighboring subunit. This region might be then critical for stabilizing the secretin ring across species and makes an interesting surface patch for targeting with therapeutics.

The *Salmonella* model only lacks the last five C‐terminal residues of the S domain while ours lacks the last 18, indicating disorder or flexibility of this region. Okon et al. (2008) could show that these 18 residues interact with the pilotin, MxiM[SctG], and rearrange into a loop‐helix conformation upon contact. Interestingly, our MS data reveals the presence of this pilotin, in the isolated needle complex. Multiple cross‐links between MxiM[SctG] and MxiD[SctC] prove that MxiM[SctG] remains bound to the T3SS needle complex upon purification (Figure 1c), consistently with earlier experiments (Sani et al., 2007; Zenk et al., 2007).

Closer examination of the cross‐links reveals that the MxiM[SctG] cross‐links are widely distributed throughout the secretin pore complex (Figure S11) indicating multiple possible poses of MxiM[SctG]. Combining cross‐linking MS and EM data in an integrative modeling approach, allowed us to obtain three different orientations of MxiM[SctG] in relation to MxiD[SctC], each satisfying ~65% of secretin‐pilotin cross‐links (Figures S13, S14). We speculate that the pilotin MxiM[SctG] is in complex with the last α2 helix of the S domain and that this complex is flexible, attributed to the loss of membrane, in which the lipidated N‐terminus of MxiM[SctG] could anchor. This dynamic heterogeneity, as seen by the cross‐links, would explain why we only observe a weak featureless density in the EM maps for MxiM[SctG] as well as for the last 18 residues of MxiD[SctC]. Additionally, this assumption is supported by the intra‐MxiD[SctC] cross‐links (Figure 1c)—cross‐links from the last C‐terminal residues (K558 and S549) of MxiD[SctC] (which are lacking in our EM structure, and are in complex with the pilotin) are found with almost the same residues of the secretin pore ring as the cross‐links from the pilotin (Figure S11). Thus, MxiM[SctG] and last MxiD[SctC] residues locat in the same regions, are probably bound together. If the observed flexibility of MxiM[SctG] is caused by the extraction from the membrane, then the biologically relevant structure is likely to have the lipidated N‐terminus oriented towards the OM as in our model (cluster 2) (Figure 5a–c). Similar orientation with the N‐terminal tail directed toward the OM has been found in the T2SS pilotin AspS (Yin et al., 2018). Even though further experiments would be needed to prove the biologically relevant orientation of MxiM[SctG], our integrative modeling approach (Figures 5, S13) confirms previous suggestions of the pilotin localization. Based on the function of the pilotin (Schuch & Maurelli, 1999) and the resemblance to the PulDPulS complex (Nouwen et al., 1999), Sani et al. (2007) proposed that density spikes found around the OM ring of the isolated *Shigella* needle complexes might originate from the pilotin. Similarly, cryo‐ET of *Shigella* minicells displayed a density around the secretin pore on the periplasmic side of the OM (Hu et al., 2015), which has been hypothesized to be the pilotin.

These studies suggest that the pilotin remains associated with the *Shigella* needle complex beyond secretin assembly. Interestingly, this does not seem to be the case for *Salmonella*, as no additional density around the secretin, comparable to ours, is observable in published cryo‐EM maps of *Salmonella* needle complex (Hu et al., 2018; Miletic et al., 2021). Furthermore, the density below the OM, visible in cryo‐ET from *Shigella* minicells (Hu et al., 2015; Tachiyama et al., 2019) in which the pilotin is proposed, is not visible in *Salmonella* minicells (Hu et al., 2017) (Figures 5d, S17). The seemingly stark difference in pilotin abundance between *Shigella* and *Salmonella* leaves room for speculation. It might reflect distinct conformations during different stages of infection. Furthermore, pilotins might exhibit additional roles within the needle complex beyond the initial localization and oligomerization of the secretin. This could include structural stabilization, sensing, or even regulation of some sort that may differ to meet the individual pathogen requirements. However, more research will be necessary to decipher the molecular mechanisms of the diverse pilotins within the T3SSs.

Taking together, this study leads to a better understanding of the *Shigella* T3SS and, by revealing conserved and unique futures, highlights the need to analyze the T3SS in a species‐specific manner.

## 5 MATERIALS AND METHODS

### 5.1 Bacterial strains

The *S. flexneri* mutant M90T Δ*ipaD* Δ*mxiH* complemented with an N‐terminal Strep‐tagged MxiH[SctF] was used as described by Lunelli et al. (2020).

### 5.2 Cryo‐electron microscopy

The data set processed in the study was collected with the microscope Titan Krios (Thermo Fisher Scientific, Waltham, MA) equipped with a Falcon II detector. Sample vitrification and data collection are described in our previous study (Lunelli et al., 2020).

We used Relion 3.0 (Scheres, 2012a, 2012b) to process the data. Each micrograph is composed of 7 frames. We removed the 7th frame of each micrograph, which is the most exposed and therefore the most subjected to damages from the electron beam and aligned the remaining 6 frames with Relion. Defocus values for the CTF correction were determined with CTFFIND 4.1.13 (Rohou & Grigorieff, 2015). We removed micrographs with estimated resolution worse than 7 Å and figures of merit lower than 0.05. The particles autopicked in our previous study (Lunelli et al., 2020) were re‐extracted from the micrographs. The best classes obtained by two rounds of 2D classification with 200 and 100 classes included 105,927 particles (Figure S18a), which were subjected to 3D classification with three classes using as reference our previous reconstruction of the needle complex low‐pass filtered at 40 Å. We did not impose any symmetry and used a soft solvent mask that included the entire needle complex. We increased the angular sampling from 3.7° to 0.9° (parameter healpix_order from 3 to 5) in three steps when the distribution of particles among the classes and their resolution was stable. A class included 90,547 particles (~85% of the input particles) that were used for an initial 3D refinement, which resulted in a reconstruction at 7 Å resolution. After Bayesian polishing of the particles, another 3D refinement improved the resolution to 6.5 Å, and after CTF refinement (per‐particle defocus) a final refinement using solvent‐flattened FSC delivered a reconstruction at 4.05 Å resolution. The post‐processed map (at the same resolution) was sharpened with b‐factor − 103.

The same set of CTF‐refined particles was used for the focused reconstructions of the IM ring with C24 symmetry, and of the IM ring and connector with C8 symmetry. We created the references and masks for the focused reconstructions with Segger 1.9.5 (Pintilie & Chiu, 2012) into Chimera 1.14 (Pettersen et al., 2004) from the map of the full needle complex. However, we could not obtain a satisfactory result from a simply focused reconstruction of the OM ring. Thus, we employed the partial signal subtraction technique (Bai et al., 2015) to enhance the signal of the OM ring region (Figure S18b). The subtracted particles were further classified into three classes imposing C15 symmetry. A class included ~76% of the particles, which were used for the focused reconstruction of the OM ring with C15 symmetry and solvent‐flattened FSC. The resulting map at 3.44 Å resolution was post‐refined and sharpened with b‐factor − 108.

### 5.3 Model building and refinements

We built the atomic structure of the inner components of the needle complex (Figure S4a) either starting from our previously published structure (PDB ID 6RWY for SpaP[SctR], SpaQ[SctS], SpaR[SctT]) or from a homology model based on a similar structure of the *Salmonella* needle complex (PDB ID 6PEP; Hu et al., 2019) for MxiH[SctF] and MxiI[SctI] using sequence alignments as published previously by Lunelli et al. (2020) We used Coot 0.8.9.3 (Emsley et al., 2010) to build the structures of SpaP[SctR], SpaQ[SctS], SpaR[SctT], MxiI[SctI] and MxiH[SctF] into our unsymmetrized map of the *Shigella* needle complex. We extended the needle inside the basal body. We also found unoccupied density at the proximal side of the export apparatus, which appeared similar to the density of the FlhB subunit in the structure of flagellar export apparatus of *Vibrio mimicus* (PDB ID 6S3L; Kuhlen et al., 2020). Using this model as a guide, we built and refined four helices of SpaS[SctU] into the unoccupied density. Finally, we included in our model 16 copies of the N1 domain of MxiD[SctC], which interacts with the N‐terminus of MxiI[SctI], and 24 copies of the MxiJ[SctJ] helix‐loop 90–106, which interacts with the export apparatus subunits. The final model includes the following polypeptides: SpaP[SctR]_1–214_ (unobserved 78–93, 121–130), SpaP[SctR]_1–214_ (unobserved 74–93, 122–132), SpaP[SctR]_1–214_ (unobserved 73–92, 122–127), SpaP[SctR]_1–214_ (unobserved 72–93, 122–130), SpaP[SctR]_1–215_ (unobserved 73–92, 121–129), SpaR[SctT], SpaQ[SctS]_1–85_, SpaQ[SctS]_1–85_ (unobserved 37–46), SpaQ[SctS]_1–85_ (unobserved 26–47), SpaQ[SctS]_1–85_ (unobserved 29–46), SpaS[SctU]_30–188_ (unobserved 84–129), MxiI[SctI]_56–97_, MxiI[SctI]_5–97_ (unobserved 15–22), MxiI[SctI]_3–97_, MxiI[SctI]_5–97_, MxiI[SctI]_5–97_ (unobserved 17–24), MxiI[SctI]_8–97_ (unobserved 20–24), 2 copies of MxiH[SctF]_24–83_, 3 copies of MxiH[SctF]_25–83_, 3 copies of MxiH[SctF]_8–83_, 3 copies of MxiH[SctF]_9–83_, 10 copies of MxiH[SctF]_2–83_, 6 copies of MxiH[SctF]_3–83_, MxiH[SctF]_6–83_, 16 copies of MxiD[SctC]_110–171_, 24 copies of MxiJ[SctJ]_90–106_.

The secretin OM ring was built into the C15 OM ring map with Coot (Figures S3, S4), starting from a homology model based on the *Salmonella* InvG[SctC] pore (PDB ID 6DV6; Hu et al., 2018) including the N3 and secretin domains, already discussed in our previous paper (Lunelli et al., 2020), while the S domain was built *de‐novo*. The model includes MxiD[SctC]_180–548_ (unobserved 231–260, 342–349, 396–400, 437–442).

The model of IM ring and connector was fit in the C8 map with Chimera fitmap and built with Coot (Figure S4c) starting from our models of these regions (PDB ID 6RWX and 6RWK; Lunelli et al., 2020). The C‐terminal domain of 16 MxiG[SctD] subunits in the connector model 6RWK was joined with the end of the corresponding subunits in the 6RWX structure (residue 340). Residues 341–361 of the remaining 8 MxiG[SctD] subunits, which were missing in our previous models, were built *de‐novo*. The model includes 16 copies of MxiG[SctD]_151–368_, 8 copies of MxiG[SctD]_151–361_, 24 copies of MxiJ[SctJ]_20–199_, 16 copies of MxiD[SctC]_34–171_.

All the models were refined with phenix.real_space_refine of the phenix package version 1.18.2–3874 (global minimization + group B‐factors refinement) (Liebschner et al., 2019). We applied NCS constraints, corresponding to the symmetry of the map used to build the model, and also secondary structure restraints. The quality and resolution of the models were assessed with phenix.molprobity, phenix.mtriage and pheni.emringer.

### 5.4 Cross‐linking mass spectrometry

The T3SS needle complexes were isolated as in Lunelli et al. (2020) with minor adaptations to enable downstream cross‐linking procedure. Here, bacteria were grown in LB medium (Luria/Miller) and harvested at OD_600_ ~ 0.25. After the osmotic shock and spheroplast formation, cell lysate was centrifuged at 100,000 x *g* overnight. The resulting pellet was solubilized in 50 mM HEPES, 100 mM NaCl, 5 mM EDTA, 0.04% Triton X‐100 pH 8.00 before being subjected to Strep‐tag affinity purification. Eluted fractions were flash‐frozen in liquid nitrogen and stored at −80°C until further use. Small aliquots of each fraction were retained and applied to glow‐discharged, carbon‐coated copper grids (electron microscopy sciences) and negatively stained with 1% uranyl acetate. Grids were checked for needle complexes with a Talos L120C transmission electron microscope (ThermoFisher Scientific, Inc.), and decent fractions (e.g., Figure S5a) were pooled to ensure consistent quality for the cross‐linking procedure.

To determine optimal protein‐to‐cross‐link‐ratio, isolated T3SS needle complexes were incubated with increasing concentrations (0.05–2 mM) of Bis(sulfosuccinimidyl)suberate (BS^3^) (ThermoFisher Scientific, Inc.) at room temperature and quenched after 30 min. Subsequently, cross‐linked samples were run on Mini‐Protean TGX SDS‐PAGE (Bio‐Rad Laboratories, Inc.) and either silver stained (Figure S5c) or western blotted (Figure S5d) for evaluation. For western blotting, samples were transferred to a polyviylidene difluoride membrane (GE Healthcare Life Sciences) and incubated with anti‐MxiG[SctD] (Max‐Planck Institute for Infection Biology, Protein Purification facility, Berlin) followed by antimouse (Jackson ImmunoResearch Europe Ltd.).

The selected ratio at 0.2 mM BS3 (marked with an asterisk, Figure S5c, d) was chosen for final sample preparation. Whether aggregation occurred due to cross‐linking was additionally monitored with negative stain TEM (Figure S5b).

Finally, for MS measurements, the needle complexes were cross‐linked in replica, each with 0.2–0.3 mM BS3 for 30 min at room temperature, quenched, and subsequently, acetone precipitated to remove undesirable buffer components. Precipitated protein samples were resolubilized in a digestion buffer (8 M urea in 100 mM ammonium bicarbonate) to an estimated protein concentration of 1 mg/mL. Dissolved protein sample was reduced by addition of 1 M dithiothreitol (DTT) to a final concentration of 5 mM. The reaction was incubated at room temperature for 30 min. The free sulfhydryl groups in the sample were then alkylated by adding 500 mM iodoacetamide (final concentration of 15 mM) and incubation at room temperature for 20 min in the dark. After alkylation, additional 1 M DTT was added (total concentration 10 mM) to quench excess of iodoacetamide. Next, protein samples were digested with LysC (at a 50:1 [m/m] protein to protease ratio) at room temperature for 4 h. The sample was then diluted with 100 mM ammonium bicarbonate to reach a urea concentration of 1.5 M. Trypsin was added at a 50:1 (m/m) protein to protease ratio to further digest proteins overnight (~15 h) at room temperature. Resulting peptides were desalted using C18 StageTips (Rappsilber et al., 2007). An aliquot of estimated 0.5 μg peptides was taken from each replica and pooled for protein identification (suppporting information).

For each sample, remaining peptides were fractionated using size exclusion chromatography (SEC) in order to enrich for cross‐linked peptides (Leitner et al., 2014). Peptides were separated using a Superdex™ 30 Increase 3.2/300 column (GE Healthcare) at a flow rate of 10 μL/min. The mobile phase consisted of 30% (v/v) acetonitrile and 0.1% trifluoroacetic acid. The earliest six peptide‐containing fractions (50 μL each) were collected. Solvent was removed using a vacuum concentrator. The fractions were then analyzed by LC–MS/MS.

LC–MS/MS analysis was performed using an Orbitrap Fusion Lumos Tribrid mass spectrometer (Thermo Fisher Scientific), connected to an Ultimate 3000 RSLCnano system (Dionex, Thermo Fisher Scientific). Each SEC fraction was resuspended in 1.6% v/v acetonitrile and 0.1% v/v formic acid and analyzed with LC–MS/MS acquisitions. For the SEC fractions that have sufficient material, a replicated acquisition was carried out. Peptides were injected onto a 50‐cm EASY‐Spray C18 LC column (Thermo Scientific) that is operated at 50 °C column temperature. Mobile phase A consists of water, 0.1% v/v formic acid and mobile phase B consists of 80% v/v acetonitrile and 0.1% v/v formic acid. Peptides were loaded and separated at a flow rate of 0.3 μL/min. Peptides were separated by applying a gradient ranging from 2% to 45% B over 90 min. The gradient was optimized for each fraction. Following the separating gradient, the content of B was ramped to 55% and 95% within 2.5 min each. Eluted peptides were ionized by an EASY‐Spray source (Thermo Scientific) and introduced directly into the mass spectrometer.

The MS data is acquired in the data‐dependent mode with the top‐speed option. For each three‐second acquisition cycle, the full scan mass spectrum was recorded in the Orbitrap with a resolution of 120,000. The ions with a charge state from 3+ to 7+ were isolated and fragmented using high‐energy collisional dissociation For each isolated precursor, one of three collision energy settings (26%, 28%, or 30%) was selected for fragmentation using data‐dependent decision tree based on the *m*/*z* and charge of the precursor. The fragmentation spectra were then recorded in the Orbitrap with a resolution of 50,000. Dynamic exclusion was enabled with a single repeat count and 60‐s exclusion duration.

MS2 peak lists were generated from the raw mass spectrometric data files using the MSConvert module in ProteoWizard (version 3.0.11729). The default parameters were applied, except that Top MS/MS Peaks per 100 Da was set to 20. Precursor and fragment *m*/*z* values were recalibrated. Identification of cross‐linked peptides was carried out using xiSEARCH software (https://www.rappsilberlab.org/software/xisearch) (version 1.7.1) (Mendes et al., 2019). The peak lists were searched against the sequences of 34 target proteins and 34 decoy proteins. The target proteins are 34 proteins that are encoded by the “entry region” of the *Shigella* virulence plasmid (Buchrieser et al., 2000; Cervantes‐Rivera et al., 2020). The decoy proteins were constructed with random amino acid sequences; however, they have the same length as the target proteins and in the sequences, trypsin cleavage amino acids (lysing and arginine) are at the identical position to the target proteins. The following parameters were applied for the search: MS accuracy = 4 ppm; MS2 accuracy = 8 ppm; enzyme = trypsin (with full tryptic specificity); allowed number of missed cleavages = 4; missing monoisotopic peak = 2; cross‐linker = BS3 (the reaction specificity for BS3 was assumed to be for lysine, serine, threonine, tyrosine, and protein N termini); fixed modifications = carbamidomethylation on cysteine; variable modifications = oxidation on methionine and hydrolyzed and amidated BS3. Identified cross‐linked peptide candidates were filtered using xiFDR (Müller et al., 2018). A FDR of 2% on residue‐pair level was applied with “boost between” option selected. A list of identified cross‐linked residue pairs is reported in Table S1.

Only cross‐links that are with a match score of 6 or higher and do not involve the MxiH[SctF] purification tag were taken for modeling. At this threshold, 85% of cross‐links (90 out of 106 that could be mapped to the structure) satisfied the distance threshold of 30 Å when mapped to the part of the needle complex that was built based on high‐resolution regions of the cryo‐EM map.

### 5.5 Integrative modeling

Docking of MxiM[SctG] to MxiD[SctC] was performed using Assembline pipeline (Rantos et al., 2021), which relies on IMP Package version 2.13 and Python Modeling Interface library (Saltzberg et al., 2019). The input structures for the docking constituted 15 copies of MxiM[SctG] (in complex with the last 18 C‐terminal amino acids of MxiD[SctC] α2) PDB ID: 2JW1; (Okon et al., 2008) and the cryo‐EM model of secretin ring determined in this work. A 15:15 stoichiometry between the secretin and the pilotin was chosen based on the T2SS from *E. coli*, which is the only solved structure of a secretin pore in complex with its pilotin (Yin et al., 2018) and it displays this arrangement. The input structures were coarse‐grained to a multiscale representation consisting of two levels: C‐α atom per each residue and a single bead per every 10‐residue stretch. During sampling, the MxiD[SctC] domain was kept fixed, while each of the 15 copies of MxiM[SctG] (including the MxiD[SctC] α2), moved as a single rigid body. The loop between the α1 and α2 helix of MxiD[SctC] was treated as a flexible chain of C‐α atoms. Only cross‐links with a score above 6 were used for modeling (26 cross‐links in total). The conformational sampling was performed using the simulated annealing Monte Carlo method.

The scoring function for the sampling consisted of a linear combination of the clash score preventing the structures from penetrating each other, distance connectivity restraints between the beads, and, depending on the modeling scenario, either cross‐link or both cross‐link and EM restraints. For cross‐link and connectivity restraints, C‐α atom representation was used, whereas the 10‐residue representation was used for the clash score and EM restraints. The cross‐link restraint was implemented as a harmonic function for an upper distance of 30 Å. As there are multiple copies of MxiD and MxiM leading to ambiguity in which equivalent residue pairs were cross‐linked, for each cross‐link the restraint was calculated based on the residue pair giving the shortest cross‐link distance. The EM restraint corresponded to the cross‐correlation of the EM map and the model (*FitRestraint* in IMP). The weights for the scoring function were adjusted to ensure connectivity of the flexible loop and prevent steric clashes, to favor penetration of the EM density over the cross‐links in the scenario when the EM map was used, and to obtain a wide range of conformations and to ensure sampling exhaustiveness. The conformational sampling has been performed using Monte Carlo simulated annealing approach. For modeling with both the cross‐links and EM, 100 modeling runs were performed with each run comprising 120,000 Monte Carlo steps in six simulated annealing stages with temperature decreasing from 100,000 to 1000 (with model scores in the order of 10^6^ units). For modeling based on cross‐link only, 100 modeling runs were performed with each run comprising 60,000 Monte Carlo steps in six simulated annealing stages with temperature decreasing from 10,000 to 100 (with model scores in the order of 10^4^ units). The exhaustiveness of sampling (Figures S19, S20) was performed using the sampling exhaustiveness protocol described by Viswanath et al. (2017).

### 5.6 Structure analysis and representation

EM maps and atomic structures were visualized using UCSF Chimera (Pettersen et al., 2004) and ChimeraX (Goddard et al., 2018; Pettersen et al., 2021). The cross‐links were visualized using xiNET (Combe et al., 2015) and Xlink Analyzer (Kosinski et al., 2015). Protein interfaces were calculated with the “Protein interfaces, surfaces and assemblies” service PISA at the European Bioinformatics Institute (http://www.ebi.ac.uk/pdbe/prot_int/pistart.html) (Krissinel & Henrick, 2007).

## AUTHOR CONTRIBUTIONS

**Lara Flacht:** Formal analysis (equal); investigation (equal); visualization (equal); writing – original draft (equal); writing – review and editing (equal). **Michele Lunelli:** Data curation (equal); formal analysis (equal); investigation (equal); methodology (equal); visualization (equal); writing – original draft (equal); writing – review and editing (equal). **Karol Kaszuba:** Data curation (equal); formal analysis (equal); methodology (equal); visualization (equal); writing – original draft (equal). **Zhou Angel Chen:** Data curation (equal); investigation (equal); methodology (equal); writing – review and editing (equal). **Francis O’. Reilly:** Investigation (equal); writing – review and editing (supporting). **Juri Rappsilber:** Resources (equal); writing – review and editing (supporting). **Jan Kosinski:** Conceptualization (equal); data curation (equal); methodology (equal); project administration (equal); resources (equal); supervision (equal); writing – review and editing (equal).

## CONFLICT OF INTERESTS STATEMENT

The authors declare no competing interests.

## Supporting information


**Data S1:** Supporting Information

## Data Availability

Electron Microscopy Data Bank: EMD‐15700, EMD‐15701, EMD‐15702, EMD‐15703. Protein Data Bank: 8AXK, 8AXL, 8AXN. The mass spectrometry data is deposited in jPost (Okuda et al., 2017) with the project ID JPST001433 (PRIDE ID: PXD030646). The integrative model of MxiM[SctG] docked to the secretin ring will be deposited at PDB‐DEV database upon publication.
