# Supplementary material for: Integrative structural analysis of the type III secretion system needle complex from Shigella flexneri
Source: Protein Sci. 2023 Apr 1;32(4):e4595. doi: 10.1002/pro.4595 (PMC10019453; doi:10.1002/pro.4595)
Supplement: Supplementary file 1 — Data S1: Supporting Information [file PRO-32-e4595-s001.docx]

# Supplementary Information

## Supplementary methods

Mass spectrometry protein identification

The BS3 cross-liked type III secretion system needle complex was digested as described on the Method and Material. Estimate 1 ug peptides was subject to LC-MS/MS for protein identification.

Peptides were resuspended in 1.6% v/v acetonitrile 0.1% v/v formic acid. LC-MS/MS analysis was performed using an Orbitrap Fusion Lumos Tribrid mass spectrometer (Thermo Fisher Scientific), connected to an Ultimate 3000 RSLCnano system (Dionex, Thermo Fisher Scientific). Peptides were injected onto a 50-centimetre EASY-Spray C18 LC column (Thermo Scientific) that is operated at 50 °C column temperature. Mobile phase A consists of water, 0.1% v/v formic acid and mobile phase B consists of 80% v/v acetonitrile and 0.1% v/v formic acid. Peptides were loaded and separated at a flowrate of 0.3 μl/min. Peptides were separated by a series of linear gradients: first by applying a gradient from 2% to 35% B in 90 minutes, then from 35% to 45% B in 15 minutes, from 45% to 55% B in 3 minutes and finally from 55% to 95% B in 2 minutes. Eluted peptides were ionized by an EASY-Spray source (Thermo Scientific) and introduced directly into the mass spectrometer.

The MS data is acquired in the data-dependent mode with the top-speed option. For each three-second acquisition cycle, the full scan mass spectrum was recorded in the Orbitrap with a resolution of 120,000. The ions with a charge state from 2+ to 7+ were isolated and fragmented using higher-energy collisional dissociation (HCD) with 30% collision energy. The fragmentation spectra were then recorded in the Orbitrap with a resolution of 30,000. Dynamic exclusion was enabled with single repeat count and 60 second exclusion duration.

MS raw data was processed using MaxQuant (version 2.1.0.0) for protein identification. MS2 peak lists were searched against the protein sequence database of Shigella flexneri (GenBank_CP037923.fasta; GenBank_CP037924.fasta). The default search parameters were applied for the search except: enzyme = trypsin; allowed number of missed cleavages = 2; fixed modifications = carbamidomethylation on cysteine; variable modifications = oxidation on methionine and hydrolyzed and amidated BS3 on amino acid lysine, serine, tyrosine and Threonine.

The mass spectrometry proteomics data have been deposited to the ProteomeXchange Consortium via the PRIDE^[1]^ partner repository with the dataset identifier PXD038227 and 10.6019/PXD038227.

[1] Perez-Riverol Y, Bai J, Bandla C, Hewapathirana S, García-Seisdedos D, Kamatchinathan S, Kundu D, Prakash A, Frericks-Zipper A, Eisenacher M, Walzer M, Wang S, Brazma A, Vizcaíno JA (2022). The PRIDE database resources in 2022: A Hub for mass spectrometry-based proteomics evidences. Nucleic Acids Res 50(D1):D543-D552 (PubMed ID: 34723319).

## Supporting Figures


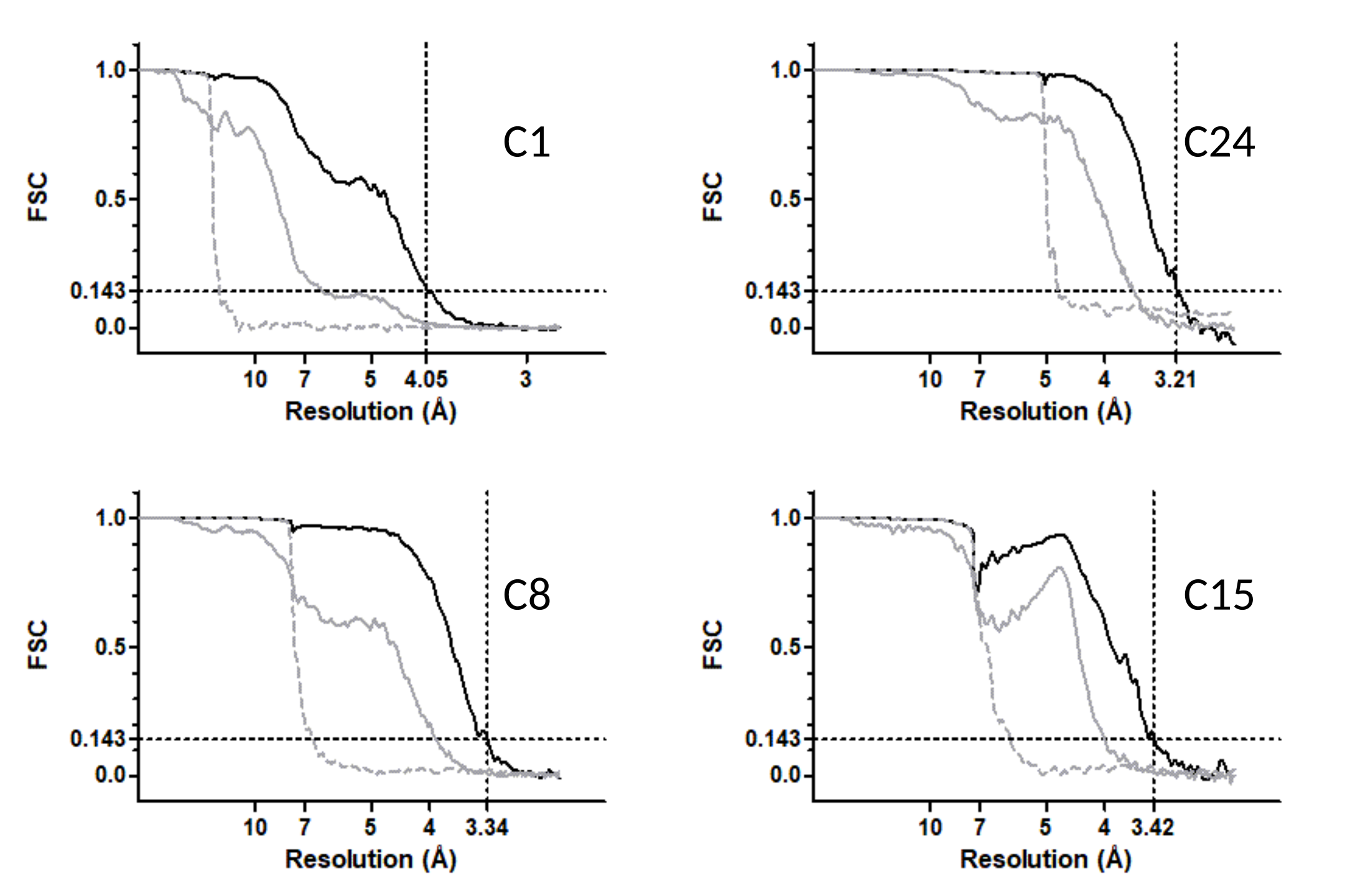


## **Sup. Fig. 1:** Fourier shell correlation curves.

Gold-standard Fourier shell correlation curves for the cryo-EM reconstructions reported in the manuscript (see also Table 1). The gray continuous curve represents the unmasked FSC, the gray dashed curve shows the FSC between phase-randomized maps, while the black curve shows the masked FSC corrected with the procedure implemented in relion_postprocess (described in Chen et al. Ultramicroscopy Volume 135, December 2013, pages 24-35). The reported resolution of the maps is obtained from the corrected masked FSC. The symmetry imposed in the reconstructions is indicated: full needle complex without symmetry imposed (C1); inner membrane ring with C24 symmetry; inner membrane ring and connector with C8 symmetry; outer membrane ring with C15 symmetry.


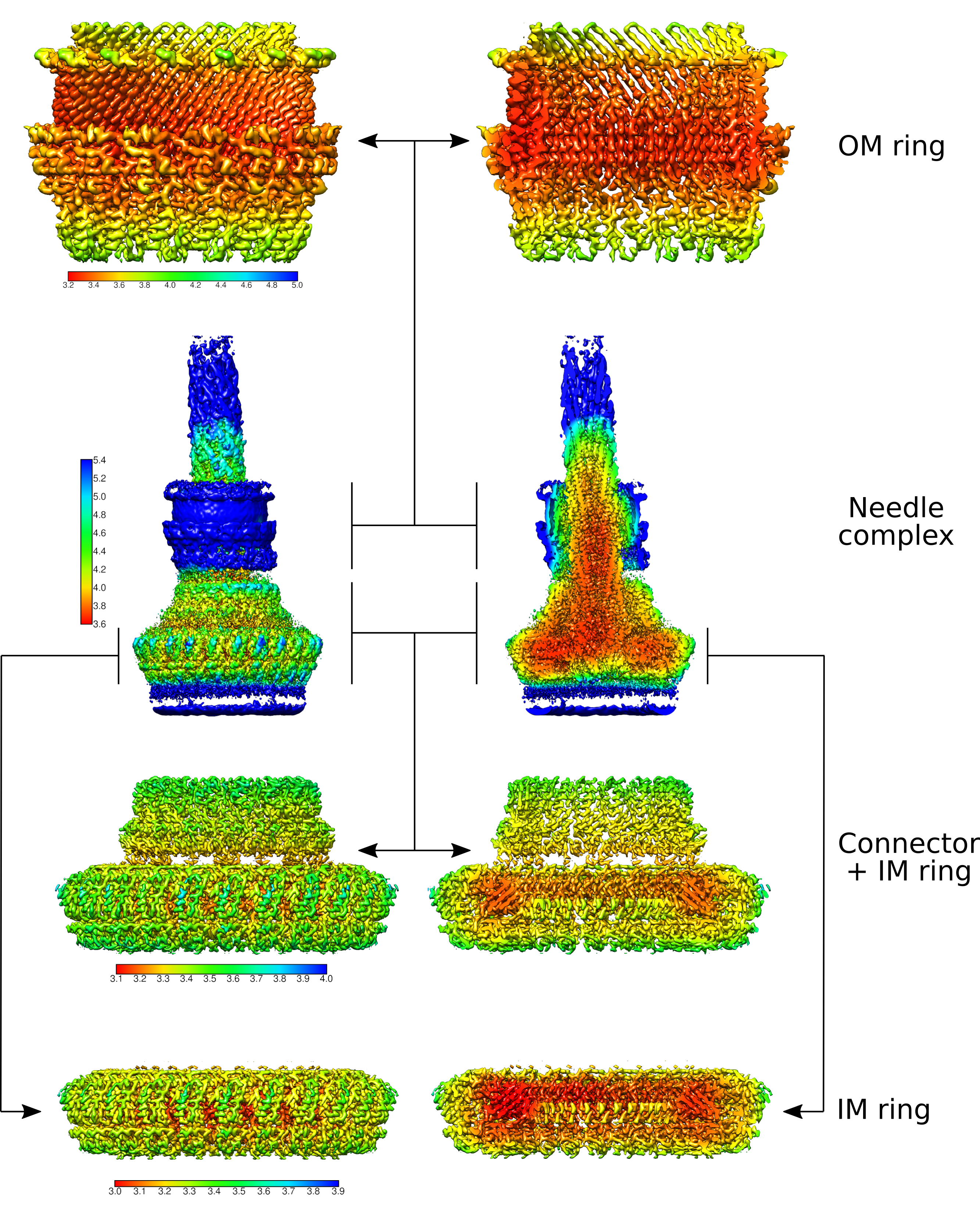


## **Sup. Fig. 2: Local resolution.**

Locally-filtered maps colored according to the local resolution of the reconstructions reported in the manuscript (see also Table 1). Side view (left) and cross-section along the vertical axis (right) of the OM ring map with C15 symmetry, fullneedle complex, IM ring and connector with C8 symmetry, and IM ring with C24 symmetry (from top to bottom). The color keys show the resolution in Å and are valid for the left as well as the right images.


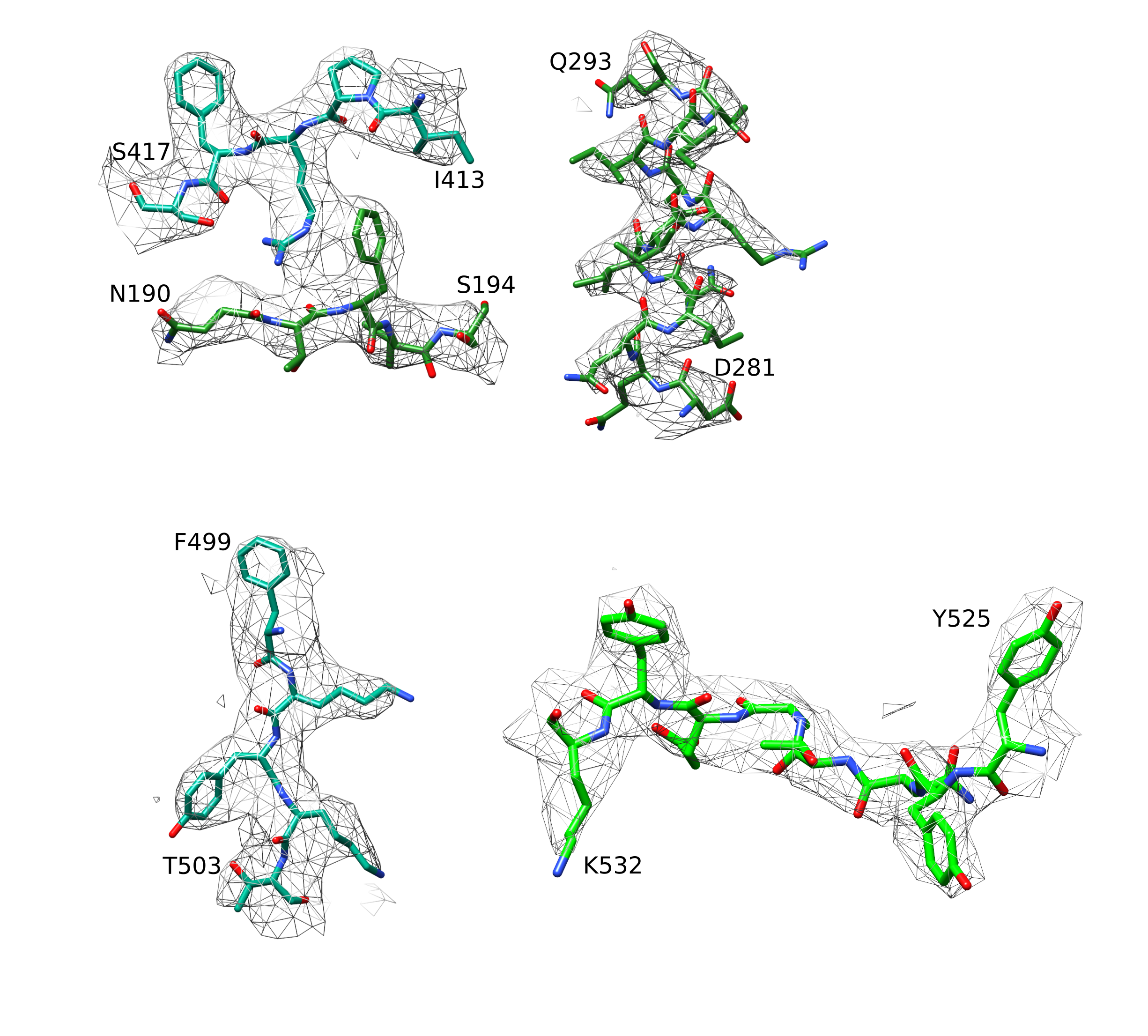


## Sup. Fig. 3: Examples demonstrating the high resolution of the secretin map.

Four selected regions of the secretin map with the model represented as sticks (dark green: N3 domain residues, marine green: secretin domain residues, bright green: S domain residues). The residues at the ends are labeled.


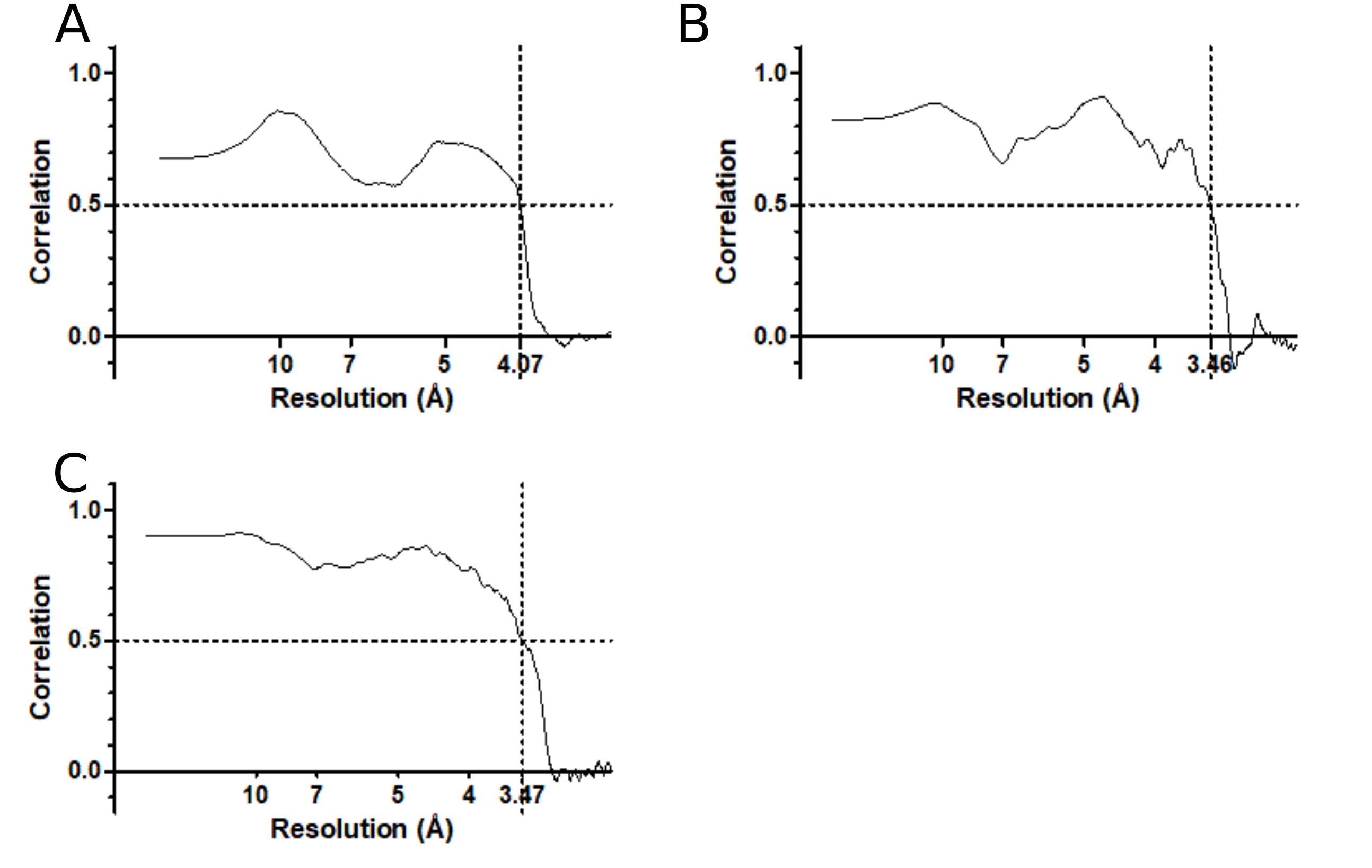


## Sup. Fig. 4: Map-model correlation.

Fourier shell correlation curves between map and atomic model: full needle complex map vs. export apparatus core and initial needle **(A)**, OM ring C15 map vs. secretin pore **(B)**, and IM ring and connector C8 map vs. atomic model **(C)**. The resolution at which the correlation falls below 0.5 is indicated among the labels on the X axis.


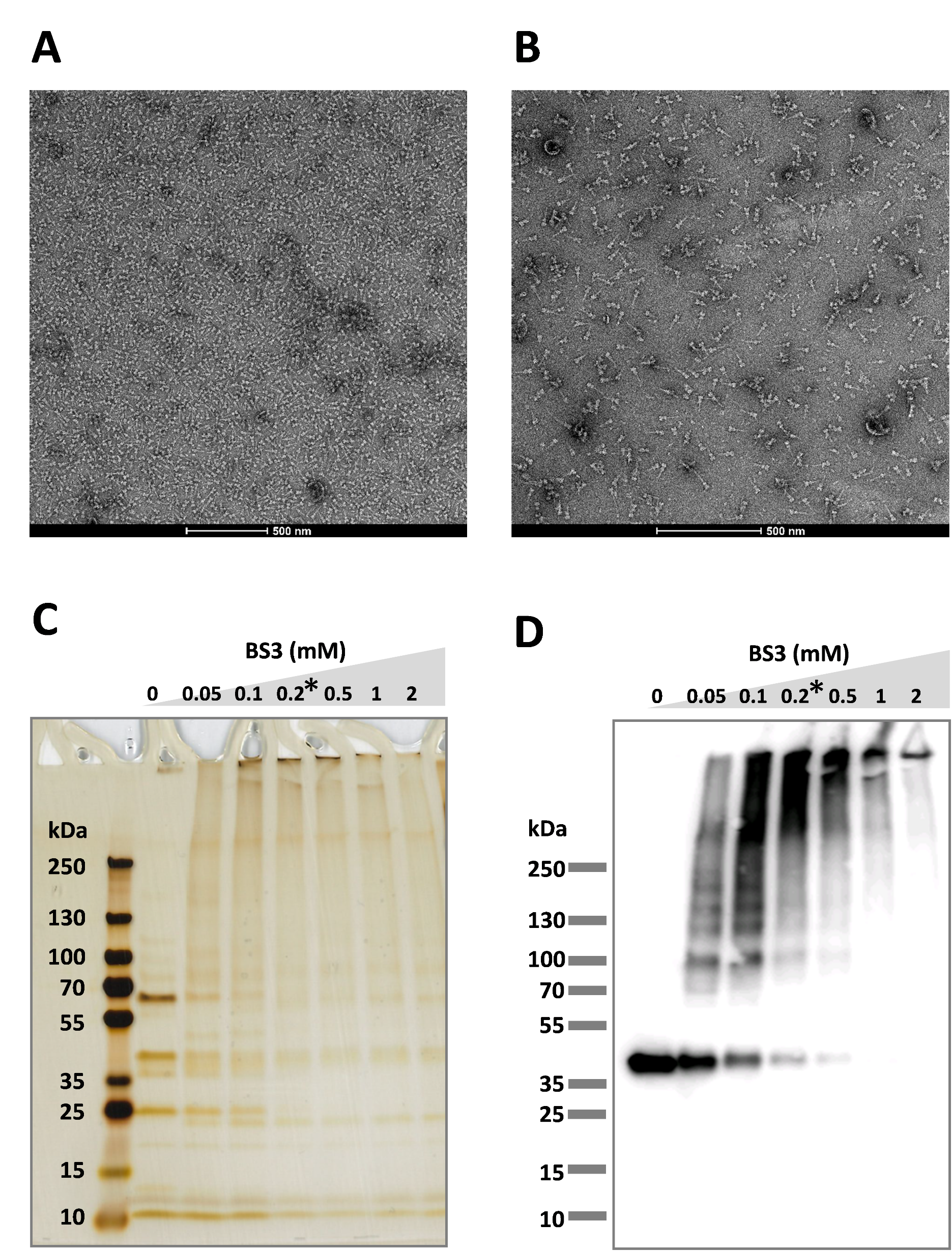


## Sup. Fig. 5: T3SS needle complex sample preparation for cross-linking MS.

Negatively stained electron micrographs of **(A)** a representative elution of isolated T3SS needle complexes after purification and **(B)** after BS3 cross-linking procedure indicating integrity and no major aggregation of particles. Gradient (4-12%) SDS-PAGE of purified needle complexes titrated with increasing BS3 concentrations followed by **(C)** silver staining or **(D)** western blotting with anti-MxiG[SctD] antibody. Upon cross-linking, individual subunit bands disappear and higher molecular weight species become visible. Protein-to-BS3-ratio for MS analysis marked with asterisk.


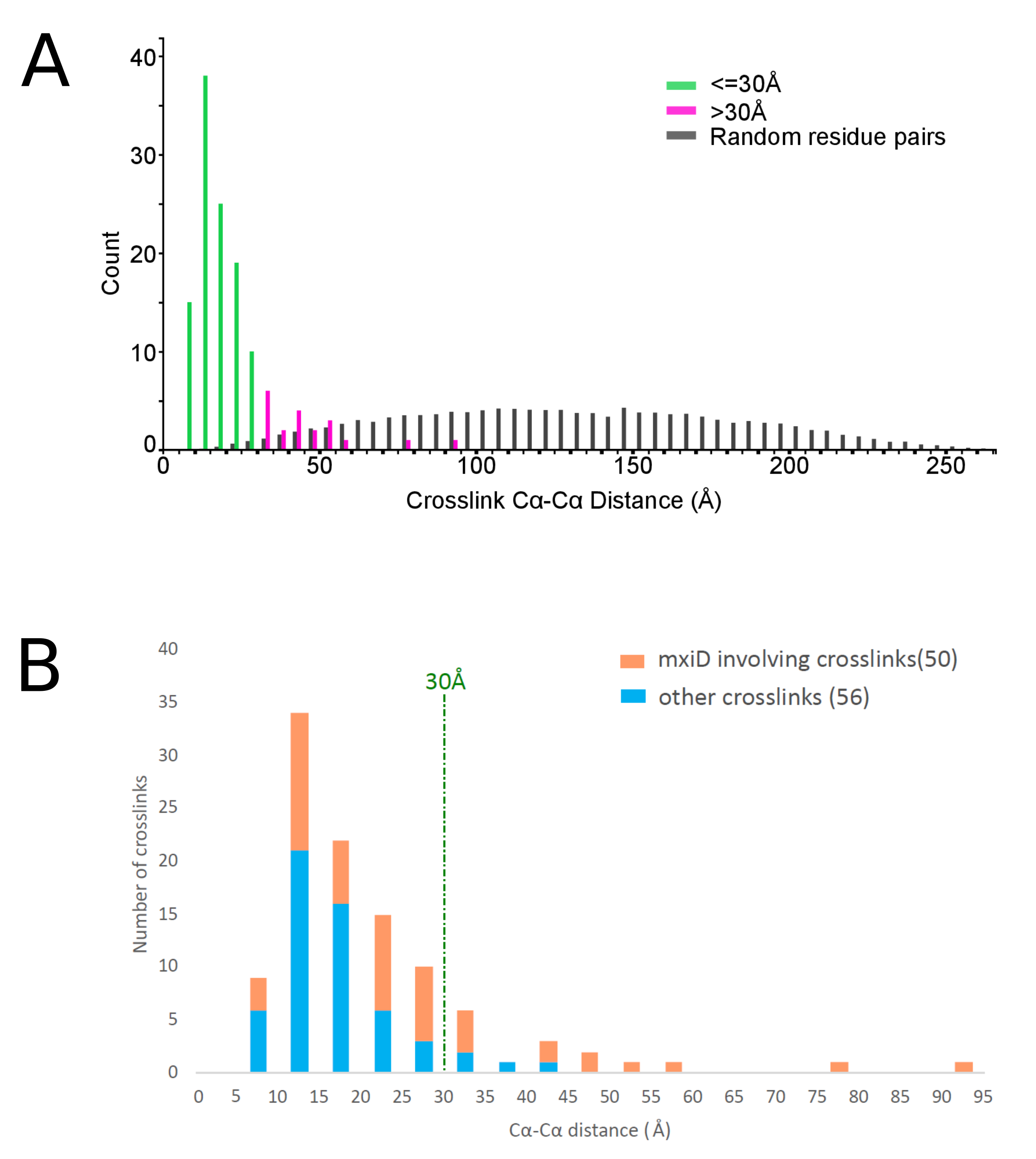


## Sup. Fig. 6: Cross-link distance histograms.

**(A)** In total 364 cross-links were identified with 2% FDR at link level. Among these cross-links, 127 can be displayed in the 3D model of the T3SS needle complex. Distances between cross-linked residues were measured between the Cα atoms. The distribution of the Cα-Cα distances of these cross-links is plotted in the histogram. Green are 107 cross-links with Cα-Cα distances equal or less than 30 Å, pink are 20 cross-links with Cα-Cα distances over 30 Å. Gray show the distance distribution of all cross-linkable residue pairs in the 3D model. **(B)** Cα-Cα distance distribution of 106 cross-links that are used for integrative modelling. Cross-links involving MxiD[SctC] (highlighted in orange) contribute to 75% (12 out of 16) of cross-links that are beyond 30 Å.


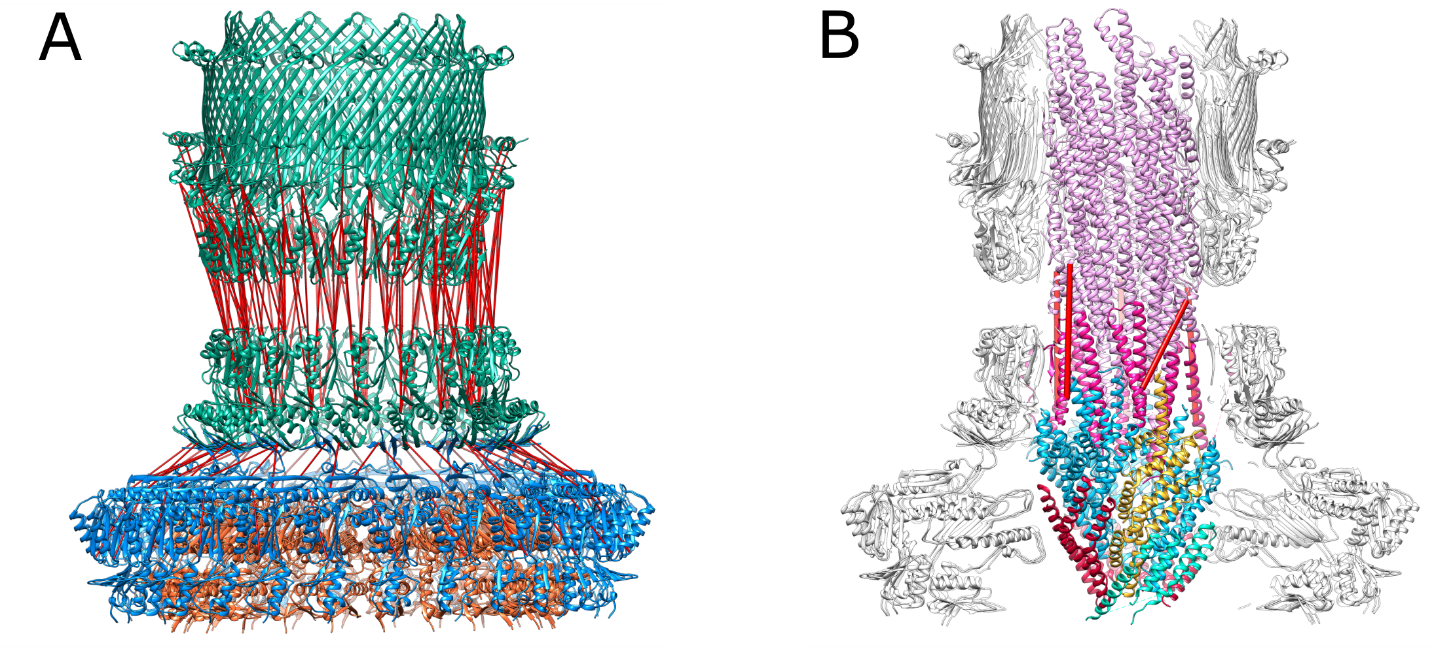


## **Sup. Fig. 7: Cross-links exceeding the distance threshold mapped onto the needle complex obtained by cryo-EM.**

Atomic model of **(A) the basal body** rings (MxiG[SctD], MxiJ[SctJ] and MxiD[SctC]) and **(B) the export apparatus** including the needle (SpaP[SctR], SpaQ[SctS], SpaR[SctT], SpaS[SctU], MxiI[SctI] and MxiH[SctF]) as cartoon representation with cross-links exceeding the distance threshold of 30 Å (red) mapped onto the structures. Cross-links below the threshold are shown in Fig. 2 D, E.


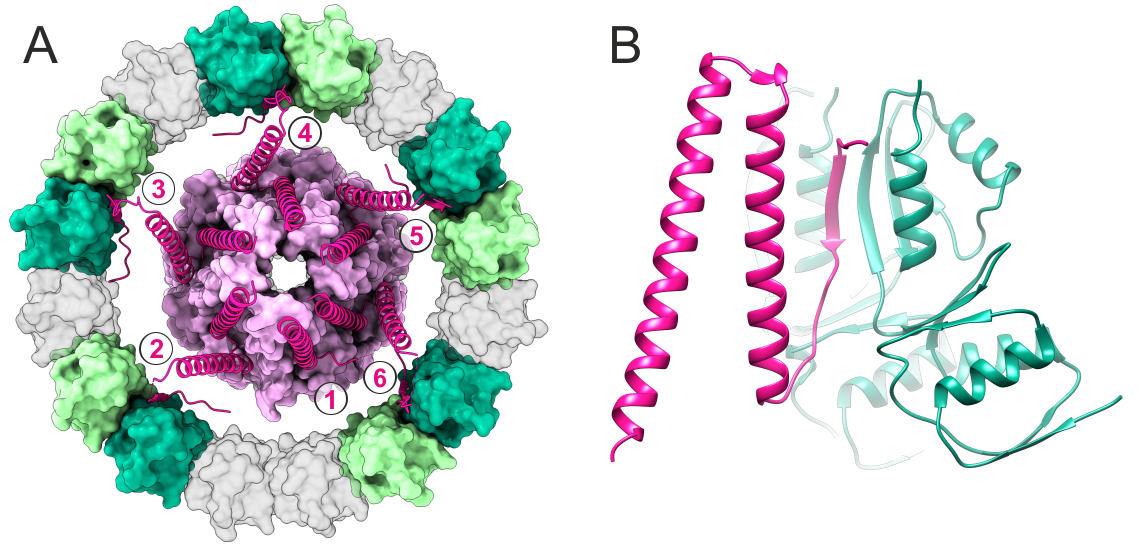


## Sup. Fig. 8: Structure of the inner rod protein MxiI[SctI] and its interfacing proteins.

**(A) Bottom view of the inner rod (MxiI[SctI]) and needle** with subunits shown as surface representation, except for MxiI[SctI] (magenta), which is displayed as cartoon. Proteins subunits interfacing with MxiI[SctI] are opaquely colored and indicated on the right (MxiD[SctC]^1,2,4,5,7,8,10,11,13,14^ light and dark green; MxiH[SctF]^1-12^ pink), highlighting the connecting role of MxiI[SctI] within the needle complex. Proteins that are part of this structure but do not interact with MxiI[SctI] (MxiD[SctC]^3,6,9,12,15,16^, MxiI[SctI]^12-28^) are displayed in transparent gray. Export apparatus subunits (SpaP[SctR], SpaQ[SctS], SpaR[SctT], SpaS[SctU]) are removed to allow view on MxiI[SctI]. **(B) Beta sheet augmentation of MxiI[SctI]^4^** (pink) and a N0,N1-domain of MxiD[SctC] (dark green) in cartoon representation.


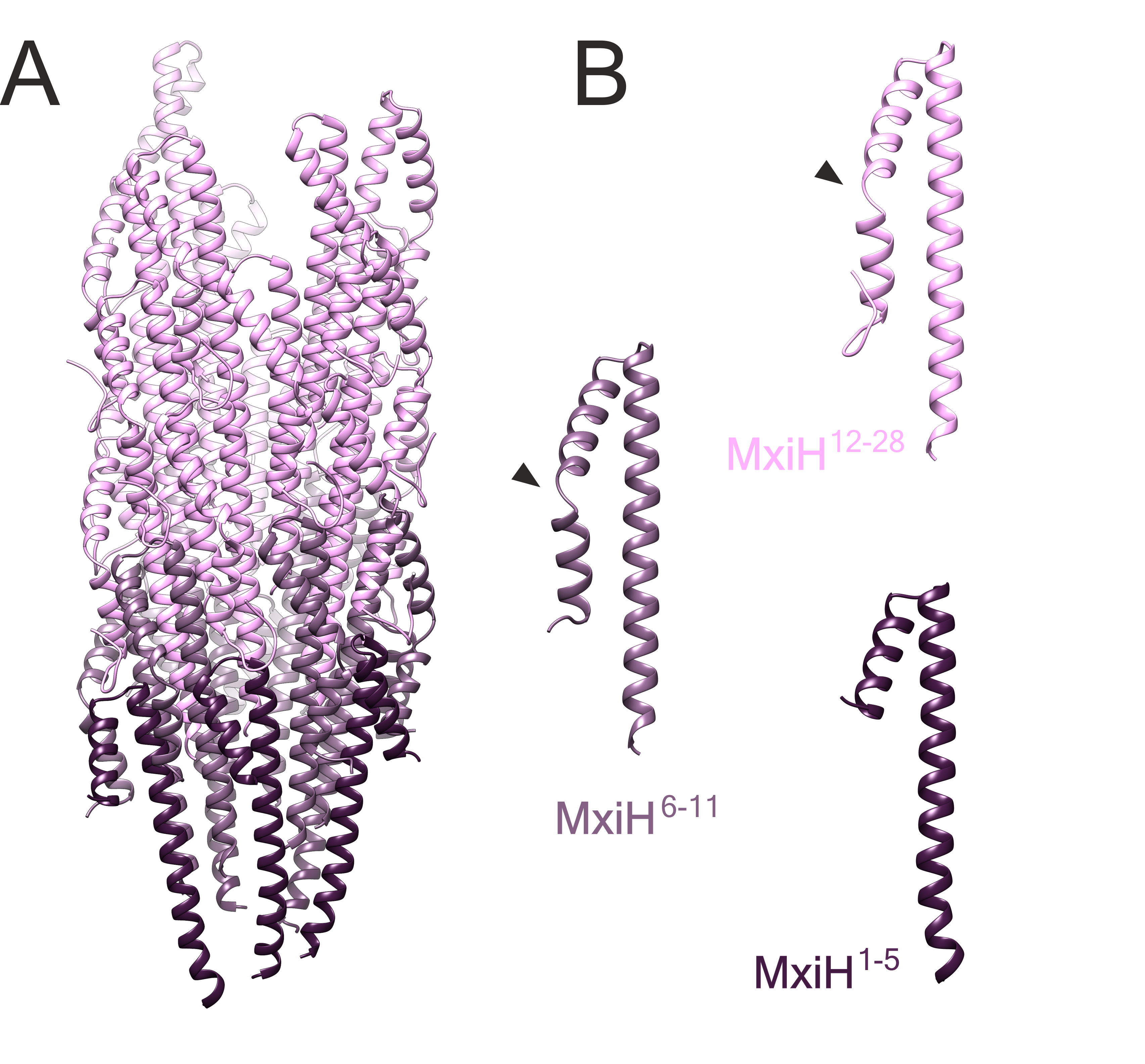


## Sup. Fig. 9: Architecture of the needle (MxiH[SctF]).

**(A) The needle structure in cartoon representation.** Structural differences within the MxiH[SctF] subunits between the first two turns are indicated by different color shades (from proximal to distal: MxiH[SctF]^1-5^ plum, MxiH[SctF]^6-11^lilac, MxiH[SctF]^12-28^ pink). **(B) Structures of individual MxiH[SctF] subunits** displayed and colored as in A. The black arrow indicates the kink within the N-terminal helices.


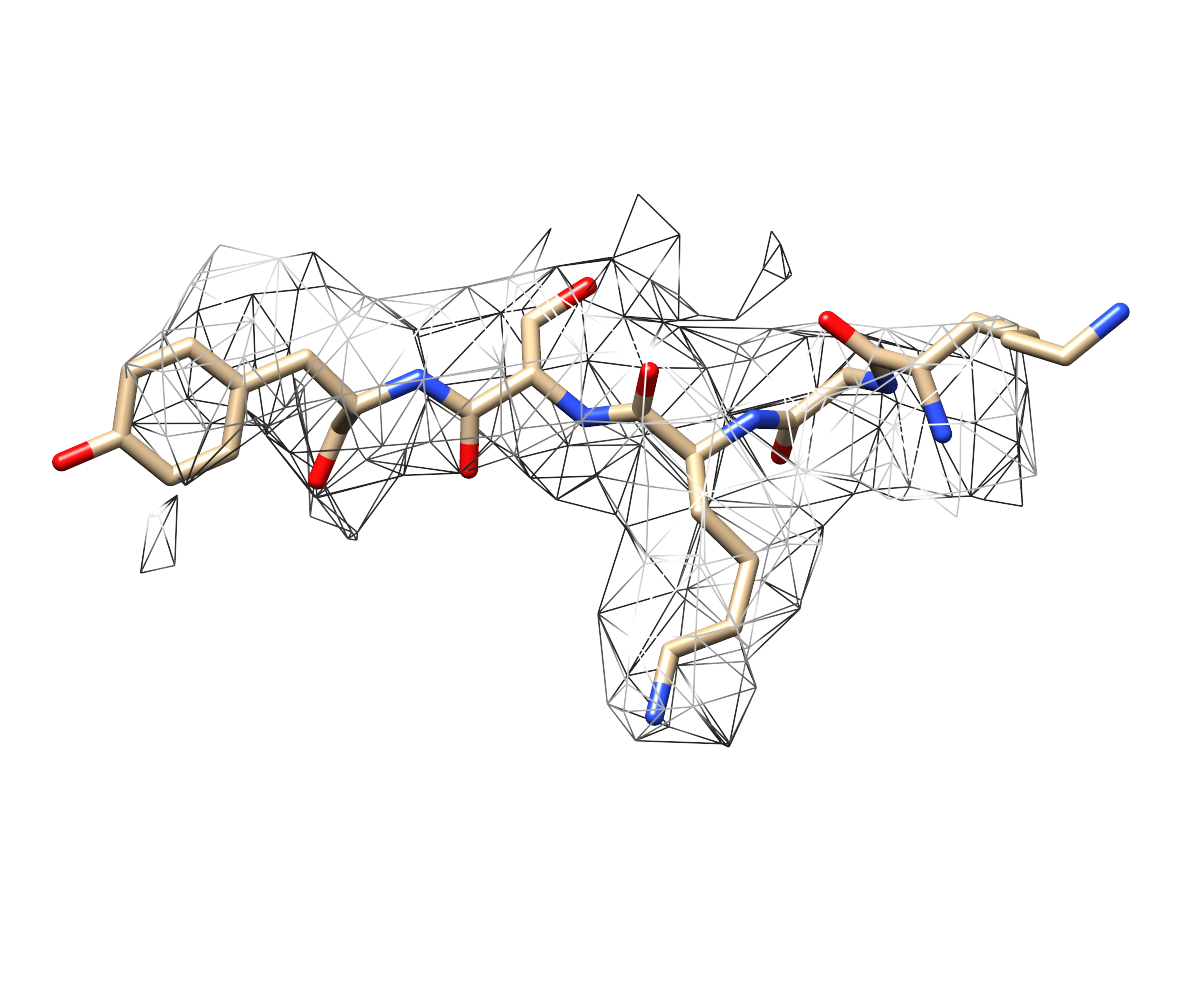


**Sup. Fig. 10: Example of the density map of MxiG.**

The residues K345 - Y349 of the MxiG subunit not involved in β-sheet augmentation with the connector are shown inside the density map.


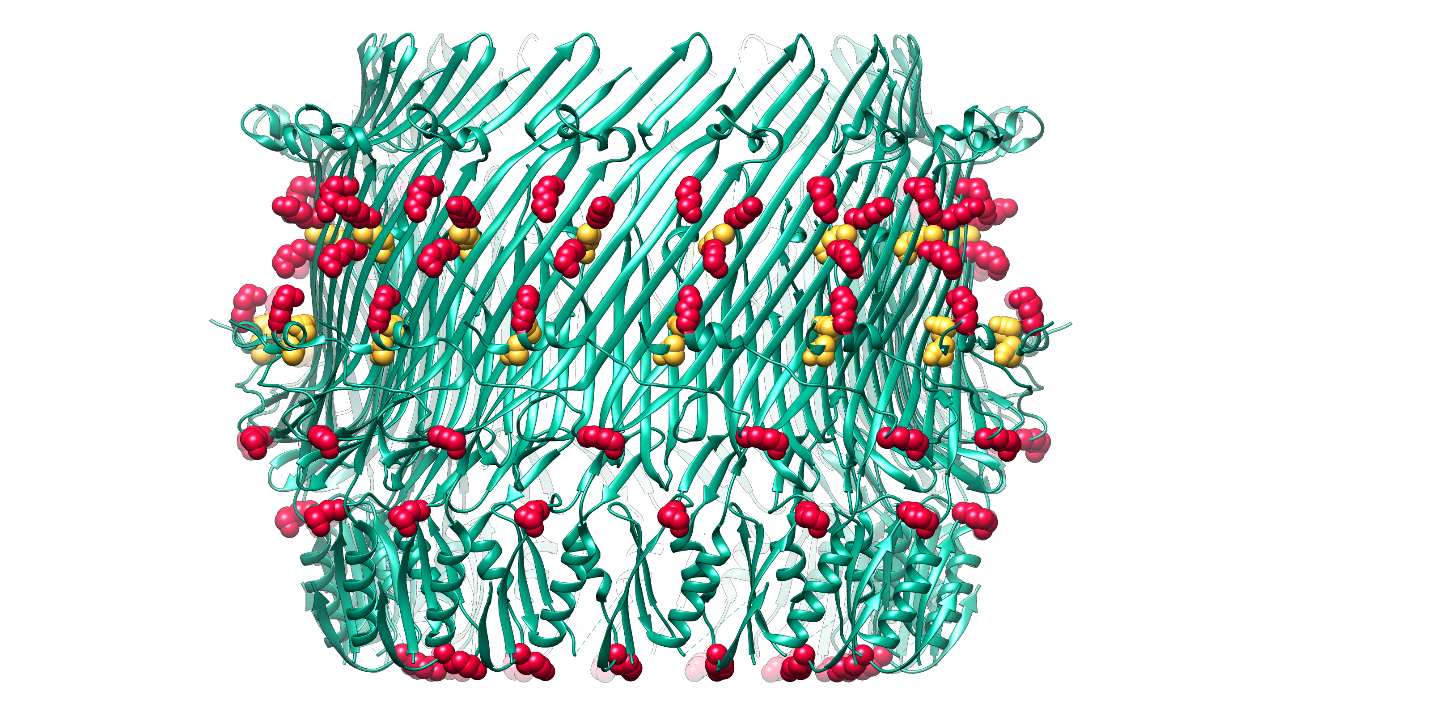


## Sup. Fig. 11: Distribution of cross-links of MxiM[SctG] and C-terminal MxiD[SctC]_549-566_ across the secretin MxiD[SctC]_180‑548_.

Side view of the atomic model of MxiD[SctC]_180-548_ as cartoon representation (green) obtained by cryo-EM. Residues of MxiD[SctC] (K189, K222, K362, K364, K502, K521, K542) cross-linking to both MxiM[SctG] and the residue K558 and S549 belonging to the C-terminal MxiD[SctC] (not present in the EM model) are displayed as red spheres. Residues of MxiD[SctC] (K363, T543, T544) cross-linking solely to MxiM[SctG] are displayed in yellow. All residues of MxiD[SctC] that cross-link with MxiD[SctC]_559-566_ also cross-link with MxiM[SctG].


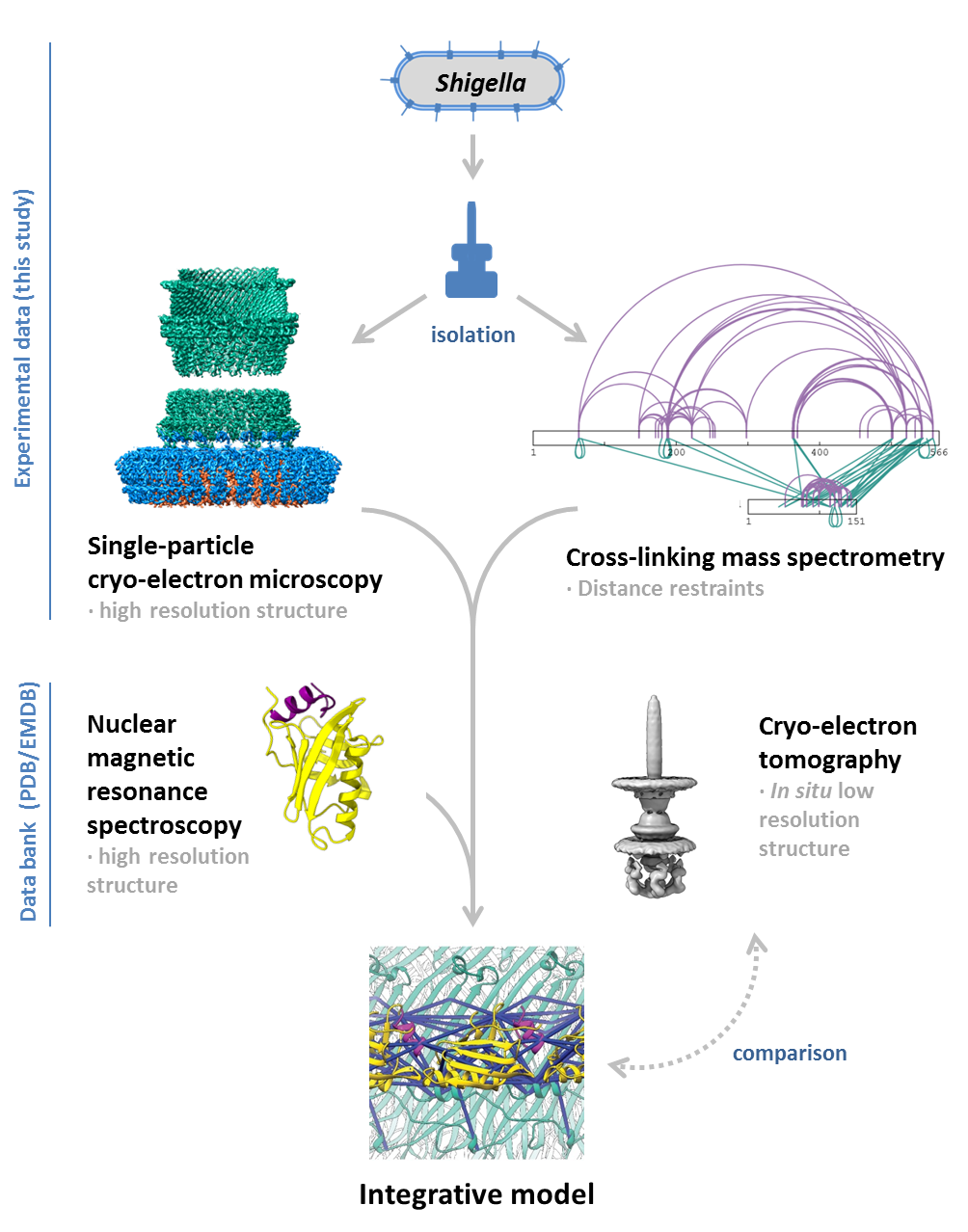


## Sup. Fig. 12: Integrative modeling workflow of this study.

Schematic illustration of the integrative modeling workflow which combines single-particle cryo-EM and cross-linking MS of isolated *Shigella* T3SS needlecomplex with a NMR structure of the pilotin MxiM[SctG] PDB ID 2JW1 (Okon et al., 2008). The obtained integrative model is subsequently compared to cryo-ET structures of the T3SS needle complex.


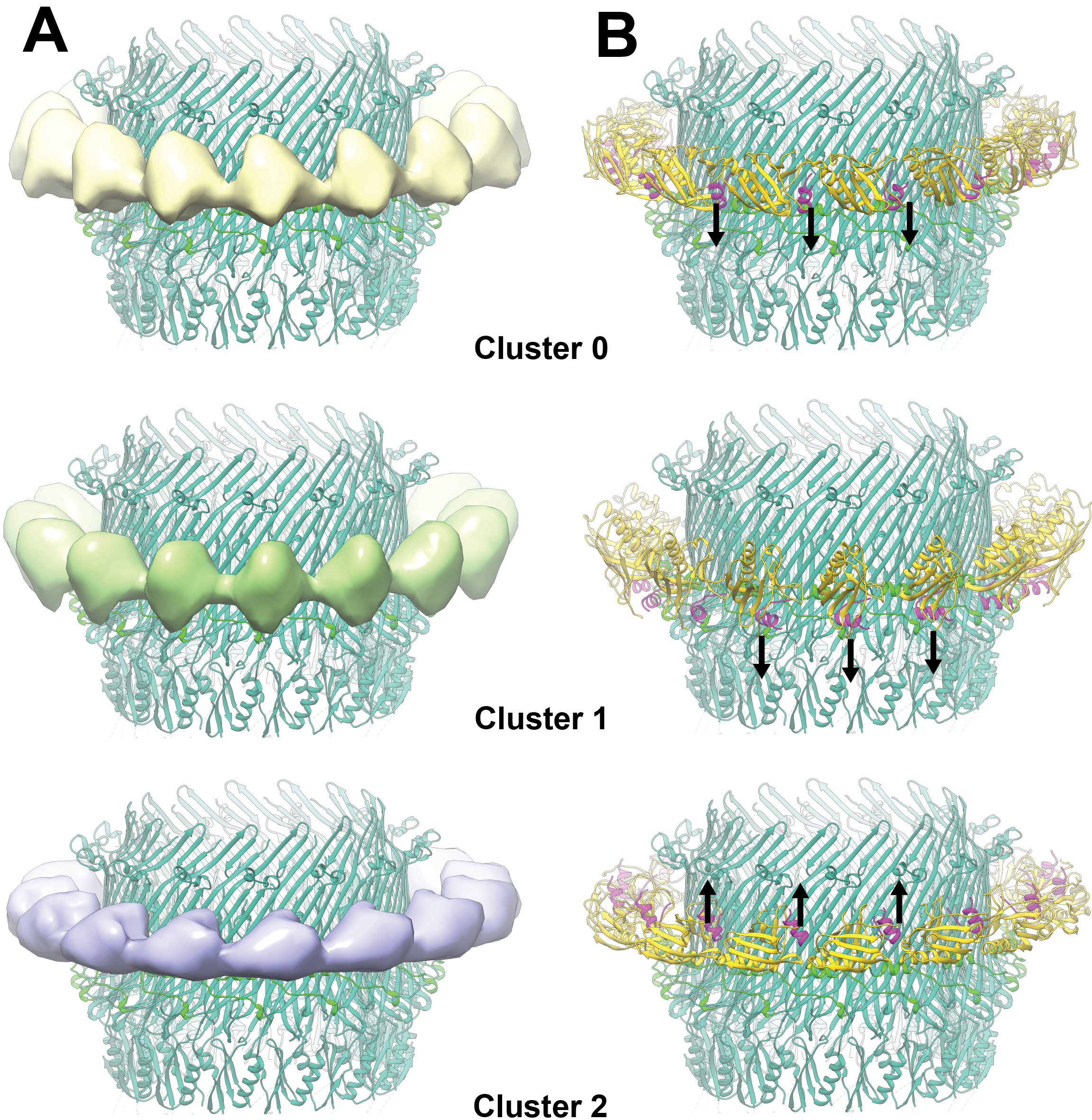


## Sup. Fig. 13: Docking of the pilotin MxiM[SctG] based on cross-links and cryo-EM map.

The cryo-EM structure of the MxiD[SctC]_180-548_ OM ring in cartoon representation (green) and **(A)** **localization probability densities** (solid surfaces) representing the ensemble of the MxiM[SctG] conformations of the three clusters obtained by docking based on cross-links and the cryo-EM map. **(B) Cartoon representation of the respective clusters** with a representative MxiM[SctG] conformation (yellow) in complex with MxiD[SctC]_549-566_ (purple) from (Okon et al., 2008), PDB ID 2JW1. Arrows indicate the orientation of the lipidated MxiM[SctG] N-terminus. The average scores of the clusters, in arbitrary units, are: 2,751,507 (cluster 0), 2,741,471 (cluster 1), 2,752,880 (cluster 2) and the cluster score distributions are not significantly different from each other according to the Kolmogorov–Smirnov test (at the 5% significance level).


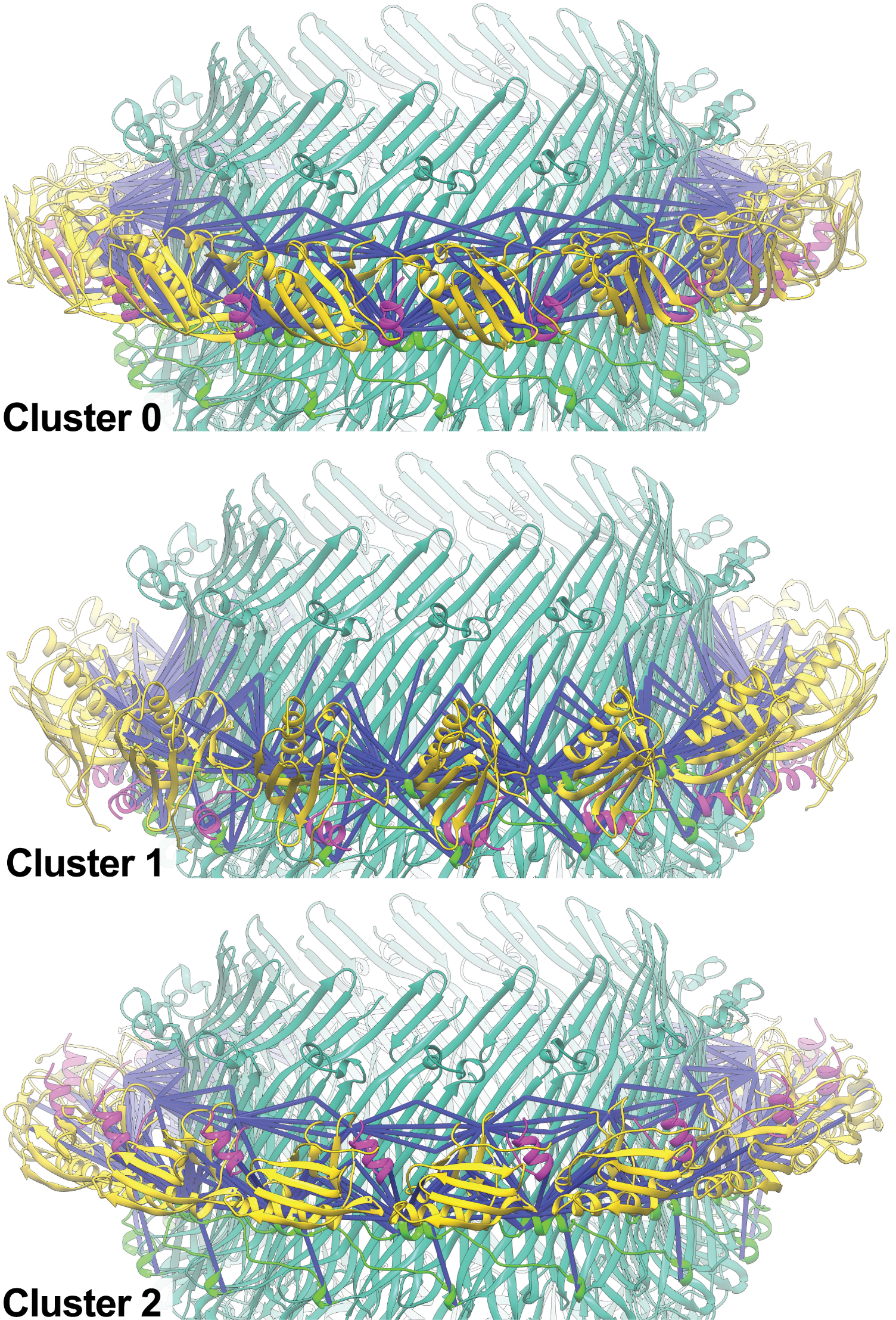


## Sup. Fig. 14: Docking of the pilotin based on the cross-links and cryo-EM map with highlighted cross‑links (<30 Å).

Representative MxiM[SctG] orientations of the three clusters obtained by docking based on cross-links and the cryo-EM map (Sup. Fig. 13). MxiD[SctC]_180-548_ from cryo-EM as cartoon (green) with docked MxiM[SctG] (yellow) in complex with MxiD[SctC]_549-566_ (purple) from (Okon et al., 2008) (PDB ID 2JW1). Cross-links satisfying the distance threshold of 30 Å are displayed in dark blue. The violated cross-links to more distant regions of MxiD[SctC] are not shown for clarity.

## Sup. Fig. 15: Docking of the pilotin MxiM[SctG] based on cross-links only.


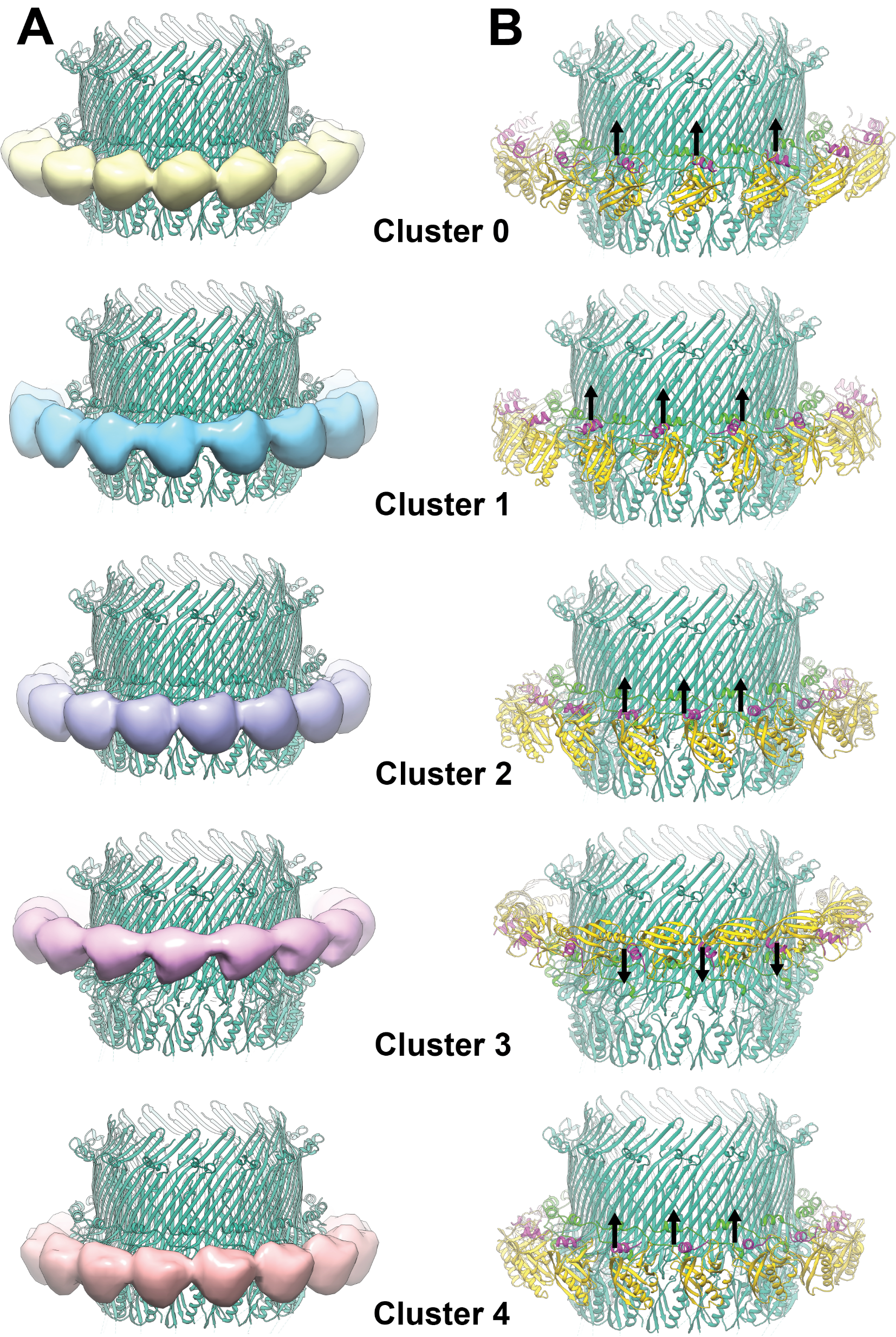


The cryo-EM structure of the MxiD[SctC]_180-548_ OM ring in cartoon representation (green) with (**A)** **localization probability densities** (solid surfaces) representing the ensemble of the MxiM[SctG] conformations of the five clusters. **(B) Cartoon model of the respective clusters** with a representative MxiM[SctG] (yellow) in complex with MxiD[SctC]_549-566_ (purple) from (Okon et al., 2008), PDB ID 2JW1. Arrows indicate the orientation of the lipidated MxiM[SctG] N-terminus. The average scores of the clusters, in arbitrary units, are: 4,186 (cluster 0), 4,203 (cluster 1), 4170 (cluster 2), 4,214 (cluster 3), 4,259 (cluster 4), and the cluster score distributions are not significantly different from each other according to the Kolmogorov–Smirnov test (at the 5% significance level), except for clusters 2 and 4 (p-value=0.045).


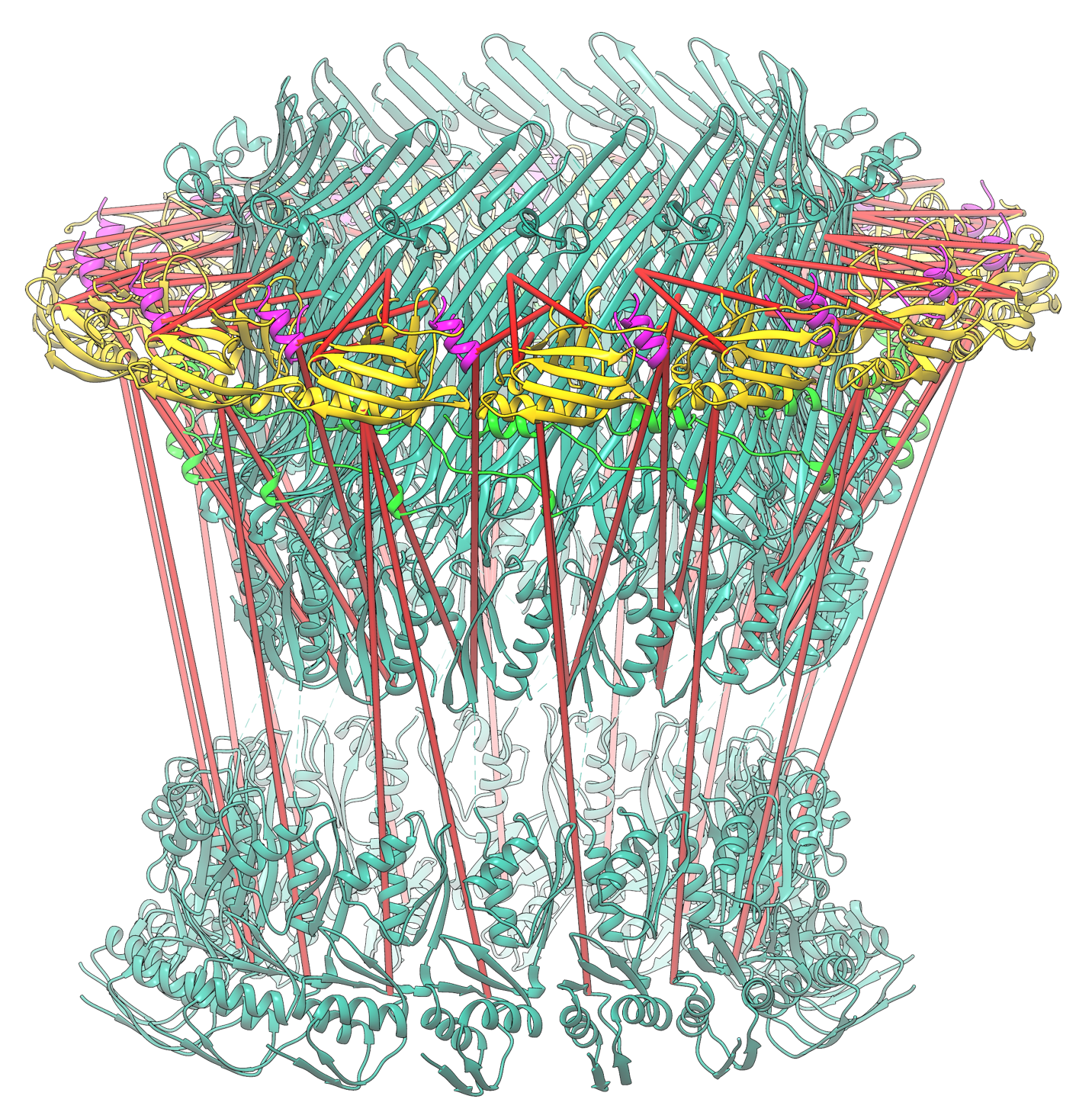


## Sup. Fig. 16: **Cross-links not satisfying the 30 Å distance threshold in any of the models of the** pilotin MxiM[SctG]-secretin complex**.**

Cross-links are mapped to the representative model from the cluster 2 resulting from the docking of the pilotin based on the cross-links and cryo-EM map (Sup. Fig. 14 C). The model is shown in cartoon representation with MxiM[SctG] in yellow and MxiD[SctC]549-566 in purple. Four violated cross-links around the MxiM binding site exceed the threshold for less than 10 Å could be explained by flexibility not accounted in the obtained model ensemble and still support the binding around the S domain (lime green). The three very long cross-links might be false positive identifications or stem from more compact conformation or unspecific aggregation.


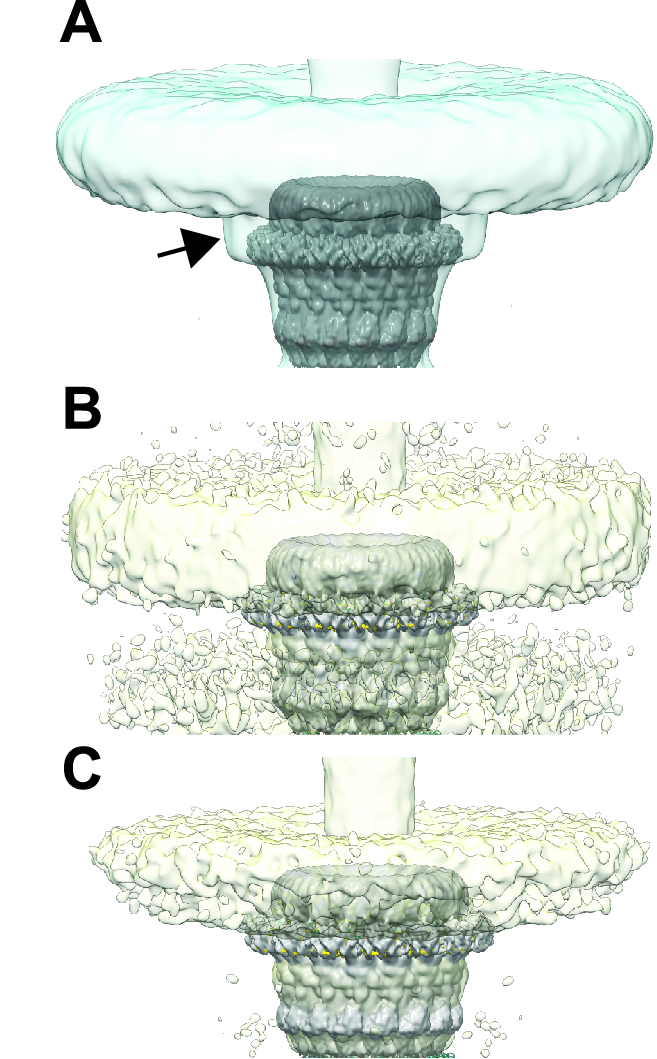


## Sup. Fig. 17: Comparison of cryo-ET densities of the *Shigella* and *Salmonella* T3SS*.*

**(A)** The cryo-EM density of MxiD[SctC] domain (gray solid surface) obtained in this study and superposed on the cryo-ET density (cyan, transparent representation) of the *Shigella* T3SS, EM Data Bank ID: EMD-2667 (Hu et al., 2015). The region of *Shigella*’s tomographic volume, presumably corresponding to individual MxiM[SctG] subunits, which are bound to the MxiD[SctC] domain, is indicated by arrow. On the contrary, the panels **(B)** and **(C)** show tomographic volume of *Salmonella* T3SS, EM Data Bank ID: EMD-8544 (Hu et al., 2017), which does not show such a feature. Figures in panels A, B and C were obtained with the volume contorting thresholds of 0.726, 0.726 and 3.500, respectively.


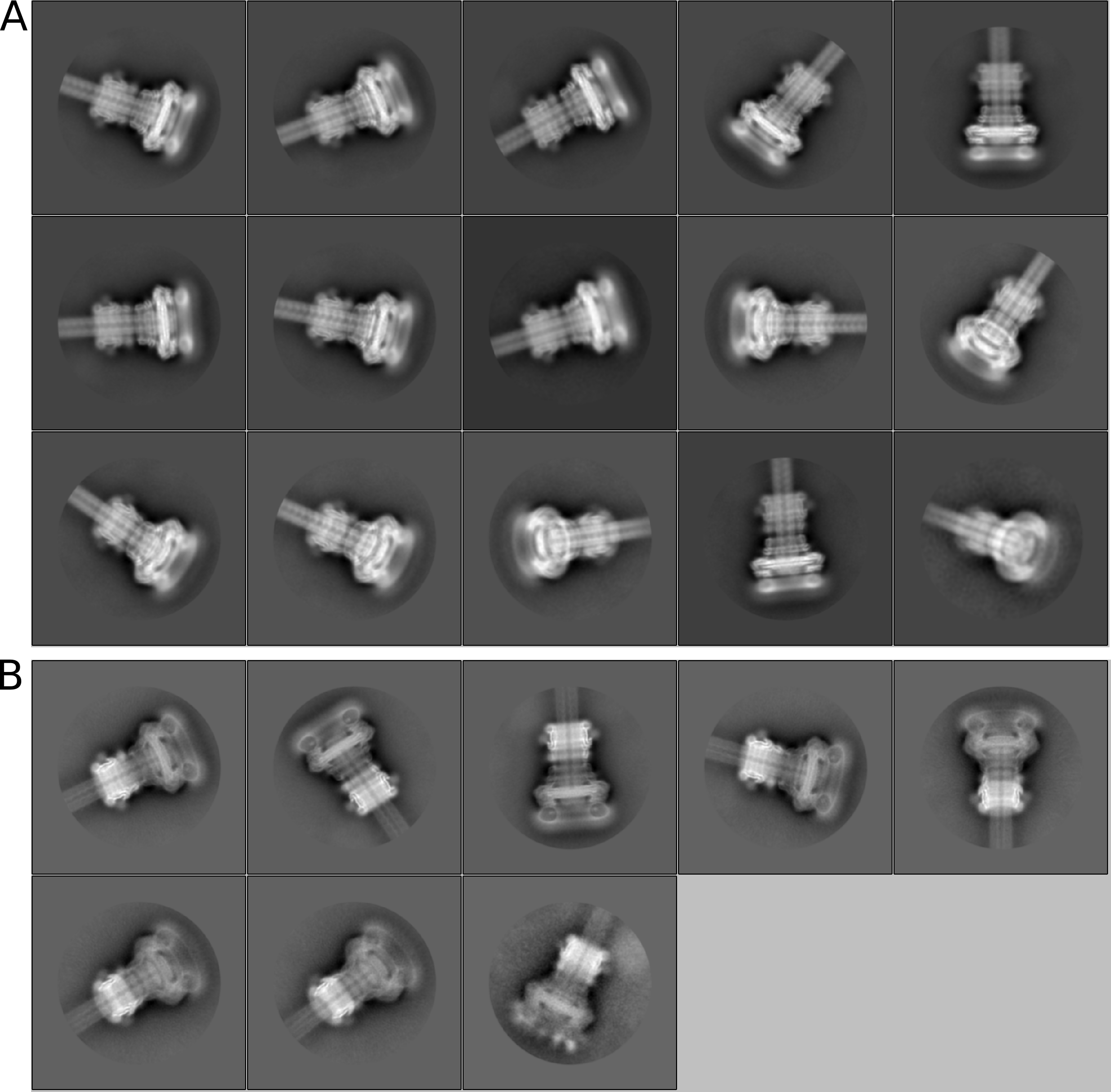


Sup. Fig. 18: **2D class averages.**

Class averages obtained after classification of the image particles **(A)** and of the image particles after partial signal subtraction **(B)**. The partial signal subtraction was performed to enhance the signal of the OM ring.


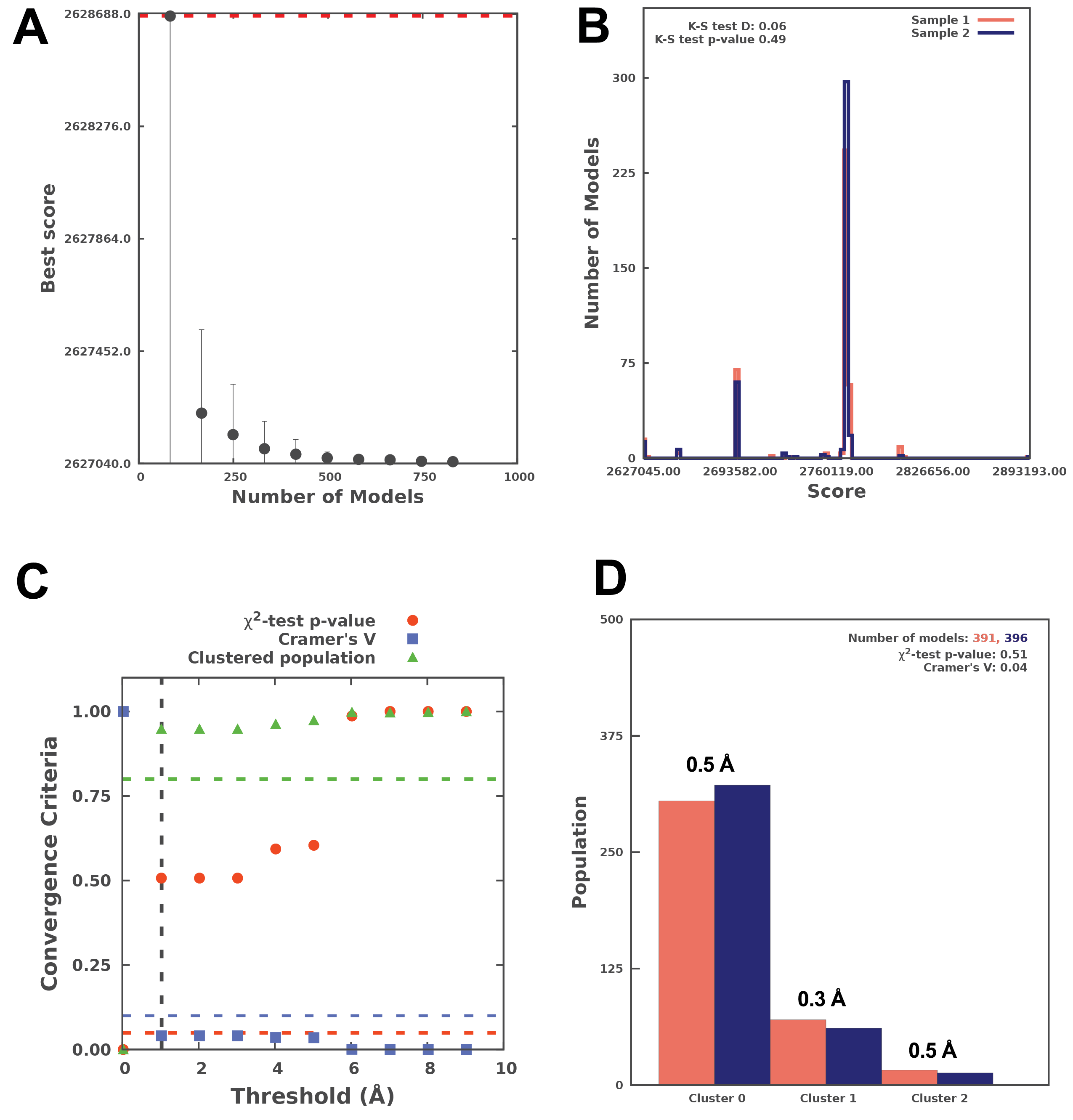


## Sup. Fig. 19: Results for sampling exhaustiveness protocol for the docking run, which was performed with the restraints of cryo-EM map (focused MxiD[SctC] reconstruction) and the restraints of cross-links between MxiM[SctG] and MxiD[SctC].

**(A)** The convergence of the model score for the 800 generated models; the scores do not continue to improve as more models are computed. The error bars represent the standard deviation of the scores. The red dashed line indicates the score level defined by the first data point. **(B)** Testing similarity of model score distributions between two group of models, divided (randomly) into samples 1 (red) and 2 (blue); the difference in distribution of scores is not significant (Kolmogorov-Smirnov two-sample test p value > 0.05) and the magnitude of the difference D is small, that is 0.06; thus, the two score distributions are effectively equal. **(C)** Shown three criteria for determining the sampling precision (y axis), evaluated as a function of the RMSD clustering threshold (x axis). The p value is computed using the χ2 test for homogeneity of proportions (red dots). Second, an effect size for the χ2 test is quantified by the Cramer’s V value (blue squares). The population of models in sufficiently large clusters (containing at least 10 models from each sample) is shown as green triangles. The vertical dashed gray line indicates the RMSD clustering threshold at which three conditions are satisfied (p value > 0.05 (dotted red line), Cramer’s V < 0.10 (dotted blue line), and the population of clustered models > 0.80 (dotted green line)), thus defining the sampling precision of 1.022 Å. **(D)** Populations of sample 1 and 2 models in the clusters are obtained using the RMSD clustering threshold of 1.022 Å. Cluster precision is shown for each cluster.


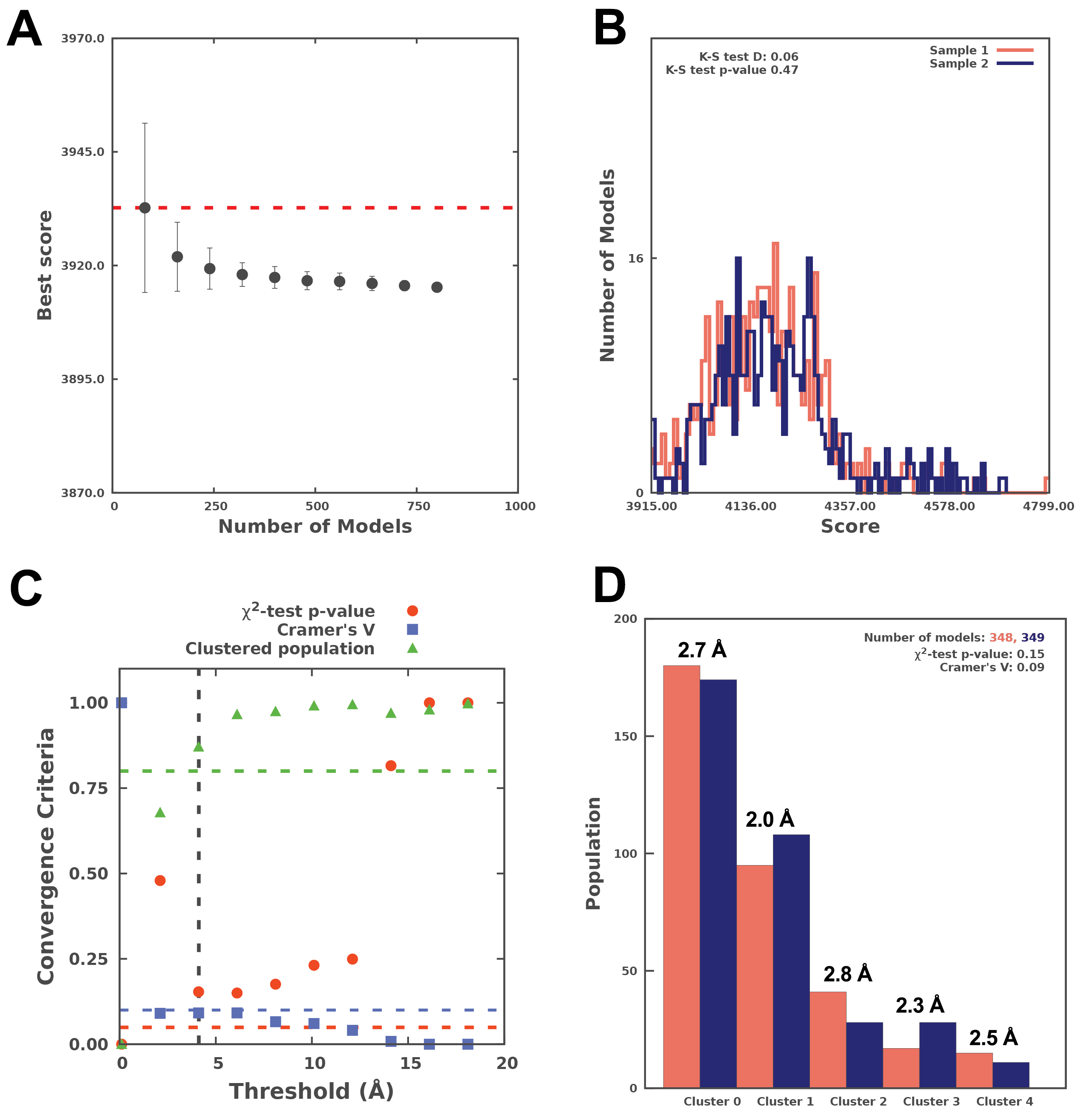


## Sup. Fig. 20: Results for sampling exhaustiveness protocol for the cross-links (only)-guided-docking.

**(A)** The convergence of the model score for the 800 generated models; the scores do not continue to improve as more models are computed. The error bars represent the standard deviation of the scores. The red dashed line indicates the score level defined by the first data point. **(B)** Testing similarity of model score distributions between two group of models, divided (randomly) into samples 1 (red) and 2 (blue); the difference in distribution of scores is not significant (Kolmogorov-Smirnov two-sample test p value > 0.05) and the magnitude of the difference D is small, that is 0.06; thus, the two score distributions are effectively equal. **(C)** Shown three criteria for determining the sampling precision (y axis), evaluated as a function of the RMSD clustering threshold (x axis). The p value is computed using the χ2 test for homogeneity of proportions (red dots). Second, an effect size for the χ2 test is quantified by the Cramer’s V value (blue squares). The population of models in sufficiently large clusters (containing at least 10 models from each sample) is shown as green triangles. The vertical dotted gray line indicates the RMSD clustering threshold at which three conditions are satisfied (p value > 0.05 (dotted red line), Cramer’s V < 0.10 (dotted blue line), and the population of clustered models > 0.80 (dotted green line)), thus defining the sampling precision of 4.108 Å. **(D)** Populations of sample 1 and 2 models in the clusters are obtained using the RMSD clustering threshold of 4.108 Å. Cluster precision is shown for each cluster.

## Sup. Table 1: List of cross-links

| **Protein1** | **Protein Name 1** | **SeqPos1** | **LinkedRes1** | **Protein2** | **Protein Name 2** | **SeqPos2** | **LinkedRes2** | **Highest Score** | **Match Count** | **Decoy Type** | **Link Type** | **Cα-Cα distance in the 3D model** | **From Chain** | **To Chain** | **Used for modelling** |
| --- | --- | --- | --- | --- | --- | --- | --- | --- | --- | --- | --- | --- | --- | --- | --- |
| TagQCC34415.1 | TagMxiH | 2 | A | TagQCC34415.1 | TagMxiH | 11 | K | 21.2 | 4 | TT | Homomultimeric |  |  |  | FALSE |
| TagQCC34415.1 | TagMxiH | 84 | K | TagQCC34415.1 | TagMxiH | 87 | K | 19.92 | 9 | TT | Homomultimeric | 15.91 | u | v | TRUE |
| QCC34408.1 | mxiD | 364 | K | QCC34408.1 | mxiD | 542 | K | 19.27 | 6 | TT | Self | 17.98 | Z15 | Z1 | TRUE |
| QCC34416.1 | mxiG | 293 | K | QCC34416.1 | mxiG | 353 | K | 19.1 | 5 | TT | Self | 12.26 | X22 | X22 | TRUE |
| QCC34408.1 | mxiD | 364 | K | QCC34408.1 | mxiD | 502 | K | 18.64 | 8 | TT | Self | 11.62 | Z1 | Z0 | TRUE |
| QCC34410.1 | mxiM | 82 | K | QCC34410.1 | mxiM | 99 | K | 18.24 | 7 | TT | Self |  |  |  | TRUE |
| QCC34408.1 | mxiD | 187 | K | TagQCC34415.1 | TagMxiH | 2 | A | 18 | 5 | TT | Heteromeric |  |  |  | FALSE |
| QCC34413.1 | mxiJ | 143 | K | QCC34413.1 | mxiJ | 178 | K | 17.96 | 6 | TT | Self | 11.26 | Y5 | Y5 | TRUE |
| QCC34416.1 | mxiG | 252 | K | QCC34416.1 | mxiG | 268 | K | 17.49 | 54 | TT | Self | 13.17 | X11 | X11 | TRUE |
| QCC34408.1 | mxiD | 222 | K | TagQCC34415.1 | TagMxiH | 2 | A | 17.45 | 4 | TT | Heteromeric |  |  |  | FALSE |
| QCC34414.1 | mxiI | 1 | M | QCC34414.1 | mxiI | 33 | K | 17.29 | 3 | TT | Self |  |  |  | TRUE |
| QCC34408.1 | mxiD | 187 | K | QCC34408.1 | mxiD | 222 | K | 17.29 | 3 | TT | Self | 26.33 | Z14 | Z13 | TRUE |
| QCC34416.1 | mxiG | 252 | K | TagQCC34415.1 | TagMxiH | 2 | A | 17.26 | 2 | TT | Heteromeric |  |  |  | FALSE |
| QCC34413.1 | mxiJ | 49 | K | QCC34413.1 | mxiJ | 54 | K | 16.71 | 4 | TT | Homomultimeric | 21.57 | Y6 | Y5 | TRUE |
| QCC34413.1 | mxiJ | 54 | K | QCC34413.1 | mxiJ | 54 | K | 16.59 | 8 | TT | Homomultimeric | 14.33 | Y1 | Y22 | TRUE |
| QCC34416.1 | mxiG | 254 | K | QCC34416.1 | mxiG | 268 | K | 16.55 | 9 | TT | Self | 8.75 | X23 | X23 | TRUE |
| TagQCC34415.1 | TagMxiH | 2 | A | TagQCC34415.1 | TagMxiH | 40 | T | 16.47 | 4 | TT | Self |  |  |  | FALSE |
| TagQCC34415.1 | TagMxiH | 2 | A | TagQCC34415.1 | TagMxiH | 54 | K | 16.44 | 7 | TT | Self |  |  |  | FALSE |
| TagQCC34415.1 | TagMxiH | 2 | A | TagQCC34415.1 | TagMxiH | 68 | K | 16.29 | 1 | TT | Self |  |  |  | FALSE |
| QCC34408.1 | mxiD | 222 | K | QCC34408.1 | mxiD | 521 | K | 16.06 | 2 | TT | Self | 46.24 | Z10 | Z10 | TRUE |
| QCC34414.1 | mxiI | 1 | M | TagQCC34415.1 | TagMxiH | 87 | K | 15.94 | 4 | TT | Heteromeric |  |  |  | TRUE |
| QCC34408.1 | mxiD | 364 | K | QCC34408.1 | mxiD | 558 | K | 15.93 | 3 | TT | Self |  |  |  | TRUE |
| QCC34408.1 | mxiD | 362 | K | QCC34408.1 | mxiD | 502 | K | 15.89 | 4 | TT | Self | 9.75 | Z3 | Z1 | TRUE |
| TagQCC34415.1 | TagMxiH | 54 | K | TagQCC34415.1 | TagMxiH | 68 | K | 15.85 | 1 | TT | Homomultimeric | 13.8 | p | u | TRUE |
| QCC34408.1 | mxiD | 189 | K | TagQCC34415.1 | TagMxiH | 2 | A | 15.85 | 6 | TT | Heteromeric |  |  |  | FALSE |
| QCC34408.1 | mxiD | 187 | K | QCC34408.1 | mxiD | 298 | K | 15.82 | 3 | TT | Self | 13.68 | Z8 | Z7 | TRUE |
| QCC34410.1 | mxiM | 82 | K | TagQCC34415.1 | TagMxiH | 2 | A | 15.8 | 4 | TT | Heteromeric |  |  |  | FALSE |
| QCC34410.1 | mxiM | 99 | K | TagQCC34415.1 | TagMxiH | 2 | A | 15.66 | 2 | TT | Heteromeric |  |  |  | FALSE |
| QCC34408.1 | mxiD | 171 | K | TagQCC34415.1 | TagMxiH | 2 | A | 15.54 | 2 | TT | Heteromeric |  |  |  | FALSE |
| QCC34416.1 | mxiG | 5 | K | QCC34416.1 | mxiG | 17 | K | 15.33 | 1 | TT | Self |  |  |  | TRUE |
| QCC34408.1 | mxiD | 521 | K | QCC34410.1 | mxiM | 52 | K | 15.29 | 2 | TT | Heteromeric |  |  |  | TRUE |
| QCC34414.1 | mxiI | 33 | K | QCC34414.1 | mxiI | 86 | K | 15.21 | 1 | TT | Self | 18.14 | Q | Q | TRUE |
| QCC34416.1 | mxiG | 5 | K | QCC34416.1 | mxiG | 73 | K | 15.1 | 5 | TT | Self |  |  |  | TRUE |
| TagQCC34415.1 | TagMxiH | 54 | K | TagQCC34415.1 | TagMxiH | 87 | K | 15.01 | 3 | TT | Self | 16.64 | T | e | TRUE |
| QCC34414.1 | mxiI | 33 | K | TagQCC34415.1 | TagMxiH | 2 | A | 14.99 | 3 | TT | Heteromeric |  |  |  | FALSE |
| QCC34410.1 | mxiM | 121 | K | TagQCC34415.1 | TagMxiH | 2 | A | 14.92 | 2 | TT | Heteromeric |  |  |  | FALSE |
| QCC34408.1 | mxiD | 180 | K | TagQCC34415.1 | TagMxiH | 2 | A | 14.86 | 4 | TT | Heteromeric |  |  |  | FALSE |
| QCC34408.1 | mxiD | 189 | K | TagQCC34415.1 | TagMxiH | 11 | K | 14.85 | 3 | TT | Heteromeric |  |  |  | FALSE |
| QCC34416.1 | mxiG | 240 | K | QCC34416.1 | mxiG | 268 | K | 14.63 | 2 | TT | Self | 14.7 | X10 | X10 | TRUE |
| QCC34408.1 | mxiD | 502 | K | QCC34410.1 | mxiM | 130 | K | 14.61 | 2 | TT | Heteromeric |  |  |  | TRUE |
| QCC34408.1 | mxiD | 189 | K | QCC34408.1 | mxiD | 222 | K | 14.57 | 4 | TT | Self | 31.85 | Z14 | Z13 | TRUE |
| TagQCC34415.1 | TagMxiH | 2 | A | TagQCC34415.1 | TagMxiH | 28 | S | 14.54 | 1 | TT | Self |  |  |  | FALSE |
| TagQCC34415.1 | TagMxiH | 2 | A | TagQCC34415.1 | TagMxiH | 19 | T | 14.53 | 2 | TT | Self |  |  |  | FALSE |
| QCC34410.1 | mxiM | 115 | K | QCC34410.1 | mxiM | 139 | K | 14.49 | 3 | TT | Self |  |  |  | TRUE |
| QCC34408.1 | mxiD | 187 | K | QCC34408.1 | mxiD | 521 | K | 14.44 | 2 | TT | Self | 22.31 | Z1 | Z0 | TRUE |
| TagQCC34415.1 | TagMxiH | 2 | A | TagQCC34415.1 | TagMxiH | 87 | K | 14.44 | 2 | TT | Self |  |  |  | FALSE |
| QCC34408.1 | mxiD | 521 | K | QCC34408.1 | mxiD | 542 | K | 14.42 | 1 | TT | Self | 22.96 | Z8 | Z9 | TRUE |
| QCC34400.1 | Spa24 | 105 | K | QCC34414.1 | mxiI | 1 | M | 14.42 | 2 | TT | Heteromeric |  |  |  | TRUE |
| QCC34414.1 | mxiI | 1 | M | TagQCC34415.1 | TagMxiH | 84 | K | 14.42 | 1 | TT | Heteromeric |  |  |  | TRUE |
| QCC34408.1 | mxiD | 171 | K | QCC34408.1 | mxiD | 189 | K | 14.38 | 2 | TT | Self | 43.1 | Z0 | Z0 | TRUE |
| QCC34414.1 | mxiI | 1 | M | QCC34414.1 | mxiI | 86 | K | 14.35 | 2 | TT | Self |  |  |  | TRUE |
| QCC34414.1 | mxiI | 25 | S | TagQCC34415.1 | TagMxiH | 2 | A | 14.29 | 1 | TT | Heteromeric |  |  |  | FALSE |
| QCC34408.1 | mxiD | 148 | K | QCC34408.1 | mxiD | 189 | K | 14.22 | 1 | TT | Self | 47.55 | Z13 | Z14 | TRUE |
| QCC34408.1 | mxiD | 542 | K | QCC34410.1 | mxiM | 130 | K | 14.19 | 4 | TT | Heteromeric |  |  |  | TRUE |
| QCC34408.1 | mxiD | 364 | K | QCC34410.1 | mxiM | 130 | K | 14.19 | 4 | TT | Heteromeric |  |  |  | TRUE |
| QCC34408.1 | mxiD | 148 | K | QCC34408.1 | mxiD | 180 | K | 14.11 | 2 | TT | Self | 21.37 | Z13 | Z14 | TRUE |
| QCC34416.1 | mxiG | 293 | K | QCC34416.1 | mxiG | 362 | K | 14.02 | 3 | TT | Self | 34.25 | X13 | X13 | TRUE |
| QCC34408.1 | mxiD | 456 | K | TagQCC34415.1 | TagMxiH | 2 | A | 14.02 | 2 | TT | Heteromeric |  |  |  | FALSE |
| QCC34408.1 | mxiD | 222 | K | TagQCC34415.1 | TagMxiH | 54 | K | 14.01 | 1 | TT | Heteromeric | 6.71 | Z15 | T | TRUE |
| QCC34416.1 | mxiG | 293 | K | QCC34416.1 | mxiG | 369 | K | 13.99 | 3 | TT | Self |  |  |  | TRUE |
| QCC34408.1 | mxiD | 467 | K | QCC34408.1 | mxiD | 521 | K | 13.96 | 1 | TT | Self | 12.56 | Z1 | Z3 | TRUE |
| QCC34416.1 | mxiG | 2 | S | QCC34416.1 | mxiG | 73 | K | 13.92 | 8 | TT | Self |  |  |  | TRUE |
| QCC34410.1 | mxiM | 127 | K | TagQCC34415.1 | TagMxiH | 2 | A | 13.89 | 1 | TT | Heteromeric |  |  |  | FALSE |
| QCC34400.1 | Spa24 | 105 | K | TagQCC34415.1 | TagMxiH | 2 | A | 13.81 | 2 | TT | Heteromeric |  |  |  | FALSE |
| QCC34408.1 | mxiD | 521 | K | TagQCC34415.1 | TagMxiH | 2 | A | 13.81 | 2 | TT | Heteromeric |  |  |  | FALSE |
| QCC34408.1 | mxiD | 222 | K | QCC34410.1 | mxiM | 115 | K | 13.74 | 2 | TT | Heteromeric |  |  |  | TRUE |
| QCC34408.1 | mxiD | 222 | K | QCC34408.1 | mxiD | 298 | K | 13.72 | 1 | TT | Self | 34.75 | Z0 | Z0 | TRUE |
| QCC34408.1 | mxiD | 180 | K | QCC34408.1 | mxiD | 222 | K | 13.71 | 2 | TT | Self | 10.56 | Z14 | Z13 | TRUE |
| QCC34416.1 | mxiG | 293 | K | TagQCC34415.1 | TagMxiH | 2 | A | 13.71 | 1 | TT | Heteromeric |  |  |  | FALSE |
| TagQCC34415.1 | TagMxiH | 2 | A | TagQCC34415.1 | TagMxiH | 38 | T | 13.67 | 2 | TT | Self |  |  |  | FALSE |
| TagQCC34415.1 | TagMxiH | 2 | A | TagQCC34415.1 | TagMxiH | 65 | Y | 13.66 | 1 | TT | Self |  |  |  | FALSE |
| QCC34408.1 | mxiD | 521 | K | QCC34408.1 | mxiD | 558 | K | 13.65 | 1 | TT | Self |  |  |  | TRUE |
| QCC34410.1 | mxiM | 99 | K | QCC34410.1 | mxiM | 121 | K | 13.65 | 2 | TT | Self |  |  |  | TRUE |
| QCC34408.1 | mxiD | 542 | K | QCC34410.1 | mxiM | 77 | K | 13.64 | 1 | TT | Heteromeric |  |  |  | TRUE |
| QCC34408.1 | mxiD | 298 | K | TagQCC34415.1 | TagMxiH | 2 | A | 13.6 | 5 | TT | Heteromeric |  |  |  | FALSE |
| QCC34414.1 | mxiI | 1 | M | TagQCC34415.1 | TagMxiH | 54 | K | 13.58 | 2 | TT | Heteromeric |  |  |  | TRUE |
| QCC34408.1 | mxiD | 64 | K | QCC34416.1 | mxiG | 362 | K | 13.58 | 3 | TT | Heteromeric | 14.8 | Z7 | X0 | TRUE |
| QCC34408.1 | mxiD | 222 | K | QCC34414.1 | mxiI | 1 | M | 13.58 | 1 | TT | Heteromeric |  |  |  | TRUE |
| TagQCC34415.1 | TagMxiH | 68 | K | TagQCC34415.1 | TagMxiH | 87 | K | 13.57 | 1 | TT | Self | 10.81 | b | q | TRUE |
| QCC34400.1 | Spa24 | 126 | K | QCC34416.1 | mxiG | 347 | K | 13.56 | 1 | TT | Heteromeric |  |  |  | TRUE |
| QCC34408.1 | mxiD | 148 | K | QCC34408.1 | mxiD | 521 | K | 13.46 | 1 | TT | Self | 59.43 | Z15 | Z15 | TRUE |
| QCC34416.1 | mxiG | 293 | K | QCC34416.1 | mxiG | 313 | K | 13.44 | 2 | TT | Self | 16.54 | X5 | X4 | TRUE |
| TagQCC34415.1 | TagMxiH | 2 | A | TagQCC34415.1 | TagMxiH | 84 | K | 13.42 | 4 | TT | Self |  |  |  | FALSE |
| QCC34410.1 | mxiM | 77 | K | TagQCC34415.1 | TagMxiH | 2 | A | 13.41 | 2 | TT | Heteromeric |  |  |  | FALSE |
| QCC34408.1 | mxiD | 64 | K | QCC34408.1 | mxiD | 521 | K | 13.4 | 1 | TT | Self | 92.38 | Z1 | Z0 | TRUE |
| QCC34414.1 | mxiI | 1 | M | QCC34414.1 | mxiI | 20 | S | 13.34 | 1 | TT | Self |  |  |  | TRUE |
| QCC34416.1 | mxiG | 246 | K | QCC34416.1 | mxiG | 252 | K | 13.33 | 6 | TT | Homomultimeric | 26.53 | X16 | X17 | TRUE |
| QCC34413.1 | mxiJ | 178 | K | QCC34416.1 | mxiG | 347 | K | 13.32 | 8 | TT | Heteromeric | 13.28 | Y4 | X8 | TRUE |
| QCC34410.1 | mxiM | 121 | K | QCC34410.1 | mxiM | 139 | K | 13.31 | 4 | TT | Self |  |  |  | TRUE |
| QCC34408.1 | mxiD | 363 | K | QCC34410.1 | mxiM | 130 | K | 13.17 | 1 | TT | Heteromeric |  |  |  | TRUE |
| TagQCC34415.1 | TagMxiH | 2 | A | TagQCC34415.1 | TagMxiH | 57 | S | 13.15 | 9 | TT | Self |  |  |  | FALSE |
| QCC34414.1 | mxiI | 1 | M | QCC34416.1 | mxiG | 293 | K | 13.13 | 2 | TT | Heteromeric |  |  |  | TRUE |
| QCC34408.1 | mxiD | 64 | K | TagQCC34415.1 | TagMxiH | 2 | A | 13.12 | 3 | TT | Heteromeric |  |  |  | FALSE |
| QCC34408.1 | mxiD | 542 | K | QCC34410.1 | mxiM | 115 | K | 13.11 | 2 | TT | Heteromeric |  |  |  | TRUE |
| QCC34408.1 | mxiD | 189 | K | QCC34408.1 | mxiD | 298 | K | 13.05 | 4 | TT | Self | 11 | Z9 | Z9 | TRUE |
| QCC34410.1 | mxiM | 82 | K | QCC34410.1 | mxiM | 130 | K | 13.02 | 1 | TT | Self |  |  |  | TRUE |
| TagQCC34415.1 | TagMxiH | 84 | K | TagQCC34415.1 | TagMxiH | 84 | K | 12.99 | 3 | TT | Homomultimeric | 15.63 | s | t | TRUE |
| QCC34408.1 | mxiD | 521 | K | QCC34410.1 | mxiM | 130 | K | 12.96 | 1 | TT | Heteromeric |  |  |  | TRUE |
| QCC34416.1 | mxiG | 246 | K | TagQCC34415.1 | TagMxiH | 2 | A | 12.94 | 2 | TT | Heteromeric |  |  |  | FALSE |
| QCC34414.1 | mxiI | 16 | S | TagQCC34415.1 | TagMxiH | 2 | A | 12.94 | 1 | TT | Heteromeric |  |  |  | FALSE |
| QCC34408.1 | mxiD | 189 | K | QCC34408.1 | mxiD | 521 | K | 12.91 | 2 | TT | Self | 19.73 | Z9 | Z9 | TRUE |
| QCC34408.1 | mxiD | 558 | K | QCC34410.1 | mxiM | 82 | K | 12.89 | 2 | TT | Heteromeric |  |  |  | TRUE |
| QCC34410.1 | mxiM | 99 | K | QCC34410.1 | mxiM | 130 | K | 12.87 | 1 | TT | Self |  |  |  | TRUE |
| QCC34410.1 | mxiM | 91 | K | QCC34410.1 | mxiM | 121 | K | 12.86 | 4 | TT | Self |  |  |  | TRUE |
| QCC34408.1 | mxiD | 189 | K | QCC34414.1 | mxiI | 1 | M | 12.79 | 2 | TT | Heteromeric |  |  |  | TRUE |
| QCC34429.1 | VirB | 48 | K | QCC34429.1 | VirB | 121 | K | 12.72 | 3 | TT | Self |  |  |  | TRUE |
| TagQCC34415.1 | TagMxiH | 2 | A | TagQCC34415.1 | TagMxiH | 51 | K | 12.7 | 1 | TT | Self |  |  |  | FALSE |
| QCC34408.1 | mxiD | 298 | K | QCC34408.1 | mxiD | 521 | K | 12.7 | 3 | TT | Self | 12.32 | Z1 | Z1 | TRUE |
| QCC34416.1 | mxiG | 246 | K | QCC34416.1 | mxiG | 268 | K | 12.69 | 5 | TT | Self | 15.29 | X22 | X22 | TRUE |
| QCC34408.1 | mxiD | 502 | K | QCC34408.1 | mxiD | 542 | K | 12.62 | 1 | TT | Self | 26.11 | Z14 | Z1 | TRUE |
| QCC34416.1 | mxiG | 293 | K | QCC34416.1 | mxiG | 355 | S | 12.61 | 2 | TT | Self | 12.01 | X11 | X11 | TRUE |
| QCC34408.1 | mxiD | 542 | K | QCC34410.1 | mxiM | 146 | K | 12.6 | 3 | TT | Heteromeric |  |  |  | TRUE |
| QCC34416.1 | mxiG | 2 | S | QCC34416.1 | mxiG | 72 | S | 12.55 | 3 | TT | Self |  |  |  | TRUE |
| QCC34410.1 | mxiM | 115 | K | TagQCC34415.1 | TagMxiH | 2 | A | 12.51 | 3 | TT | Heteromeric |  |  |  | FALSE |
| QCC34408.1 | mxiD | 502 | K | QCC34408.1 | mxiD | 558 | K | 12.49 | 2 | TT | Self |  |  |  | TRUE |
| QCC34416.1 | mxiG | 345 | K | QCC34416.1 | mxiG | 347 | K | 12.47 | 2 | TT | Homomultimeric | 5.76 | X7 | X8 | TRUE |
| QCC34410.1 | mxiM | 77 | K | QCC34410.1 | mxiM | 130 | K | 12.43 | 1 | TT | Self |  |  |  | TRUE |
| QCC34416.1 | mxiG | 268 | K | TagQCC34415.1 | TagMxiH | 2 | A | 12.41 | 1 | TT | Heteromeric |  |  |  | FALSE |
| QCC34416.1 | mxiG | 265 | K | TagQCC34415.1 | TagMxiH | 2 | A | 12.35 | 2 | TT | Heteromeric |  |  |  | FALSE |
| QCC34408.1 | mxiD | 189 | K | QCC34408.1 | mxiD | 542 | K | 12.35 | 1 | TT | Self | 33.81 | Z1 | Z3 | TRUE |
| QCC34408.1 | mxiD | 542 | K | QCC34410.1 | mxiM | 52 | K | 12.26 | 1 | TT | Heteromeric |  |  |  | TRUE |
| QCC34429.1 | VirB | 48 | K | QCC34429.1 | VirB | 70 | K | 12.25 | 1 | TT | Self |  |  |  | TRUE |
| QCC34429.1 | VirB | 48 | K | QCC34429.1 | VirB | 157 | K | 12.22 | 2 | TT | Self |  |  |  | TRUE |
| QCC34408.1 | mxiD | 148 | K | QCC34408.1 | mxiD | 222 | K | 12.15 | 1 | TT | Self | 23.59 | Z9 | Z9 | TRUE |
| QCC34408.1 | mxiD | 542 | K | TagQCC34415.1 | TagMxiH | 2 | A | 12.15 | 2 | TT | Heteromeric |  |  |  | FALSE |
| QCC34414.1 | mxiI | 33 | K | QCC34416.1 | mxiG | 347 | K | 12.11 | 1 | TT | Heteromeric | 33.05 | N | X12 | TRUE |
| QCC34414.1 | mxiI | 1 | M | QCC34414.1 | mxiI | 16 | S | 12.07 | 1 | TT | Self |  |  |  | TRUE |
| QCC34414.1 | mxiI | 24 | S | TagQCC34415.1 | TagMxiH | 2 | A | 12.07 | 1 | TT | Heteromeric |  |  |  | FALSE |
| TagQCC34415.1 | TagMxiH | 2 | A | TagQCC34415.1 | TagMxiH | 67 | S | 12.07 | 1 | TT | Self |  |  |  | FALSE |
| QCC34408.1 | mxiD | 189 | K | QCC34408.1 | mxiD | 247 | S | 12.05 | 2 | TT | Self |  |  |  | TRUE |
| QCC34413.1 | mxiJ | 143 | K | QCC34416.1 | mxiG | 347 | K | 12.04 | 4 | TT | Heteromeric | 18.89 | Y16 | X19 | TRUE |
| QCC34410.1 | mxiM | 115 | K | QCC34410.1 | mxiM | 144 | K | 12 | 8 | TT | Self |  |  |  | TRUE |
| QCC34408.1 | mxiD | 456 | K | TagQCC34415.1 | TagMxiH | 54 | K | 11.96 | 1 | TT | Heteromeric | 12.93 | Z0 | d | TRUE |
| QCC34416.1 | mxiG | 1 | M | QCC34416.1 | mxiG | 7 | S | 11.95 | 2 | TT | Homomultimeric |  |  |  | TRUE |
| TagQCC34415.1 | TagMxiH | 68 | K | TagQCC34415.1 | TagMxiH | 84 | K | 11.92 | 1 | TT | Self | 11.85 | S | k | TRUE |
| QCC34408.1 | mxiD | 363 | K | QCC34408.1 | mxiD | 502 | K | 11.92 | 3 | TT | Self | 9.9 | Z3 | Z1 | TRUE |
| QCC34410.1 | mxiM | 82 | K | QCC34410.1 | mxiM | 115 | K | 11.9 | 1 | TT | Self |  |  |  | TRUE |
| QCC34408.1 | mxiD | 175 | S | TagQCC34415.1 | TagMxiH | 2 | A | 11.9 | 1 | TT | Heteromeric |  |  |  | FALSE |
| QCC34416.1 | mxiG | 240 | K | QCC34416.1 | mxiG | 252 | K | 11.87 | 7 | TT | Homomultimeric | 21.1 | X23 | X20 | TRUE |
| QCC34429.1 | VirB | 17 | K | QCC34429.1 | VirB | 130 | K | 11.76 | 6 | TT | Self |  |  |  | TRUE |
| QCC34416.1 | mxiG | 151 | K | QCC34416.1 | mxiG | 195 | K | 11.71 | 1 | TT | Self | 9.97 | X19 | X19 | TRUE |
| QCC34410.1 | mxiM | 77 | K | QCC34410.1 | mxiM | 99 | K | 11.68 | 4 | TT | Self |  |  |  | TRUE |
| QCC34414.1 | mxiI | 46 | S | TagQCC34415.1 | TagMxiH | 2 | A | 11.67 | 1 | TT | Heteromeric |  |  |  | FALSE |
| QCC34408.1 | mxiD | 187 | K | QCC34408.1 | mxiD | 189 | K | 11.67 | 3 | TT | Homomultimeric | 20.15 | Z8 | Z7 | TRUE |
| QCC34410.1 | mxiM | 115 | K | QCC34410.1 | mxiM | 130 | K | 11.62 | 2 | TT | Self |  |  |  | TRUE |
| QCC34416.1 | mxiG | 347 | K | TagQCC34415.1 | TagMxiH | 11 | K | 11.61 | 2 | TT | Heteromeric |  |  |  | FALSE |
| QCC34416.1 | mxiG | 5 | K | QCC34416.1 | mxiG | 72 | S | 11.6 | 2 | TT | Self |  |  |  | TRUE |
| QCC34410.1 | mxiM | 99 | K | QCC34410.1 | mxiM | 115 | K | 11.55 | 3 | TT | Self |  |  |  | TRUE |
| TagQCC34415.1 | TagMxiH | 54 | K | TagQCC34415.1 | TagMxiH | 84 | K | 11.48 | 2 | TT | Self | 19.77 | i | d | TRUE |
| QCC34416.1 | mxiG | 282 | K | QCC34416.1 | mxiG | 293 | K | 11.46 | 2 | TT | Self | 10.05 | X2 | X2 | TRUE |
| TagQCC34415.1 | TagMxiH | 11 | K | TagQCC34415.1 | TagMxiH | 54 | K | 11.45 | 4 | TT | Self |  |  |  | FALSE |
| TagQCC34415.1 | TagMxiH | 40 | T | TagQCC34415.1 | TagMxiH | 54 | K | 11.43 | 1 | TT | Self | 9.57 | o | c | TRUE |
| QCC34414.1 | mxiI | 1 | M | QCC34414.1 | mxiI | 31 | S | 11.38 | 1 | TT | Self |  |  |  | TRUE |
| QCC34416.1 | mxiG | 347 | K | QCC34416.1 | mxiG | 362 | K | 11.38 | 2 | TT | Self | 11.12 | X10 | X10 | TRUE |
| QCC34408.1 | mxiD | 542 | K | QCC34410.1 | mxiM | 127 | K | 11.26 | 5 | TT | Heteromeric |  |  |  | TRUE |
| QCC34429.1 | VirB | 31 | K | QCC34429.1 | VirB | 130 | K | 11.2 | 5 | TT | Self |  |  |  | TRUE |
| QCC34414.1 | mxiI | 33 | K | TagQCC34415.1 | TagMxiH | 11 | K | 11.17 | 1 | TT | Heteromeric |  |  |  | FALSE |
| QCC34413.1 | mxiJ | 201 | K | QCC34416.1 | mxiG | 195 | K | 11.14 | 3 | TT | Heteromeric |  |  |  | TRUE |
| QCC34408.1 | mxiD | 558 | K | QCC34410.1 | mxiM | 130 | K | 11.13 | 2 | TT | Heteromeric |  |  |  | TRUE |
| QCC34408.1 | mxiD | 64 | K | TagQCC34415.1 | TagMxiH | 11 | K | 11.13 | 1 | TT | Heteromeric |  |  |  | FALSE |
| QCC34408.1 | mxiD | 64 | K | QCC34408.1 | mxiD | 189 | K | 11.09 | 1 | TT | Self | 79.39 | Z0 | Z0 | TRUE |
| QCC34429.1 | VirB | 17 | K | QCC34429.1 | VirB | 31 | K | 11.04 | 2 | TT | Self |  |  |  | TRUE |
| QCC34408.1 | mxiD | 369 | S | QCC34408.1 | mxiD | 501 | Y | 11.02 | 1 | TT | Self | 28.26 | Z1 | Z0 | TRUE |
| QCC34408.1 | mxiD | 369 | S | QCC34408.1 | mxiD | 502 | K | 11.02 | 3 | TT | Self | 25 | Z1 | Z0 | TRUE |
| QCC34414.1 | mxiI | 31 | S | TagQCC34415.1 | TagMxiH | 2 | A | 11.01 | 1 | TT | Heteromeric |  |  |  | FALSE |
| QCC34408.1 | mxiD | 502 | K | QCC34410.1 | mxiM | 43 | K | 11 | 1 | TT | Heteromeric |  |  |  | TRUE |
| QCC34416.1 | mxiG | 268 | K | QCC34416.1 | mxiG | 282 | K | 10.93 | 2 | TT | Self | 29.18 | X2 | X2 | TRUE |
| QCC34406.1 | mxiA | 151 | K | QCC34406.1 | mxiA | 170 | K | 10.9 | 2 | TT | Self |  |  |  | TRUE |
| TagQCC34415.1 | TagMxiH | 2 | A | TagQCC34415.1 | TagMxiH | 72 | Y | 10.87 | 1 | TT | Self |  |  |  | FALSE |
| QCC34408.1 | mxiD | 189 | K | QCC34408.1 | mxiD | 558 | K | 10.82 | 2 | TT | Self |  |  |  | TRUE |
| QCC34414.1 | mxiI | 86 | K | TagQCC34415.1 | TagMxiH | 84 | K | 10.82 | 1 | TT | Heteromeric | 14.16 | R | S | TRUE |
| QCC34416.1 | mxiG | 240 | K | QCC34416.1 | mxiG | 246 | K | 10.81 | 2 | TT | Homomultimeric | 23.12 | X16 | X17 | TRUE |
| QCC34408.1 | mxiD | 364 | K | TagQCC34415.1 | TagMxiH | 2 | A | 10.78 | 1 | TT | Heteromeric |  |  |  | FALSE |
| QCC34410.1 | mxiM | 52 | K | QCC34410.1 | mxiM | 115 | K | 10.77 | 1 | TT | Self |  |  |  | TRUE |
| QCC34416.1 | mxiG | 254 | K | TagQCC34415.1 | TagMxiH | 2 | A | 10.77 | 1 | TT | Heteromeric |  |  |  | FALSE |
| QCC34408.1 | mxiD | 187 | K | TagQCC34415.1 | TagMxiH | 11 | K | 10.73 | 2 | TT | Heteromeric |  |  |  | FALSE |
| QCC34408.1 | mxiD | 362 | K | QCC34410.1 | mxiM | 130 | K | 10.64 | 3 | TT | Heteromeric |  |  |  | TRUE |
| QCC34414.1 | mxiI | 33 | K | TagQCC34415.1 | TagMxiH | 54 | K | 10.62 | 1 | TT | Heteromeric | 42.37 | R | S | TRUE |
| QCC34410.1 | mxiM | 91 | K | QCC34410.1 | mxiM | 127 | K | 10.62 | 3 | TT | Self |  |  |  | TRUE |
| QCC34408.1 | mxiD | 180 | K | TagQCC34415.1 | TagMxiH | 54 | K | 10.6 | 1 | TT | Heteromeric | 15.58 | Z0 | T | TRUE |
| QCC34408.1 | mxiD | 558 | K | QCC34410.1 | mxiM | 77 | K | 10.59 | 2 | TT | Heteromeric |  |  |  | TRUE |
| QCC34408.1 | mxiD | 64 | K | QCC34414.1 | mxiI | 1 | M | 10.56 | 2 | TT | Heteromeric |  |  |  | TRUE |
| QCC34416.1 | mxiG | 293 | K | QCC34416.1 | mxiG | 347 | K | 10.56 | 2 | TT | Self | 20.15 | X5 | X5 | TRUE |
| QCC34408.1 | mxiD | 189 | K | TagQCC34415.1 | TagMxiH | 54 | K | 10.53 | 1 | TT | Heteromeric | 24.23 | Z1 | j | TRUE |
| QCC34408.1 | mxiD | 187 | K | QCC34408.1 | mxiD | 187 | K | 10.49 | 2 | TT | Homomultimeric | 23.2 | Z8 | Z7 | TRUE |
| QCC34410.1 | mxiM | 115 | K | QCC34410.1 | mxiM | 127 | K | 10.33 | 2 | TT | Self |  |  |  | TRUE |
| QCC34416.1 | mxiG | 195 | K | QCC34416.1 | mxiG | 265 | K | 10.32 | 2 | TT | Self | 21.13 | X8 | X8 | TRUE |
| QCC34413.1 | mxiJ | 63 | K | QCC34413.1 | mxiJ | 201 | K | 10.21 | 1 | TT | Self |  |  |  | TRUE |
| QCC34414.1 | mxiI | 1 | M | QCC34416.1 | mxiG | 347 | K | 10.19 | 2 | TT | Heteromeric |  |  |  | TRUE |
| QCC34408.1 | mxiD | 64 | K | QCC34408.1 | mxiD | 222 | K | 10.13 | 1 | TT | Self | 54.35 | Z12 | Z12 | TRUE |
| QCC34414.1 | mxiI | 86 | K | QCC34414.1 | mxiI | 96 | K | 10.13 | 1 | TT | Homomultimeric | 14.02 | M | R | TRUE |
| QCC34416.1 | mxiG | 271 | Y | QCC34416.1 | mxiG | 282 | K | 10.12 | 1 | TT | Self | 20.72 | X10 | X10 | TRUE |
| QCC34416.1 | mxiG | 265 | K | QCC34416.1 | mxiG | 282 | K | 10.1 | 1 | TT | Self | 35.65 | X20 | X23 | TRUE |
| QCC34416.1 | mxiG | 240 | K | QCC34416.1 | mxiG | 273 | S | 10.07 | 2 | TT | Self | 5.43 | X16 | X16 | TRUE |
| QCC34416.1 | mxiG | 5 | K | QCC34416.1 | mxiG | 62 | K | 10.04 | 2 | TT | Self |  |  |  | TRUE |
| QCC34406.1 | mxiA | 170 | K | QCC34416.1 | mxiG | 5 | K | 10.04 | 2 | TT | Heteromeric |  |  |  | TRUE |
| QCC34414.1 | mxiI | 14 | K | TagQCC34415.1 | TagMxiH | 2 | A | 10 | 1 | TT | Heteromeric |  |  |  | FALSE |
| TagQCC34415.1 | TagMxiH | 11 | K | TagQCC34415.1 | TagMxiH | 87 | K | 10 | 1 | TT | Self |  |  |  | FALSE |
| QCC34408.1 | mxiD | 521 | K | QCC34410.1 | mxiM | 77 | K | 9.99 | 1 | TT | Heteromeric |  |  |  | TRUE |
| QCC34408.1 | mxiD | 542 | K | QCC34410.1 | mxiM | 91 | K | 9.77 | 1 | TT | Heteromeric |  |  |  | TRUE |
| QCC34410.1 | mxiM | 77 | K | QCC34410.1 | mxiM | 115 | K | 9.73 | 1 | TT | Self |  |  |  | TRUE |
| QCC34408.1 | mxiD | 148 | K | TagQCC34415.1 | TagMxiH | 2 | A | 9.71 | 1 | TT | Heteromeric |  |  |  | FALSE |
| QCC34408.1 | mxiD | 558 | K | TagQCC34415.1 | TagMxiH | 2 | A | 9.7 | 2 | TT | Heteromeric |  |  |  | FALSE |
| QCC34429.1 | VirB | 70 | K | QCC34429.1 | VirB | 157 | K | 9.68 | 1 | TT | Self |  |  |  | TRUE |
| QCC34408.1 | mxiD | 189 | K | QCC34410.1 | mxiM | 115 | K | 9.64 | 1 | TT | Heteromeric |  |  |  | TRUE |
| QCC34429.1 | VirB | 70 | K | QCC34429.1 | VirB | 130 | K | 9.64 | 1 | TT | Self |  |  |  | TRUE |
| QCC34406.1 | mxiA | 170 | K | QCC34413.1 | mxiJ | 54 | K | 9.55 | 2 | TT | Heteromeric |  |  |  | TRUE |
| QCC34416.1 | mxiG | 268 | K | QCC34416.1 | mxiG | 287 | K | 9.55 | 2 | TT | Self | 28.14 | X20 | X23 | TRUE |
| QCC34408.1 | mxiD | 502 | K | QCC34410.1 | mxiM | 77 | K | 9.49 | 1 | TT | Heteromeric |  |  |  | TRUE |
| QCC34408.1 | mxiD | 521 | K | QCC34414.1 | mxiI | 1 | M | 9.45 | 1 | TT | Heteromeric |  |  |  | TRUE |
| QCC34410.1 | mxiM | 130 | K | TagQCC34415.1 | TagMxiH | 2 | A | 9.42 | 1 | TT | Heteromeric |  |  |  | FALSE |
| QCC34408.1 | mxiD | 171 | K | TagQCC34415.1 | TagMxiH | 11 | K | 9.39 | 1 | TT | Heteromeric |  |  |  | FALSE |
| QCC34416.1 | mxiG | 160 | K | QCC34416.1 | mxiG | 265 | K | 9.36 | 1 | TT | Self | 16.67 | X22 | X22 | TRUE |
| QCC34413.1 | mxiJ | 143 | K | TagQCC34415.1 | TagMxiH | 11 | K | 9.34 | 1 | TT | Heteromeric |  |  |  | FALSE |
| QCC34408.1 | mxiD | 222 | K | QCC34408.1 | mxiD | 254 | S | 9.26 | 1 | TT | Self |  |  |  | TRUE |
| QCC34408.1 | mxiD | 566 | Y | TagQCC34415.1 | TagMxiH | 2 | A | 9.24 | 1 | TT | Heteromeric |  |  |  | FALSE |
| QCC34413.1 | mxiJ | 178 | K | TagQCC34415.1 | TagMxiH | 11 | K | 9.23 | 1 | TT | Heteromeric |  |  |  | FALSE |
| QCC34416.1 | mxiG | 300 | K | QCC34416.1 | mxiG | 313 | K | 9.21 | 1 | TT | Self | 16.2 | X4 | X3 | TRUE |
| QCC34406.1 | mxiA | 170 | K | QCC34416.1 | mxiG | 17 | K | 9.19 | 1 | TT | Heteromeric |  |  |  | TRUE |
| QCC34408.1 | mxiD | 191 | T | TagQCC34415.1 | TagMxiH | 2 | A | 9.17 | 2 | TT | Heteromeric |  |  |  | FALSE |
| QCC34424.1 | ipaB | 157 | K | QCC34424.1 | ipaB | 188 | K | 9.12 | 2 | TT | Self |  |  |  | TRUE |
| QCC34406.1 | mxiA | 170 | K | QCC34416.1 | mxiG | 268 | K | 9.03 | 1 | TT | Heteromeric |  |  |  | TRUE |
| QCC34408.1 | mxiD | 456 | K | QCC34408.1 | mxiD | 521 | K | 9.02 | 1 | TT | Self | 25 | Z13 | Z14 | TRUE |
| QCC34410.1 | mxiM | 82 | K | QCC34410.1 | mxiM | 127 | K | 8.97 | 1 | TT | Self |  |  |  | TRUE |
| QCC34408.1 | mxiD | 521 | K | QCC34410.1 | mxiM | 127 | K | 8.96 | 2 | TT | Heteromeric |  |  |  | TRUE |
| QCC34408.1 | mxiD | 64 | K | QCC34414.1 | mxiI | 33 | K | 8.95 | 1 | TT | Heteromeric | 16.18 | Z6 | P | TRUE |
| QCC34429.1 | VirB | 121 | K | QCC34429.1 | VirB | 157 | K | 8.91 | 1 | TT | Self |  |  |  | TRUE |
| QCC34410.1 | mxiM | 91 | K | QCC34410.1 | mxiM | 139 | K | 8.88 | 1 | TT | Self |  |  |  | TRUE |
| QCC34413.1 | mxiJ | 54 | K | QCC34413.1 | mxiJ | 58 | S | 8.78 | 3 | TT | Homomultimeric | 16.15 | Y5 | Y6 | TRUE |
| QCC34408.1 | mxiD | 362 | K | QCC34408.1 | mxiD | 558 | K | 8.78 | 2 | TT | Self |  |  |  | TRUE |
| QCC34400.1 | Spa24 | 105 | K | QCC34416.1 | mxiG | 362 | K | 8.76 | 2 | TT | Heteromeric | 16.69 | B | X0 | TRUE |
| QCC34408.1 | mxiD | 180 | K | QCC34408.1 | mxiD | 189 | K | 8.74 | 1 | TT | Self | 28.32 | Z13 | Z13 | TRUE |
| QCC34400.1 | Spa24 | 84 | T | TagQCC34415.1 | TagMxiH | 87 | K | 8.7 | 1 | TT | Heteromeric |  |  |  | TRUE |
| TagQCC34415.1 | TagMxiH | 54 | K | TagQCC34415.1 | TagMxiH | 70 | S | 8.67 | 1 | TT | Self | 14.96 | p | u | TRUE |
| TagQCC34415.1 | TagMxiH | 2 | A | TagQCC34415.1 | TagMxiH | 17 | S | 8.62 | 1 | TT | Self |  |  |  | FALSE |
| QCC34408.1 | mxiD | 222 | K | TagQCC34415.1 | TagMxiH | 11 | K | 8.62 | 2 | TT | Heteromeric |  |  |  | FALSE |
| QCC34408.1 | mxiD | 174 | S | TagQCC34415.1 | TagMxiH | 2 | A | 8.61 | 1 | TT | Heteromeric |  |  |  | FALSE |
| QCC34416.1 | mxiG | 5 | K | QCC34416.1 | mxiG | 7 | S | 8.6 | 1 | TT | Homomultimeric |  |  |  | TRUE |
| QCC34406.1 | mxiA | 170 | K | QCC34416.1 | mxiG | 195 | K | 8.58 | 1 | TT | Heteromeric |  |  |  | TRUE |
| QCC34413.1 | mxiJ | 133 | Y | QCC34413.1 | mxiJ | 143 | K | 8.56 | 2 | TT | Self | 10.25 | Y22 | Y22 | TRUE |
| QCC34408.1 | mxiD | 298 | K | TagQCC34415.1 | TagMxiH | 11 | K | 8.55 | 3 | TT | Heteromeric |  |  |  | FALSE |
| QCC34408.1 | mxiD | 247 | S | TagQCC34415.1 | TagMxiH | 2 | A | 8.52 | 1 | TT | Heteromeric |  |  |  | FALSE |
| QCC34408.1 | mxiD | 180 | K | TagQCC34415.1 | TagMxiH | 11 | K | 8.45 | 1 | TT | Heteromeric |  |  |  | FALSE |
| TagQCC34415.1 | TagMxiH | 54 | K | TagQCC34415.1 | TagMxiH | 72 | Y | 8.38 | 1 | TT | Self | 11.89 | p | u | TRUE |
| QCC34416.1 | mxiG | 273 | S | QCC34416.1 | mxiG | 282 | K | 8.25 | 2 | TT | Self | 14.67 | X2 | X2 | TRUE |
| QCC34408.1 | mxiD | 38 | Y | QCC34416.1 | mxiG | 293 | K | 8.21 | 7 | TT | Heteromeric | 22.55 | Z10 | X3 | TRUE |
| QCC34408.1 | mxiD | 189 | K | QCC34408.1 | mxiD | 251 | S | 8.2 | 1 | TT | Self |  |  |  | TRUE |
| QCC34400.1 | Spa24 | 100 | K | TagQCC34415.1 | TagMxiH | 2 | A | 8.2 | 1 | TT | Heteromeric |  |  |  | FALSE |
| QCC34410.1 | mxiM | 75 | Y | QCC34410.1 | mxiM | 115 | K | 8.13 | 1 | TT | Self |  |  |  | TRUE |
| QCC34429.1 | VirB | 130 | K | QCC34429.1 | VirB | 157 | K | 8.13 | 1 | TT | Self |  |  |  | TRUE |
| QCC34400.1 | Spa24 | 86 | S | TagQCC34415.1 | TagMxiH | 2 | A | 8.08 | 1 | TT | Heteromeric |  |  |  | FALSE |
| QCC34410.1 | mxiM | 121 | K | QCC34410.1 | mxiM | 130 | K | 8.07 | 1 | TT | Self |  |  |  | TRUE |
| QCC34408.1 | mxiD | 64 | K | QCC34410.1 | mxiM | 115 | K | 7.95 | 1 | TT | Heteromeric |  |  |  | TRUE |
| TagQCC34415.1 | TagMxiH | 72 | Y | TagQCC34415.1 | TagMxiH | 84 | K | 7.94 | 1 | TT | Self | 13.74 | f | m | TRUE |
| QCC34400.1 | Spa24 | 100 | K | QCC34408.1 | mxiD | 64 | K | 7.91 | 1 | TT | Heteromeric | 13.32 | A | Z10 | TRUE |
| QCC34408.1 | mxiD | 222 | K | QCC34408.1 | mxiD | 558 | K | 7.9 | 2 | TT | Self |  |  |  | TRUE |
| QCC34410.1 | mxiM | 77 | K | QCC34410.1 | mxiM | 127 | K | 7.81 | 1 | TT | Self |  |  |  | TRUE |
| TagQCC34415.1 | TagMxiH | 38 | T | TagQCC34415.1 | TagMxiH | 54 | K | 7.8 | 1 | TT | Self | 5.2 | u | p | TRUE |
| QCC34408.1 | mxiD | 64 | K | QCC34408.1 | mxiD | 64 | K | 7.77 | 2 | TT | Homomultimeric | 18.22 | Z12 | Z11 | TRUE |
| QCC34408.1 | mxiD | 523 | S | TagQCC34415.1 | TagMxiH | 2 | A | 7.72 | 3 | TT | Heteromeric |  |  |  | FALSE |
| QCC34408.1 | mxiD | 180 | K | QCC34408.1 | mxiD | 187 | K | 7.71 | 1 | TT | Homomultimeric | 29.76 | Z8 | Z7 | TRUE |
| QCC34410.1 | mxiM | 118 | S | QCC34410.1 | mxiM | 121 | K | 7.56 | 1 | TT | Homomultimeric |  |  |  | TRUE |
| QCC34408.1 | mxiD | 533 | S | QCC34408.1 | mxiD | 542 | K | 7.56 | 1 | TT | Self | 14.94 | Z1 | Z3 | TRUE |
| QCC34400.1 | Spa24 | 100 | K | QCC34414.1 | mxiI | 33 | K | 7.55 | 1 | TT | Heteromeric | 19.95 | E | N | TRUE |
| QCC34408.1 | mxiD | 175 | S | QCC34408.1 | mxiD | 222 | K | 7.47 | 1 | TT | Self |  |  |  | TRUE |
| QCC34408.1 | mxiD | 38 | Y | QCC34416.1 | mxiG | 300 | K | 7.46 | 4 | TT | Heteromeric | 33.46 | Z15 | X11 | TRUE |
| QCC34406.1 | mxiA | 170 | K | QCC34413.1 | mxiJ | 49 | K | 7.45 | 1 | TT | Heteromeric |  |  |  | TRUE |
| QCC34410.1 | mxiM | 82 | K | QCC34410.1 | mxiM | 121 | K | 7.37 | 1 | TT | Self |  |  |  | TRUE |
| QCC34408.1 | mxiD | 544 | T | QCC34410.1 | mxiM | 144 | K | 7.28 | 3 | TT | Heteromeric |  |  |  | TRUE |
| QCC34408.1 | mxiD | 542 | K | QCC34408.1 | mxiD | 549 | S | 7.26 | 1 | TT | Homomultimeric |  |  |  | TRUE |
| QCC34408.1 | mxiD | 362 | K | QCC34408.1 | mxiD | 500 | K | 7.25 | 1 | TT | Self | 11.17 | Z1 | Z0 | TRUE |
| QCC34416.1 | mxiG | 160 | K | QCC34416.1 | mxiG | 195 | K | 7.24 | 1 | TT | Self | 11.49 | X23 | X20 | TRUE |
| QCC34408.1 | mxiD | 542 | K | QCC34408.1 | mxiD | 558 | K | 7.11 | 1 | TT | Homomultimeric |  |  |  | TRUE |
| QCC34416.1 | mxiG | 293 | K | QCC34416.1 | mxiG | 352 | S | 7 | 2 | TT | Self | 15.47 | X22 | X22 | TRUE |
| QCC34414.1 | mxiI | 38 | K | TagQCC34415.1 | TagMxiH | 2 | A | 6.96 | 1 | TT | Heteromeric |  |  |  | FALSE |
| QCC34413.1 | mxiJ | 49 | K | QCC34413.1 | mxiJ | 58 | S | 6.94 | 1 | TT | Homomultimeric | 14.3 | Y22 | Y1 | TRUE |
| QCC34408.1 | mxiD | 259 | S | TagQCC34415.1 | TagMxiH | 2 | A | 6.87 | 1 | TT | Heteromeric |  |  |  | FALSE |
| QCC34413.1 | mxiJ | 49 | K | QCC34413.1 | mxiJ | 49 | K | 6.87 | 1 | TT | Homomultimeric | 18.21 | Y1 | Y22 | TRUE |
| TagQCC34415.1 | TagMxiH | 2 | A | TagQCC34415.1 | TagMxiH | 26 | T | 6.78 | 1 | TT | Self |  |  |  | FALSE |
| QCC34400.1 | Spa24 | 84 | T | TagQCC34415.1 | TagMxiH | 2 | A | 6.74 | 1 | TT | Heteromeric |  |  |  | FALSE |
| QCC34408.1 | mxiD | 369 | S | QCC34408.1 | mxiD | 542 | K | 6.72 | 2 | TT | Self | 14.39 | Z1 | Z4 | TRUE |
| QCC34408.1 | mxiD | 130 | K | TagQCC34415.1 | TagMxiH | 2 | A | 6.68 | 1 | TT | Heteromeric |  |  |  | FALSE |
| QCC34400.1 | Spa24 | 105 | K | QCC34400.1 | Spa24 | 126 | K | 6.67 | 1 | TT | Self |  |  |  | TRUE |
| QCC34414.1 | mxiI | 46 | S | TagQCC34415.1 | TagMxiH | 11 | K | 6.67 | 1 | TT | Heteromeric |  |  |  | FALSE |
| QCC34408.1 | mxiD | 364 | K | QCC34410.1 | mxiM | 77 | K | 6.64 | 1 | TT | Heteromeric |  |  |  | TRUE |
| TagQCC34415.1 | TagMxiH | 11 | K | TagQCC34415.1 | TagMxiH | 57 | S | 6.57 | 3 | TT | Self |  |  |  | FALSE |
| QCC34414.1 | mxiI | 1 | M | QCC34414.1 | mxiI | 25 | S | 6.57 | 1 | TT | Self |  |  |  | TRUE |
| QCC34400.1 | Spa24 | 126 | K | TagQCC34415.1 | TagMxiH | 2 | A | 6.42 | 1 | TT | Heteromeric |  |  |  | FALSE |
| QCC34416.1 | mxiG | 252 | K | QCC34416.1 | mxiG | 265 | K | 6.42 | 1 | TT | Self | 15.64 | X4 | X4 | TRUE |
| QCC34408.1 | mxiD | 369 | S | QCC34408.1 | mxiD | 543 | T | 6.4 | 1 | TT | Self | 10.61 | Z1 | Z4 | TRUE |
| QCC34408.1 | mxiD | 64 | K | QCC34408.1 | mxiD | 68 | K | 6.38 | 2 | TT | Homomultimeric | 17.03 | Z4 | Z3 | TRUE |
| QCC34408.1 | mxiD | 544 | T | QCC34410.1 | mxiM | 146 | K | 6.32 | 5 | TT | Heteromeric |  |  |  | TRUE |
| QCC34408.1 | mxiD | 543 | T | QCC34410.1 | mxiM | 144 | K | 6.32 | 2 | TT | Heteromeric |  |  |  | TRUE |
| QCC34408.1 | mxiD | 543 | T | QCC34410.1 | mxiM | 146 | K | 6.32 | 3 | TT | Heteromeric |  |  |  | TRUE |
| QCC34410.1 | mxiM | 121 | K | QCC34410.1 | mxiM | 127 | K | 6.25 | 1 | TT | Homomultimeric |  |  |  | TRUE |
| QCC34407.1 | MxiC | 66 | K | QCC34416.1 | mxiG | 268 | K | 6.25 | 2 | TT | Heteromeric |  |  |  | TRUE |
| QCC34408.1 | mxiD | 544 | T | QCC34410.1 | mxiM | 139 | K | 6.22 | 3 | TT | Heteromeric |  |  |  | TRUE |
| QCC34406.1 | mxiA | 170 | K | QCC34416.1 | mxiG | 254 | K | 6.17 | 1 | TT | Heteromeric |  |  |  | TRUE |
| QCC34414.1 | mxiI | 1 | M | QCC34416.1 | mxiG | 282 | K | 6.13 | 1 | TT | Heteromeric |  |  |  | TRUE |
| QCC34416.1 | mxiG | 353 | K | QCC34416.1 | mxiG | 369 | K | 6.12 | 1 | TT | Self |  |  |  | TRUE |
| QCC34408.1 | mxiD | 523 | S | TagQCC34415.1 | TagMxiH | 11 | K | 6.09 | 2 | TT | Heteromeric |  |  |  | FALSE |
| QCC34408.1 | mxiD | 187 | K | TagQCC34415.1 | TagMxiH | 54 | K | 6.04 | 1 | TT | Heteromeric | 22.12 | Z1 | j | TRUE |
| QCC34408.1 | mxiD | 148 | K | QCC34408.1 | mxiD | 187 | K | 6.04 | 1 | TT | Self | 42.55 | Z13 | Z14 | TRUE |
| QCC34416.1 | mxiG | 204 | K | QCC34416.1 | mxiG | 246 | K | 5.97 | 1 | TT | Self | 29.15 | X9 | X9 | FALSE |
| QCC34420.1 | icsB | 179 | Y | TagQCC34415.1 | TagMxiH | 1 | M | 5.91 | 2 | TT | Heteromeric |  |  |  | FALSE |
| TagQCC34415.1 | TagMxiH | 2 | A | TagQCC34415.1 | TagMxiH | 82 | T | 5.89 | 1 | TT | Self |  |  |  | FALSE |
| QCC34410.1 | mxiM | 127 | K | QCC34410.1 | mxiM | 146 | K | 5.8 | 2 | TT | Self |  |  |  | FALSE |
| QCC34406.1 | mxiA | 543 | S | QCC34419.1 | ipgD | 40 | K | 5.8 | 2 | TT | Heteromeric |  |  |  | FALSE |
| QCC34408.1 | mxiD | 363 | K | QCC34408.1 | mxiD | 542 | K | 5.78 | 2 | TT | Self | 21.69 | Z15 | Z1 | FALSE |
| QCC34416.1 | mxiG | 195 | K | QCC34416.1 | mxiG | 293 | K | 5.71 | 1 | TT | Self | 54.65 | X20 | X23 | FALSE |
| TagQCC34415.1 | TagMxiH | 11 | K | TagQCC34415.1 | TagMxiH | 82 | T | 5.71 | 2 | TT | Self |  |  |  | FALSE |
| QCC34429.1 | VirB | 116 | T | TagQCC34415.1 | TagMxiH | 1 | M | 5.7 | 3 | TT | Heteromeric |  |  |  | FALSE |
| QCC34410.1 | mxiM | 134 | T | QCC34410.1 | mxiM | 144 | K | 5.62 | 1 | TT | Homomultimeric |  |  |  | FALSE |
| QCC34408.1 | mxiD | 543 | T | QCC34410.1 | mxiM | 139 | K | 5.55 | 2 | TT | Heteromeric |  |  |  | FALSE |
| QCC34408.1 | mxiD | 222 | K | QCC34408.1 | mxiD | 467 | K | 5.5 | 1 | TT | Self | 51.06 | Z1 | Z0 | FALSE |
| QCC34416.1 | mxiG | 282 | K | QCC34416.1 | mxiG | 300 | K | 5.34 | 1 | TT | Self | 9.43 | X23 | X20 | FALSE |
| TagQCC34415.1 | TagMxiH | 11 | K | TagQCC34415.1 | TagMxiH | 33 | T | 5.22 | 1 | TT | Self |  |  |  | FALSE |
| QCC34408.1 | mxiD | 187 | K | QCC34408.1 | mxiD | 198 | Y | 5.2 | 1 | TT | Homomultimeric | 22.54 | Z8 | Z7 | FALSE |
| QCC34416.1 | mxiG | 282 | K | QCC34416.1 | mxiG | 292 | Y | 5.19 | 1 | TT | Self | 8.72 | X1 | X1 | FALSE |
| QCC34416.1 | mxiG | 246 | K | QCC34416.1 | mxiG | 293 | K | 5.15 | 1 | TT | Self | 35.61 | X20 | X20 | FALSE |
| QCC34416.1 | mxiG | 293 | K | QCC34416.1 | mxiG | 356 | Y | 5.08 | 1 | TT | Self | 10.87 | X11 | X11 | FALSE |
| QCC34408.1 | mxiD | 148 | K | QCC34408.1 | mxiD | 174 | S | 5.06 | 1 | TT | Self |  |  |  | FALSE |
| QCC34416.1 | mxiG | 304 | S | QCC34416.1 | mxiG | 353 | K | 5.05 | 1 | TT | Self | 11.78 | X2 | X2 | FALSE |
| QCC34410.1 | mxiM | 99 | K | QCC34410.1 | mxiM | 127 | K | 4.96 | 1 | TT | Self |  |  |  | FALSE |
| QCC34410.1 | mxiM | 127 | K | QCC34410.1 | mxiM | 144 | K | 4.94 | 2 | TT | Self |  |  |  | FALSE |
| QCC34416.1 | mxiG | 293 | K | QCC34416.1 | mxiG | 304 | S | 4.89 | 2 | TT | Self | 5.21 | X15 | X15 | FALSE |
| QCC34413.1 | mxiJ | 178 | K | QCC34416.1 | mxiG | 353 | K | 4.87 | 2 | TT | Heteromeric | 18.92 | Y13 | X17 | FALSE |
| QCC34410.1 | mxiM | 70 | K | QCC34410.1 | mxiM | 130 | K | 4.82 | 1 | TT | Self |  |  |  | FALSE |
| QCC34416.1 | mxiG | 245 | S | QCC34416.1 | mxiG | 248 | T | 4.67 | 1 | TT | Self | 8.15 | X17 | X17 | FALSE |
| QCC34429.1 | VirB | 48 | K | QCC34429.1 | VirB | 145 | K | 4.61 | 1 | TT | Self |  |  |  | FALSE |
| QCC34416.1 | mxiG | 282 | K | QCC34416.1 | mxiG | 353 | K | 4.49 | 1 | TT | Self | 18.6 | X4 | X5 | FALSE |
| QCC34408.1 | mxiD | 189 | K | QCC34408.1 | mxiD | 467 | K | 4.32 | 1 | TT | Self | 19.46 | Z1 | Z0 | FALSE |
| QCC34417.1 | ipgF | 130 | Y | TagQCC34415.1 | TagMxiH | 1 | M | 4.29 | 5 | TT | Heteromeric |  |  |  | FALSE |
| QCC34416.1 | mxiG | 310 | T | QCC34416.1 | mxiG | 347 | K | 4.22 | 1 | TT | Self | 14.95 | X4 | X5 | FALSE |
| QCC34410.1 | mxiM | 76 | S | QCC34410.1 | mxiM | 115 | K | 4.2 | 1 | TT | Self |  |  |  | FALSE |
| QCC34406.1 | mxiA | 641 | K | QCC34424.1 | ipaB | 289 | K | 4.13 | 9 | TT | Heteromeric |  |  |  | FALSE |
| QCC34406.1 | mxiA | 641 | K | QCC34424.1 | ipaB | 290 | S | 4.13 | 7 | TT | Heteromeric |  |  |  | FALSE |
| QCC34424.1 | ipaB | 172 | S | TagQCC34415.1 | TagMxiH | 1 | M | 4.08 | 3 | TT | Heteromeric |  |  |  | FALSE |
| QCC34425.1 | ipaC | 195 | S | QCC34425.1 | ipaC | 238 | S | 3.8 | 1 | TT | Self |  |  |  | FALSE |
| TagQCC34415.1 | TagMxiH | 72 | Y | TagQCC34415.1 | TagMxiH | 87 | K | 3.71 | 1 | TT | Self | 10.73 | U | i | FALSE |
| QCC34416.1 | mxiG | 292 | Y | QCC34416.1 | mxiG | 304 | S | 3.68 | 1 | TT | Self | 5.55 | X6 | X6 | FALSE |
| QCC34420.1 | icsB | 457 | K | QCC34420.1 | icsB | 457 | K | 3.67 | 1 | TT | Homomultimeric |  |  |  | FALSE |
| QCC34397.1 | Spa40 | 229 | K | QCC34397.1 | Spa40 | 245 | K | 3.67 | 1 | TT | Self |  |  |  | FALSE |
| QCC34427.1 | ipaA | 36 | S | QCC34427.1 | ipaA | 231 | S | 3.6 | 1 | TT | Self |  |  |  | FALSE |
| QCC34420.1 | icsB | 61 | K | QCC34420.1 | icsB | 229 | K | 3.52 | 1 | TT | Self |  |  |  | FALSE |
| QCC34420.1 | icsB | 61 | K | QCC34420.1 | icsB | 228 | K | 3.52 | 1 | TT | Self |  |  |  | FALSE |
| TagQCC34415.1 | TagMxiH | 11 | K | TagQCC34415.1 | TagMxiH | 84 | K | 3.49 | 2 | TT | Self |  |  |  | FALSE |
| QCC34424.1 | ipaB | 157 | K | QCC34424.1 | ipaB | 429 | K | 3.48 | 1 | TT | Self |  |  |  | FALSE |
| QCC34407.1 | MxiC | 70 | T | TagQCC34415.1 | TagMxiH | 1 | M | 3.4 | 6 | TT | Heteromeric |  |  |  | FALSE |
| QCC34416.1 | mxiG | 204 | K | QCC34416.1 | mxiG | 347 | K | 3.37 | 1 | TT | Self | 42.6 | X2 | X2 | FALSE |
| QCC34406.1 | mxiA | 133 | S | QCC34406.1 | mxiA | 321 | K | 3.36 | 1 | TT | Self |  |  |  | FALSE |
| QCC34413.1 | mxiJ | 54 | K | QCC34413.1 | mxiJ | 63 | K | 3.2 | 2 | TT | Homomultimeric | 24.42 | Y5 | Y6 | FALSE |
| QCC34416.1 | mxiG | 73 | K | QCC34416.1 | mxiG | 112 | K | 3.09 | 1 | TT | Self |  |  |  | FALSE |
| QCC34429.1 | VirB | 145 | K | QCC34429.1 | VirB | 243 | K | 3.07 | 1 | TT | Self |  |  |  | FALSE |
| QCC34425.1 | ipaC | 70 | S | QCC34425.1 | ipaC | 355 | S | 3.05 | 1 | TT | Self |  |  |  | FALSE |
| QCC34419.1 | ipgD | 129 | K | QCC34419.1 | ipgD | 137 | K | 2.94 | 1 | TT | Self |  |  |  | FALSE |
| TagQCC34415.1 | TagMxiH | 75 | Y | TagQCC34415.1 | TagMxiH | 80 | S | 2.89 | 1 | TT | Self | 8.54 | V | V | FALSE |
| QCC34462.1 | mxiK | 126 | S | QCC34462.1 | mxiK | 162 | Y | 2.83 | 1 | TT | Self |  |  |  | FALSE |
| QCC34419.1 | ipgD | 137 | K | QCC34419.1 | ipgD | 478 | K | 2.79 | 1 | TT | Self |  |  |  | FALSE |
| QCC34419.1 | ipgD | 119 | S | QCC34419.1 | ipgD | 308 | K | 2.75 | 1 | TT | Self |  |  |  | FALSE |
| QCC34427.1 | ipaA | 146 | K | QCC34427.1 | ipaA | 453 | S | 2.69 | 1 | TT | Self |  |  |  | FALSE |
| QCC34402.1 | Spa32 | 72 | K | QCC34402.1 | Spa32 | 220 | T | 2.68 | 2 | TT | Self |  |  |  | FALSE |
| QCC34402.1 | Spa32 | 30 | S | QCC34402.1 | Spa32 | 121 | Y | 2.58 | 1 | TT | Self |  |  |  | FALSE |
| QCC34429.1 | VirB | 48 | K | QCC34429.1 | VirB | 282 | T | 2.56 | 1 | TT | Self |  |  |  | FALSE |

**Sup. Table 2. Potential hydrogen bonds between the S domain and the secretin domain of the neighboring subunits in *Shigella*.**

| **S domain** | **Distance (Å)** | **Secretin domain** |
| --- | --- | --- |
| TYR 525[ OH ] | 3.64 | ARG 462[ O  ] (n+1) |
| TYR 526[ N  ] | 3.13 | SER 468[ O  ] (n+1) |
| TYR 526[ O  ] | 3.20 | SER 468[ N  ] (n+1) |
| TYR 526[ O  ] | 3.62 | SER 468[ OG ](n+1) |
| ASN 527[ OD1] | 2.39 | LYS 467[ NZ ] (n+1) |
| THR 528[ N  ] | 3.66 | GLY 466[ O  ] (n+1) |
| THR 528[ OG1] | 3.00 | SER 468[ OG ] (n+1) |
| ALA 529[ N  ] | 3.19 | GLY 466[ O  ] (n+1) |
| GLU 530[ O  ] | 3.86 | GLN 516[ NE2] (n+1) |
| TYR 531[ OH ] | 3.63 | GLU 302[ OE2] (n+1) |
| TYR 531[ OH ] | 2.19 | SER 304[ OG ] (n+1) |
| LYS 532[ O  ] | 2.33 | ARG 370[ NH1] (n+1) |
| THR 544[ OG1] | 3.77 | GLN 516[ OE1] (n+2) |

**Sup. Table 3. Interface area between the S domain and neighboring subunits.**

|  | Interface area n+1 (Å2) | Interface area n+2 (Å2) |
| --- | --- | --- |
| *Shigella* | 857.0 | 338.2 |
| *Salmonella* | 859.7 | 524.8 |

**Sup. Table 4. Potential hydrogen bonds and salt bridges between the S domain and the secretin domain of the neighboring subunits in *Salmonella*.**

| **S domain** | **Distance (Å)** | **Secretin domain** |
| --- | --- | --- |
| LEU 522[ N  ] | 3.36 | SER 466[ O  ] (n+1) |
| ASP 525[ OD1] | 3.35 | SER 466[ OG ] (n+1) |
| LEU 554[ O  ] | 3.70 | ARG 370[ NH1] (n+1) |
| GLN 547[ NE2] | 3.62 | ASN 506[ O  ] (n+2) |
| SER 537[ O  ] | 2.96 | ASN 477[ ND2] (n+2) |
| SER 537[ OG ] | 3.65 | ASN 477[ ND2] (n+2) |
| SER 541[ OG ] | 2.52 | ASN 506[ ND2] (n+2) |
| ARG 551[ NH1] | 3.91 | ASP 475[ OD2] (n+2) |
| ARG 551[ NH2] | 3.07 | ASP 475[ OD1] (n+2) |
